# Facile Access to Fe(III)-Complexing Cyclic Hydroxamic Acids in a Three-Component Format

**DOI:** 10.3390/molecules24050864

**Published:** 2019-02-28

**Authors:** Evgeny Chupakhin, Olga Bakulina, Dmitry Dar’in, Mikhail Krasavin

**Affiliations:** 1 Institute of Chemistry, Saint Petersburg State University, 199034 Saint Petersburg, Russia; chupakhinevgen@gmail.com (E.C.); o.bakulina@spbu.ru (O.B.); d.dariin@spbu.ru (D.D.); 2 Institute of Living Systems, Immanuel Kant Baltic Federal University, 236016 Kaliningrad, Russia

**Keywords:** iron complexation, cyclic hydroxamic acids, Castagnoli–Cushman reaction, homophthalic acid, hydroxylamine acetate, azeotropic distillation, cyclodehydration

## Abstract

Cyclic hydroxamic acids can be viewed as effective binders of soluble iron and can therefore be useful moieties for employing in compounds to treat iron overload disease. Alternatively, they are analogs of bacterial siderophores (iron-scavenging metabolites) and can find utility in designing antibiotic constructs for targeted delivery. An earlier described three-component variant of the Castagnoli—Cushman reaction of homophthalic acid (via in situ cyclodehydration to the respective anhydride) was extended to involve hydroxylamine in lieu of the amine component of the reaction. Using hydroxylamine acetate and *O*-benzylhydroxylamine was key to the success of this transformation due to greater solubility of the reagents in refluxing toluene (compared to hydrochloride salt). The developed protocol was found suitable for multigram-scale syntheses of *N*-hydroxy- and *N*-(benzyloxy)tetrahydroisoquinolonic acids. The cyclic hydroxamic acids synthesized in the newly developed format have been tested and shown to be efficient ligands for Fe^3+^, which makes them suitable candidates for the above-mentioned applications.

## 1. Introduction

The ability of cyclic hydroxamic acids (*N*-hydroxylactams) to chelate metal ions in general—and Fe^3+^ in particular—defines their utility in drug design [1]. One principal avenue in this regard is based on the recognition of the similarity of synthetic cyclic hydroxamic acids to bacterial siderophores—special metabolites excreted by bacteria to scavenge and deliver iron for the microorganism’s nutritional needs [2]. Conjugating such moieties to antibiotics helps shuttle those inside bacteria and thus overcome resistance of the latter to the antibacterial agent [3]. Besides said application in resistance-free antibiotic design, selective, non-toxic iron chelators are needed as treatments for hereditary iron overload disease [4]. Unfortunately, streamlined and multicomponent methods to prepare *N*-hydroxylactams are lacking. Among the existing synthetic methods for hydroxamic acid, intramolecular nucleophilic cyclization onto *O*-protected acyclic hydroxamic acids [5], nitroso moiety insertion in cyclic ketones [6], and ring-closing methathesis of bis-olefinic hydroxamic acids [7] can be mentioned.

**Figure 1:**
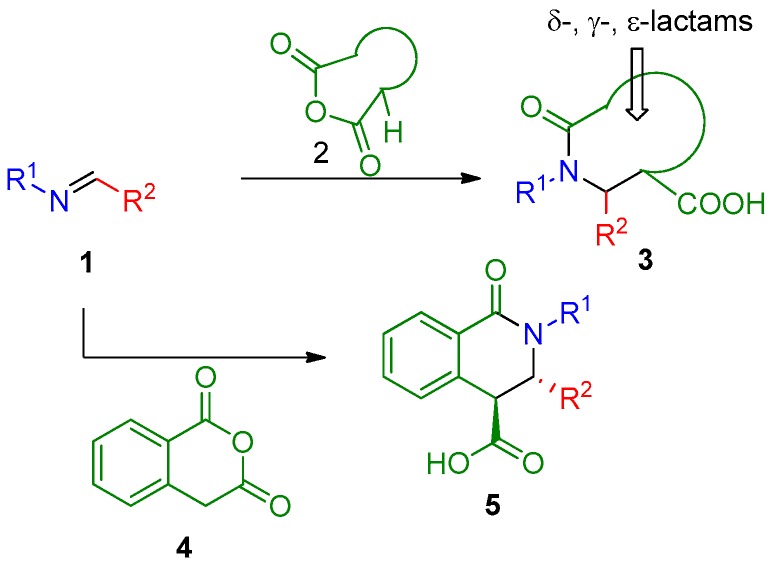
The Castagnoli–Cushman reaction (CCR).

Imines **1** are known to condense with α-C-H anhydrides of dicarboxylic acids **2** to give polysubstituted lactams **3** [8]. In the recent literature, this powerful reaction has been regarded as a name reaction—the Castagnoli–Cushman reaction (CCR) [9]—to acknowledge its origination from the research efforts of Neil Castagnoli and Mark Cushman over 40 years ago [10,11]. Homophthalic anhydride (HPA, **4**) is one of the most frequently employed anhydrides in this reaction, which normally gives rise to *trans*-configured tetrahydroisoquinolonic (THIQ) acids **5** (Figure 1) [12,13]. These have a documented utility in medicinal chemistry (in therapeutic areas such as cancer [14], malaria [15], and diabetes [16]).

**Scheme 1:**
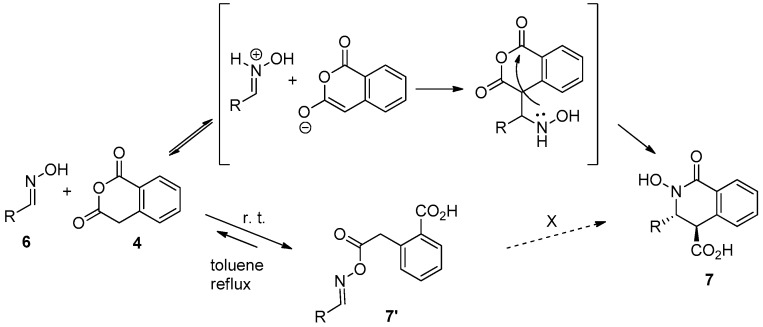
Reaction of oximes **6** with HPA (**4**) and its mechanistic interpretation [15].

Recently, we successfully replaced the imine component in the CCR of **4** with oximes **6** and obtained, after 24 h reflux in toluene, good to excellent yields of the respective *N*-hydroxy THIQ acids **7** [17] which are representative of cyclic hydroxamic acids. The forcing conditions required in order to obtain **7** were in sharp contrast with ambient temperature normally sufficient for the preparation of **5**. This was justified [17] by the need to re-generate HPA (4) from the initial, rapidly formed *O*-acylation product **7′** (Scheme 1).

**Scheme 2:**
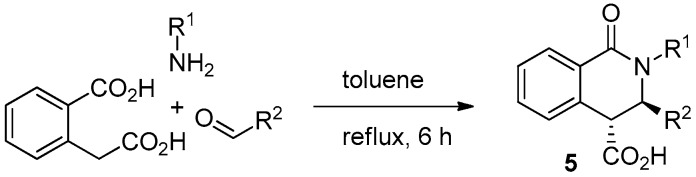
Synthesis of THIQ acids **5** in a three-component format from homophthalic acid [18].

More recently, we employed refluxing toluene for in situ dehydration of homophthalic acid which allowed, for the first time, preparing THIQ acids **5** in a true multicomponent format (i.e., by mixing an amine, an aldehyde and homophthalic acids) without a need for preliminary imine synthesis or the use of hydrolytically prone HPA (Scheme 2) [18].

These findings encouraged us to investigate the preparation of *N*-hydroxy THIQ acids **7** from homophthalic acid and oximes and possible applicability in this case of the same three-component format as presented in Scheme 2. Herein, we present the results of these findings and present characterization of compounds **7** with respect to their iron-binding properties, which validates them as potential candidates for iron overload disease treatment or the design of siderophore-based constructs for antibiotic delivery.

## 2. Results and Discussion

**Scheme 3:**
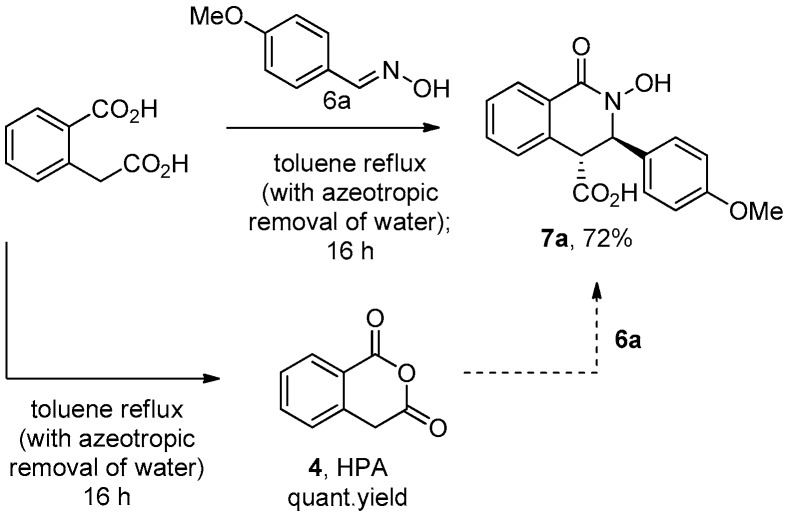
Two-component preparation of **7a** from homophthalic acid.

The initial reaction of oxime **6a** with homophthalic acid in toluene proved rather encouraging and yielded, after 16 h reflux and cooling to room temperature, 72% of the desired product **7a** [17] (Scheme 3). The reaction proceeded via formation of HPA (**4**), which was supported by the results of a separate experiment, where homophthalic acid was refluxed in toluene with azeotropic removal of water to form corresponding anhydride in quantitative yield. This experiment can be also regarded as a new method for the preparation of homophthalic anhydride.

**Scheme 4:**
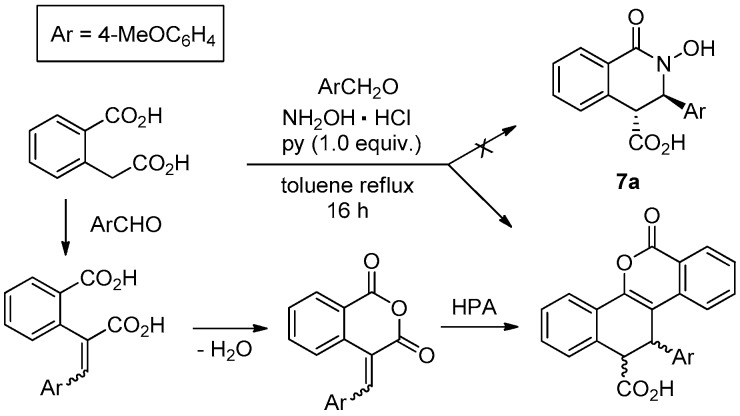
Attempted three-component preparation of **7a** from hydroxylamine hydrochloride (see ref. [19] for more mechanistic insight).

An attempt to perform the same reaction by mixing homophthalic acid with *p*-anisaldehyde and hydroxylamine hydrochloride in presence of pyridine (1 equiv.) did not lead to the formation of **7a**. Instead, the only product discernible by ^1^H-NMR (Nuclear Magnetic Resonance) analysis of the reaction mixture was tetracyclic adduct formed, presumably, via the earlier described [19] Perkin-Michael domino transformation (Scheme 4).

**Scheme 5:**
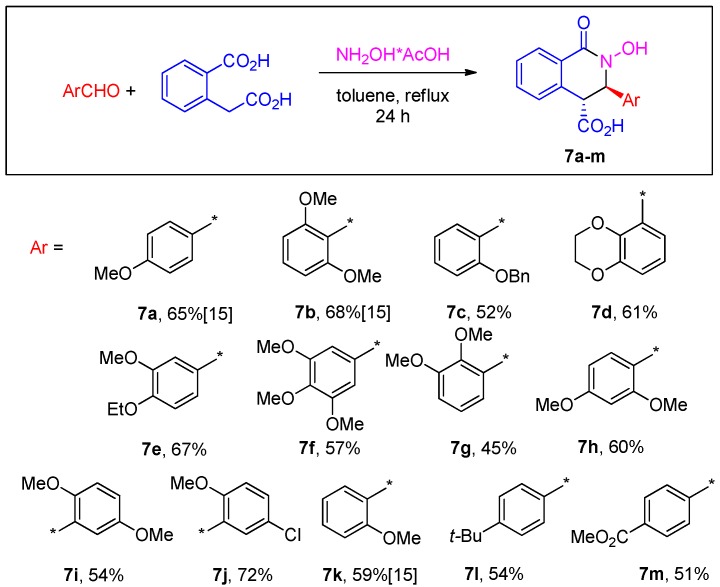
Three-component synthesis of *N*-Hydroxy THIQ acids **7a**–**m**.

The observed complication is likely the result of the inefficient formation of the intermediate oxime **6** in the non-polar reaction medium (due to low solubility in it of hydroxylamine hydrochloride) and the ready participation of free *p*-anisaldehyde in the route leading to tetracyclic adduct. To circumvent this obstacle, we attempted to prepare hydroxylamine acetate and use it in the same transformation. We anticipated that in addition to improved solubility of the acetate salt in toluene, this transformation would not require the use of a base as acetic acid would be conveniently removed from the reaction medium by azeotropic distillation with the solvent. Interestingly, hydroxylamine acetate has been seldom [20] employed for the preparation of oximes despite the obvious advantage of not having to use any base (as is the case with the usual hydroxylamine hydrochloride). The hydrochloride salt of hydroxylamine was converted to its acetate by the action of sodium acetate and reacted with *p*-anisaldehyde and homophthalic acid in refluxing toluene over 24 h. To our delight, on cooling to r. t., a thick precipitate formed which was isolated by filtration to provide 65% yield of compound **7a**. The same reaction was attempted at reflux in benzene and xylenes. While the former conditions led to <15% conversion over 24 h, the latter gave an appreciable amount of *N*-deoxygenation product. Considering this and the results of the reaction monitoring at different time points, 24 h reflux in toluene was considered to be optimal for the preparation of compound **7a**. Thus, these conditions were extended to the preparation of a new series of *N*-hydroxy THIQ acids **7a**–**m** (Scheme 5).

**Scheme 6:**
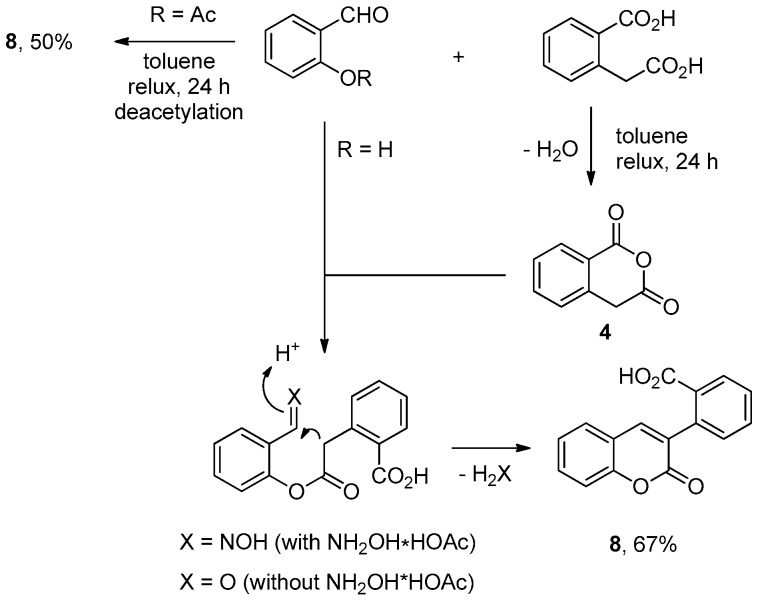
Involvement of unprotected *o*-salicaldehyde in the reaction with homophthalic acid.

An interesting result was obtained in an attempt to involve *o*-salicylaldehyde in the same reaction. When all the aldehyde starting material was consumed (according to HPLC (High Performance Liquid Chromatography) analysis of the reaction mixture), only a trace amount of anticipated compound was detected. The major product identified and isolated from the reaction mixture was coumarin **8**. The same reaction run without hydroxylamine acetate gave identical yield of **8** (67%). It likely that the reaction (described earlier but not interpreted from the mechanistic viewpoint [21]) involves acylation of the phenolic hydroxy group followed by intramolecular Knoevenagel reaction (Scheme 6). Involvement of *O*-acetyl salicylaldehyde in the same reaction led to de-acetylation and the formation of **8** in 50% yield.

The reaction generally worked very well with electron-rich aromatic aldehydes and gave good yields of respective *N*-hydroxy THIQ acids **7a**–**k** (Scheme 5). Substrates without alkoxy groups (such as *tert*-butyl-, methoxycarbonyl-, fluoro-, bromo-, and nitro-benzaldehydes,) surprisingly, gave no desired product under these conditions. Increasing the concentration of reactants from 0.1 M to 1 M and the reaction time from 24 to 48 h, allowed us to slightly increase the reaction scope by involving 4-(*tert*-butyl)benzaldehyde and 4-(methoxycarbonyl)benzaldehyde in the developed approach and to prepare corresponding *N*-hydroxy THIQ acids **7l** and **7m** in good yields (54 and 51% respectively). However, still the reaction was not applicable to more electron-poor substrates.

**Scheme 7:**
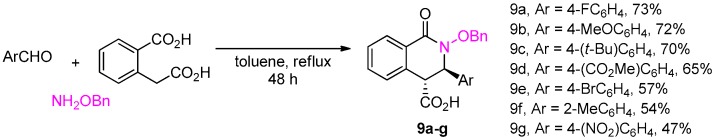
Three-component synthesis of *N*-Benzyloxy THIQ acids **9**.

The ^1^H- and ^13^C-NMR analysis of corresponding reaction mixtures showed that in case of electron-poor aldehydes the major reaction products are nitriles (formed by dehydration of oximes). To prevent this side reaction, we replaced hydroxylamine acetate with *O*-benzylhydroxylamine (Scheme 6). To our delight this allowed to involve previously unreactive electron-poor aldehydes (even 4-fluoro- and 4-nitro-benzaldehydes). Following this new modified protocol (also performed at 1 M concentration of reactants) seven novel *N*-benzyloxy THIQ acid derivatives **9a**–**g** have been prepared in high and good yields (Scheme 7). For all prepared compounds **7a**–**m** and **9a**–**g** *trans*-configuration was concluded from ^3^J_HH_ coupling constant values (~1–2 Hz) between protons at positions C3 and C4, which is consistent with our previous data [17].

**Scheme 8:**
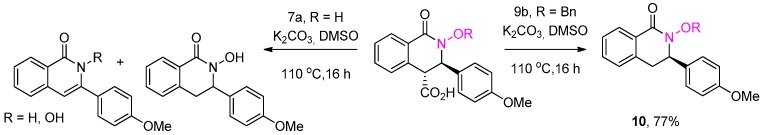
Decarboxylation of compounds **9a** and **9b**.

**Scheme 9:**
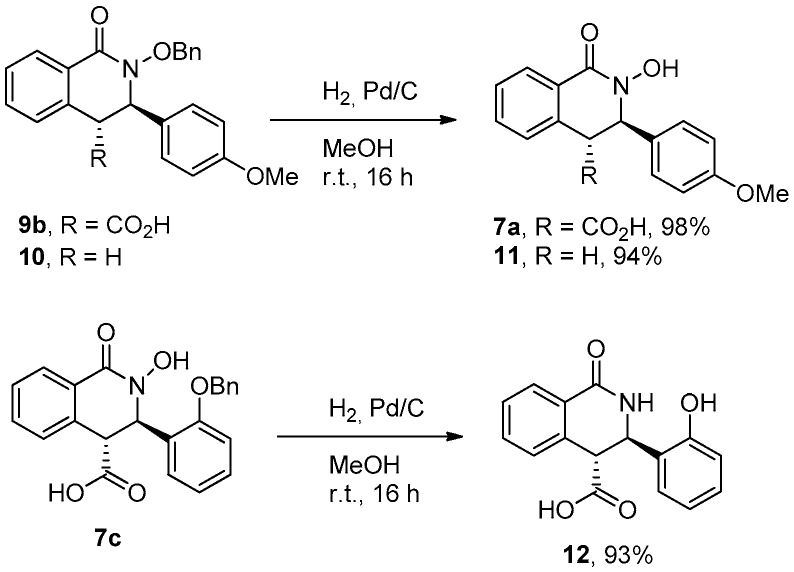
Debenzylation of compounds **9b**, **10**, and **7c**.

Additionally, two types of post-modifications have been investigated for the prepared compounds **7** and **9**: decarboxylation (Scheme 8) and debenzylation (Scheme 9). Attempts to perform decarboxylation of compound **7a** under previously reported conditions [22] lead to formation of complex mixture of products resulting from side reactions of oxidation and dehydration (supported by HRMS (High Resolution Mass Spectrometry) and ^1^H-NMR data) (Scheme 8). At the same time, decarboxylation can be smoothly conducted for *O*-benzylated compound **9b** providing compound **10** in 77% yield, thus demonstrating another advantage of *O*-benzyloxime-based protocol for preparation of *N*-hydroxy THIQ acids derivatives.

We also have investigated the possibility of removal of benzyl protective group from prepared compounds (Scheme 9). *N*-Benzyloxy THIQ acids **9b** and **10** were successfully converted to corresponding OH-hydroxamic acids **7a** and **11** via hydrogenolysis under standard conditions with excellent yields. Surprisingly, attempted *O*-debenzylation of compound **7c** only gave the product of *N*-dehydroxylation **12** (Scheme 9).

**Figure 2:**
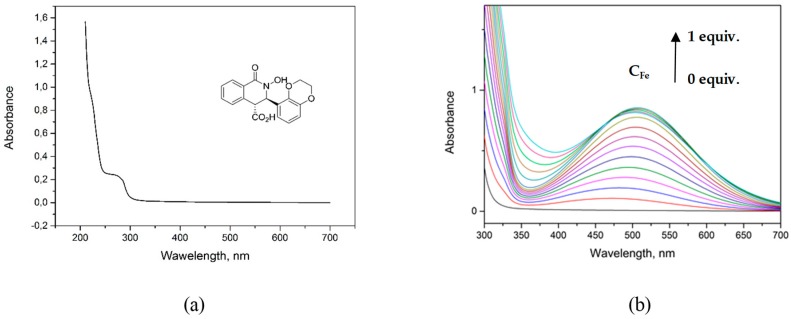
(**a**) UV-Vis absorbance spectrum of compound **7d** in methanol (C = 5 × 10^−5^ M); (**b**) UV-Vis absorbance spectrum of compound **7d** (C_L_ = 5 × 10^−4^ M, aq. MeOH 85%) in the presence of increasing concentration of Fe^3+^ (C_Fe_ = 0.25‒5 × 10^−4^ M).

Compounds **7** synthesized using the developed procedure are direct analogs of bacterial siderophores [23] and, therefore, can be potentially regarded as new agents for iron overload disease therapy and as precursors for the design of “Trojan horse” antibiotics [24] against drug resistant bacteria. Therefore five selected compounds—**7d**,**f**–**h**,**j** which represent different substitution patterns in 3-aryl moiety (4 types of di-substituted and one tri-substituted), were tested for iron(III) binding properties using mole-ratio method to prove this assumption (Figure 2a,b). Upon addition of ferric nitrate to solution of ligand **7** in aqueous methanol a color change from colorless to purple was observed. This corresponds to formation of Fe(III)-7 complex, which is characterized by a new absorption band with maximum around 500 nm (Figure 2b). All ligands **7** in contrast to their complexes with iron do not absorb in the visible region. Representative example of UV-Vis spectrum of free ligand **7** and its changes upon addition of Fe^3+^ ions is shown on Figure 2a for compound **7d**. These spectra for other tested compounds **7** are reported in ESI (Electronic Supporting Information, Figures S1–S3). In all cases two isosbestic points were observed around 470 and 600 nm at C_Fe_ = 0.5‒5 × 10^−4^ M.

**Figure 3:**
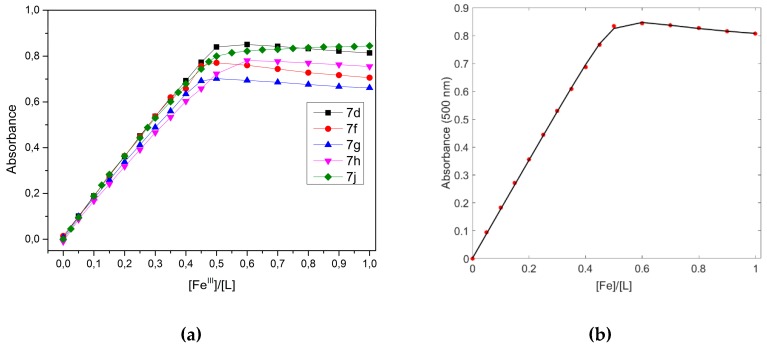
(**a**) Absorbance vs. [Fe^3+^]/[ligand] mole-ratio plot for all Fe^3+^-**7d**,**f**–**h**,**j** complexes; (**b**) Absorbance vs. [Fe^3+^]/[ligand] mole-ratio plot for Fe^3+^-**7d** complexation in aq. MeOH 85% (red asterisks—experimental data, black line—nonlinear curve-fitting according to eqn.3 from Materials and Methods Section).

Applying the mole-ratio method allowed us to determine not only the stoichiometry of observed complexes but also the associated binding constants [17,25]. Absorbance at characteristic wavelength was plotted as function of [Fe^3+^]/[ligand] ratio to give the curves shown in Figure 3a. The binding process involves two equilibria—the first one corresponding to 1:1 complex, and the second one to 1:2 complex. Stepwise formation constants K_1_ and K_2_ were estimated in the range of 10^5^−10^7^ M^−1^ and 10^4^−10^5^ M^−1^, respectively. These values were calculated using computer nonlinear curve-fitting of the absorbance values taken from experimental mole-ratio plots on Figure 3a to previously derived Equations (1)–(3) describing formation of ML_2_ complex (See Material and Methods Section for details). The example of such fitting (compound **7d**) is presented on Figure 3b. The respective plots with curve-fitting for other tested compounds **7f**–**h**,**j** are presented in ESI (Figures S4–S8) The results on spectrophotometric investigation of iron(III) binding properties for compounds **7** are summarized in Table 1. The obtained K*f* values (~10^10^–10^11^ M^−1^) were found to be in accordance with our previous results [17] for similar compounds. No significant dependence of K*_f_* values on substitution pattern was found.

## 3. Materials and Methods

### 3.1. General Considerations

All commercial reagents were used without further purification. NMR spectra were acquired using Bruker Avance III spectrometer (Billerica, MA, USA) (^1^H: 400.13 MHz; ^13^С: 100.61 MHz; chemical shifts are reported as parts per million (*δ*, ppm); solvents—DMSO-*d*_6_ or CDCl_3_, the residual solvent peaks were used as internal standards: 2.50 ppm for ^1^H and 39.52 ppm for ^13^C (DMSO-*d*_6_) or 7.26 ppm for ^1^H and 77.16 ppm for ^13^C (CDCl_3_); multiplicities are abbreviated as follows: s = singlet, d = doublet, t = triplet, q = quartet, m = multiplet, br = broad; coupling constants, *J*, are reported in Hz. Mass spectra were acquired using Bruker microTOF spectrometer (ionization by electrospray, positive ions detection; Billerica, MA, USA). Melting points were determined in open capillary tubes on Stuart SMP50 Automatic Melting Point Apparatus (Stone, UK). Homophthalic acid, hydroxylamine hydrochloride, *O*-benzylhydroxylamine, aldehydes, and Fe(NO_3_)_3_∙9H_2_O were obtained from commercial sources. All reactions were performed in air, unless otherwise noted. Analytical data obtained for compounds **7a**, **7b**, **7k** and **7m** have been consistent with those reported in our previous work [17].

### 3.2. Synthesis

#### 3.2.1. Homophthalic Anhydride 4 (Scheme 3)

Homophthalic acid (150 mg, 0.83 mmol) was refluxed in toluene (15 mL) in a flask equipped a Dean-Stark distilling trap (ISOLAB Laborgeräte GmbH, Wertheim, Germany) for 16 h. After cooling to room temperature, the reaction mixture was cooled to room temperature and concentrated in vacuo to provide pure title compound (135 mg, 0.83 mmol, quant. yield). ^1^H-NMR (400 MHz, CDCl_3_) δ 8.21 (dd, *J* = 7.9, 1.4 Hz, 1H), 7.69 (td, *J* = 7.6, 1.4 Hz, 1H), 7.51 (td, *J* = 7.7, 1.0 Hz, 1H), 7.44–7.30 (m, 1H), 4.14 (s, 2H) [26].

#### 3.2.2. Hydroxylamine Acetate

Hydroxylamine hydrochloride (10.0 g, 144 mmol) was dissolved in deionized water (5 mL). The solution was added, on stirring, to a solution of sodium acetate (11.8 g, 144 mmol) in water (5 mL). The resulting clear solution was concentrated to dryness. The solid residue was suspended in anhydrous methanol (20 mL) and filtered to remove sodium chloride. The filtrate was concentrated in vacuo to give hydroxylamine acetate (13.0 g, 141 mmol, 98%). ^1^H-NMR (400 MHz, DMSO-*d*_6_) δ 8.20 (s, 4H, NH_2_OH, COOH), 1.86 (s, 3H, CH_3_) [27].

#### 3.2.3. General Procedure for the Preparation of *N*-Hydroxy THIQ Acids **7a**–**k**

A mixture of homophthalic acid (180 mg, 1 mmol), hydroxylamine acetate (102 mg, 1.1 mmol) and the respective aldehyde (1.1 mmol) in toluene (10 mL) was heated at reflux for 24 h in a flask equipped a Dean-Stark distilling trap. A thick precipitate which formed on cooling the reaction mixture to −20 °C was isolated by filtration, washed with hexane, and crystallized from aqueous methanol to provide pure title compounds.

*(3R/S,4R/S)-3-(2-(Benzyloxy)phenyl)-2-hydroxy-1-oxo-1,2,3,4-tetrahydroisoquinoline-4-carboxylic acid* (**7c**). Yield 202 mg (52%); white powder, mp 214.3–214.7 °C; ^1^H-NMR (400 MHz, DMSO-*d*_6_) δ 13.04 (br.s, 1H, COOH), 10.23 (s, 1H, N-OH), 8.00–7.97 (m, 1H, CH(Ar)), 7.60 (d, *J* = 7.3 Hz, 2H, 2CH(Ar)), 7.46–7.33 (m, 5H, 5CH(Ar)), 7.29–7.10 (m, 3H, 3CH(Ar)), 6.78 (t, *J* = 7.5 Hz, 1H, CH(Ar)), 6.66 (d, *J* = 7.5 Hz, 1H, CH(Ar)), 5.89 (s, 1H, 3-H), 5.34–5.15 (m, 2H, CH_2_), 4.19 (s, 1H, 4-H). ^13^C-NMR (101 MHz, DMSO-*d*_6_) δ 172.3, 161.1, 155.6, 137.5, 133.3, 132.1, 130.5, 129.3, 128.9, 128.8, 128.3, 128.2, 127.6, 127.0, 126.4, 126.2, 120.7, 113.0, 69.8, 61.0, 49.9. HRMS (ESI), *m/z* calcd for C_23_H_20_NO_5_ [M + H]^+^ 412.1155, found 412.1149.

*(3R/S,4R/S)-3-(2,3-Dihydrobenzo[b][1,4]dioxin-6-yl)-2-hydroxy-1-oxo-1,2,3,4-tetrahydroisoquinoline-4-carboxylic acid* (**7d**). Yield 208 mg (61%); white powder, mp 219.8–220.2 °C. ^1^H-NMR (400 MHz, DMSO-*d*_6_) δ 13.07 (br.s, 1H, COOH), 10.21 (s, 1H, N-OH), 7.97 (d, *J* = 7.0 Hz, 1H, CH(Ar)), 7.42 (p, *J* = 7.4, 7.0 Hz, 1H, CH(Ar)), 7.49–7.36 (m, 2H, 2CH(Ar)). 7.30 (d, *J* = 6.9 Hz, 1H, CH(Ar)), 6.73 (d, *J* = 8.1 Hz, 1H, CH(Ar)), 6.61 (t, *J* = 7.9 Hz, 1H, CH(Ar)), 6.20 (d, *J* = 7.6 Hz, 1H, CH(Ar)), 5.72 (s, 1H, 3-H), 4.46–4.31 (m, 2H, CH_2_), 4.27 (t, *J* = 4.0 Hz, 2H, CH_2_), 4.16 (s, 1H, CH, 4-H). ^13^C-NMR (101 MHz, DMSO-*d*_6_) δ 172.3, 161.1, 143.9, 141.0, 133.4, 132.1, 130.6, 128.9, 128.3, 126.9, 126.8, 120.6, 118.1, 116.9, 64.9, 64.3, 60.6, 49.8. HRMS (ESI), *m/z* calcd for C_18_H_16_NO_6_ [M + H]^+^ 342.0972, found 342.0970.

*(3R/S,4R/S)-3-(2,3-Dihydrobenzo[b][1,4]dioxin-6-yl)-2-hydroxy-1-oxo-1,2,3,4-tetrahydroisoquinoline-4-carboxylic acid* (**7d**). Yield 208 mg (61%); white powder, mp 219.8–220.2 °C. ^1^H-NMR (400 MHz, DMSO-*d*_6_) δ 13.07 (br.s, 1H, COOH), 10.21 (s, 1H, N-OH), 7.97 (d, *J* = 7.0 Hz, 1H, CH(Ar)), 7.42 (p, *J* = 7.4, 7.0 Hz, 1H, CH(Ar)), 7.49–7.36 (m, 2H, 2CH(Ar)). 7.30 (d, *J* = 6.9 Hz, 1H, CH(Ar)), 6.73 (d, *J* = 8.1 Hz, 1H, CH(Ar)), 6.61 (t, *J* = 7.9 Hz, 1H, CH(Ar)), 6.20 (d, *J* = 7.6 Hz, 1H, CH(Ar)), 5.72 (s, 1H, 3-H), 4.46–4.31 (m, 2H, CH_2_), 4.27 (t, *J* = 4.0 Hz, 2H, CH_2_), 4.16 (s, 1H, CH, 4-H). ^13^C-NMR (101 MHz, DMSO-*d*_6_) δ 172.3, 161.1, 143.9, 141.0, 133.4, 132.1, 130.6, 128.9, 128.3, 126.9, 126.8, 120.6, 118.1, 116.9, 64.9, 64.3, 60.6, 49.8. HRMS (ESI), *m/z* calcd for C_18_H_16_NO_6_ [M + H]^+^ 342.0972, found 342.0970.

*(3R/S,4R/S)-3-(4-Ethoxy-3-methoxyphenyl)-2-hydroxy-1-oxo-1,2,3,4-tetrahydroisoquinoline-4-carboxylic acid* (**7e**). Yield 239 mg (67%); white powder, mp 218.7–218.9 °C; ^1^H-NMR (400 MHz, DMSO-*d*_6_) δ 13.04 (s, 1H, COOH), 10.18 (s, 1H, N-OH), 7.94 (d, *J* = 7.3 Hz, 1H, CH(Ar)), 7.49–7.38 (m, 2H, 2CH(Ar)), 7.30 (d, *J* = 7.2 Hz, 1H, CH(Ar)), 6.80 (s, 1H, CH(Ar)), 6.78 (d, *J* = 8.4 Hz, 1H, CH(Ar)), 6.53 (d, *J* = 8.2 Hz, 1H, CH(Ar)), 5.42 (s, 1H, 3-H), 4.28 (d, *J* = 1.8 Hz, 1H, 4-H), 3.91 (q, *J* = 6.9 Hz, 2H, CH_2_), 3.66 (s, 3H, OCH_3_), 1.26 (t, *J* = 7.0 Hz, 3H, CH_3_). ^13^C-NMR (101 MHz, DMSO-*d*_6_) δ 172.3, 160.7, 149.2, 147.8, 133.6, 132.1, 131.3, 130.2, 129.1, 128.2, 126.9, 118.3, 113.0, 110.7, 65.3, 64.1, 55.8, 51.9, 15.2. HRMS (ESI), *m/z* calcd for C_19_H_20_NO_6_ [M + H]^+^ 358.1285, found 358.1300.

*(3R/S,4R/S)-2-Hydroxy-1-oxo-3-(3,4,5-trimethoxyphenyl)-1,2,3,4-tetrahydroisoquinoline-4-carboxylic acid* (**7f**). Yield 212 mg (57%); white powder, mp 220.6–220.8 °C; ^1^H-NMR (400 MHz, DMSO-*d*_6_) δ 13.09 (s, 1H, COOH), 10.24 (s, 1H, N-OH), 7.95 (dd, *J* = 7.4, 1.3 Hz, 1H, CH(Ar)), 7.51–7.39 (m, 2H, 2CH(Ar)), 7.31 (d, *J* = 7.1 Hz, 1H, CH(Ar)), 6.44 (s, 2H, CH(Ar)), 5.44 (s, 1H, 3-H), 4.33 (s, 1H, 4-H), 3.63 (s, 6H, 2OCH_3_), 3.58 (s, 3H, OCH_3_). ^13^C-NMR (101 MHz, DMSO-*d*_6_) δ 172.2, 160.7, 153.2, 137.2, 134.8, 133.7, 132.2, 130.2, 129.0, 128.3, 126.8, 104.1, 65.5, 60.3, 56.2, 51.8. HRMS (ESI), *m/z* calcd for C_19_H_20_NO_7_ [M + H]^+^ 374.1234, found 374.1244

*(3R/S,4R/S)-3-(2,3-Dimethoxyphenyl)-2-hydroxy-1-oxo-1,2,3,4-tetrahydroisoquinoline-4-carboxylic acid* (**7g**). Yield 154 mg (45%); white powder, mp 224.3–224.6 °C; ^1^H-NMR (400 MHz, DMSO-*d*_6_) δ 13.04 (br.s, 1H, COOH), 10.18 (s, 1H, N-OH), 7.94 (d, *J* = 7.1 Hz, 1H, CH(Ar)), 7.50–7.36 (m, 2H, 2CH(Ar)), 7.29 (d, *J* = 7.1 Hz, 1H, CH(Ar)), 6.80 (s, 1H, CH(Ar)) 6.79 (d, *J* = 8.2 Hz, 1H, CH(Ar)), 6.54 (d, *J* = 9.6 Hz, 1H, CH(Ar)), 5.42 (s, 1H, 4-H), 4.28 (s, 1H, 3-H), 3.66 (s, 3H, OCH_3_), 3.65 (s, 3H, OCH_3_). ^13^C-NMR (101 MHz, DMSO-*d*_6_) *δ* 177.1, 165.5, 153.9, 153.4, 138.4, 137.0, 136.1, 135.0, 133.8, 133.0, 131.66, 123.1, 116.7, 115.4, 70.0, 60.6, 60.6, 56.7. HRMS (ESI), *m/z* calcd for C_18_H_18_NO_6_ [M + H]^+^ 344.1129, found 344.1141.

*(3R/S,4R/S)-3-(2,4-Dimethoxyphenyl)-2-hydroxy-1-oxo-1,2,3,4-tetrahydroisoquinoline-4-carboxylic acid* (**7h**). Yield 205 mg (60%); white powder, mp 208.2–208.7 °C; ^1^H-NMR (400 MHz, DMSO-*d*_6_) δ 12.99 (br.s, 1H, COOH), 10.14 (s, 1H, N-OH), 7.99–7.92 (m, 1H, CH(Ar)), 7.41 (td, *J* = 7.7, 6.6, 3.9 Hz, 2H, 2CH(Ar)), 7.26 (dd, *J* = 5.8, 2.8 Hz, 1H, CH(Ar)), 6.59 (d, *J* = 2.2 Hz, 1H, CH(Ar)), 6.50 (d, *J* = 8.5 Hz, 1H, CH(Ar)), 6.33 (dd, *J* = 8.5, 2.2 Hz, 1H, CH(Ar)), 5.66 (s, 1H, 4-H), 4.05 (s, 1H, 3-H), 3.86 (s, 3H, OCH_3_), 3.68 (s, 3H, OCH_3_). ^13^C-NMR (101 MHz, DMSO-*d*_6_) δ 172.5, 161.1, 160.6, 157.6, 133.5, 132.0, 130.4, 128.9, 128.2, 126.9, 126.7, 118.1, 104.7, 99.3, 60.7, 56.2, 55.6, 50.1. HRMS (ESI), m/z calcd for C_18_H_18_NO_6_ [M + H]^+^ 344.1129, found 344.1141.

*(3R/S,4R/S)-3-(2,5-Dimethoxyphenyl)-2-hydroxy-1-oxo-1,2,3,4-tetrahydroisoquinoline-4-carboxylic acid* (**7i**). This product was isolated after washing the precipitate from r.m. with Et_2_O and filtration. Yield 235 mg (54%); white powder, mp 211.6–211.9 °C; ^1^H-NMR (400 MHz, DMSO-*d*_6_) δ 13.06 (br.s, 1H, COOH), 10.23 (s, 1H, N-OH), 8.00–7.94 (m, 1H, CH(Ar)), 7.47–7.38 (m, 1H, CH(Ar)), 7.29–7.25 (m, 1H, CH(Ar)), 6.96 (d, *J* = 8.9 Hz, 1H, CH(Ar)), 6.77 (dd, *J* = 8.9, 3.0 Hz, 1H, CH(Ar)), 6.19 (d, *J* = 2.7 Hz, 1H, CH(Ar)), 5.71 (s, 1H, 3-H), 4.08 (s, 1H, 4-H), 3.83 (s, 3H, OCH_3_), 3.51 (s, 3H, OCH_3_). ^13^C-NMR (101 MHz, DMSO-*d*_6_) δ 172.3, 161.1, 153.2, 150.6, 133.4, 132.2, 130.5, 128.9, 128.3, 127.2, 126.9, 113.6, 112.5, 61.0, 56.5, 55.6, 49.8. HRMS (ESI), *m*/*z* calcd for C_18_H_18_NO_6_ [M + H]^+^ 344.1129, found 344.1143.

*(3R/S,4R/S)-3-(5-Chloro-2-methoxyphenyl)-2-hydroxy-1-oxo-1,2,3,4-tetrahydroisoquinoline-4-carboxylic acid* (**7j**). Yield 249 mg (72%); white powder, mp 229.2–229.5 °C; ^1^H-NMR (400 MHz, DMSO-*d*_6_) δ 13.10 (br.s, 1H, COOH), 10.35 (s, 1H, N-OH), 7.99 (d, *J* = 7.0 Hz, 1H, CH(Ar)), 7.48–7.40 (m, 2H, 2CH(Ar)), 7.31–7.28 (m, 1H, CH(Ar)), 7.27 (d, *J* = 2.6 Hz, 1H, CH(Ar)), 7.08 (d, *J* = 8.8 Hz, 1H, CH(Ar)), 6.58 (s, 1H, CH(Ar)), 5.72 (s, 1H, 3-H), 4.14 (s, 1H, 4-H), 3.89 (s, 3H, OCH_3_). ^13^C-NMR (101 MHz, DMSO-*d*_6_) *δ* 172.15, 161.05, 155.59, 133.20, 132.35, 130.56, 129.08, 128.58, 128.50, 128.11, 127.02, 125.92, 124.34, 113.67, 60.91, 56.66, 49.35. HRMS (ESI), *m/z* calcd for C_17_H_15_ClNO_5_ [M + H]^+^ 348.0633, found 348.0621.

#### 3.2.4. General Procedure for the Preparation of *N*-Hydroxy THIQ Acids **7l**,**m**

A mixture of homophthalic acid (3.6 g, 20 mmol), hydroxylamine acetate (1.86 g, 20 mmol) and the respective aldehyde (20 mmol) in toluene (20 mL) was placed in a pre-heated oil bath and refluxed for 48 h in a flask equipped a Dean-Stark distilling trap. A thick precipitate which formed on cooling the reaction mixture to −20 °C was isolated by filtration, washed with hexane, and crystallized from aqueous methanol to provide pure title compounds.

*(3R/S,4R/S)3-(4-(tert-Butyl)phenyl)-2-hydroxy-1-oxo-1,2,3,4-tetrahydroisoquinoline-4-carboxylic acid* (**7l**). Yield 3.67 g, 54%. White solid, mp 234–236 °C; ^1^H-NMR (400 MHz, DMSO-*d_6_*) δ 13.06 (s, 1H), 10.18 (s, 1H), 7.95 (dd, *J* = 7.5, 1.3 Hz, 1H), 7.50–7.38 (m, 2H), 7.33–7.25 (m, 3H), 7.05 (d, *J* = 8.3 Hz, 2H), 5.45 (d, *J* = 1.3 Hz, 1H), 4.27 (d, *J* = 1.3 Hz, 1H), 1.21 (s, 9H). ^13^C-NMR (101 MHz, DMSO-*d_6_*) δ 172.4, 160.7, 150.4, 136.2, 133.5, 132.2, 130.4, 129.1, 128.3, 127.0, 126.3, 125.8, 65.4, 51.8, 34.6, 31.5. HRMS (ESI), *m*/*z* calcd for C_20_H_22_NO_4_ [M + H]^+^ 340.1543, found 340.1553.

#### 3.2.5. 2-(2-Oxo-2H-chromen-3-yl)benzoic Acid **8**

Homophthalic acid (180 mg, 1 mmol) and 2-hydroxybenzaldehyde (122 mg, 1 mmol) were heated at reflux toluene (15 mL) in a flask equipped with a Dean-stark distilling trap for 24 h. On cooling to r. t., the precipitate formed was separated by filtration, washed with hexane (5 mL), air-dried and crystallized from MeOH: H_2_O (4:2) to give 194 mg, 0.73 mmol (73%) of the title compound as white solid: mp 259.1−259 °C; ^1^H-NMR (400 MHz, DMSO-*d*_6_) δ 12.85 (br.s, 1H, COOH), 8.00 (s, 1H, CH(Ar)), 7.95 (d, *J* = 7.7 Hz, 1H, CH(Ar)), 7.79–7.73 (m, 1H, CH(Ar)), 7.69 (td, *J* = 7.5, 1.2 Hz, 1H, CH(Ar)), 7.65–7.60 (m, 1H, CH(Ar)), 7.57 (t, *J* = 7.6 Hz, 1H, CH(Ar)), 7.50 (d, *J* = 7.5 Hz, 1H, CH(Ar)), 7.45 (d, *J* = 8.2 Hz, 1H, CH(Ar)), 7.39 (t, *J* = 7.9 Hz, 1H, CH(Ar)). ^13^C-NMR (101 MHz, DMSO-*d*_6_) δ 168.2, 160.3, 153.4, 139.0, 136.4, 132.6, 131.9, 131.8, 131.4, 130.7, 130.2, 129.3, 128.8, 125.1, 119.9, 116.4. HRMS (ESI), *m*/*z* calcd for C_16_H_10_NaO_4_ [M + Na]^+^ 289.0471, found 289.0461.

#### 3.2.6. General Procedure for the Preparation of *N*-Benzyloxy THIQ Acids **9a**–**g**

A mixture of homophthalic acid (3.6 g, 20 mmol), *O*-benzylhydroxylamine (2.5 g, 20 mmol) and the respective aldehyde (20 mmol) in toluene (20 mL) was placed in a pre-heated oil bath and refluxed for 48 h in a flask equipped a Dean-Stark distilling trap. A thick precipitate which formed on cooling the reaction mixture to −20 °C was isolated by filtration, washed with hexane, and crystallized from aqueous methanol to provide pure title compounds.

*(3S/R,4S/R)-2-(Benzyloxy)-3-(4-fluorophenyl)-1-oxo-1,2,3,4-tetrahydroisoquinoline-4-carboxylic acid* (**9a**). Yield 5.7 g, 73%. White solid, mp 138–140 °C; ^1^H-NMR (400 MHz, DMSO-*d*_6_) δ 13.21 (s, 1H), 7.98 (dd, *J* = 7.6, 1.5 Hz, 1H), 7.51 (td, *J* = 7.5, 1.6 Hz, 1H), 7.45 (ddd, *J* = 6.7, 4.8, 1.6 Hz, 3H), 7.41–7.30 (m, 4H), 7.24 (td, *J* = 8.5, 6.3 Hz, 2H), 7.10 (t, *J* = 8.8 Hz, 2H), 5.78 (d, *J* = 2.0 Hz, 1H), 5.07 (d, *J* = 9.9 Hz, 1H), 5.00 (d, *J* = 10.0 Hz, 1H), 4.42 (d, *J* = 2.1 Hz, 1H). ^13^C-NMR (101 MHz, DMSO-*d*_6_) δ 171.5, 161.5 (d, *J* = 243.9 Hz), 160.9, 135.0, 134.6 (d, *J* = 3.0 Hz), 133.2, 132.5, 129.9, 129.2, 128.5, 128.3, 128.2 (d, *J* = 8.2 Hz), 128.1, 128.0, 126.9, 115.4 (d, *J* = 21.6 Hz), 75.9, 62.7, 51.4. HRMS (ESI), *m*/*z* calcd for C_23_H_18_FNO_4_Na [M + Na]^+^ 414.1112, found 414.1113.

*(3S/R,4S/R)-2-(Benzyloxy)-3-(4-methoxyphenyl)-1-oxo-1,2,3,4-tetrahydroisoquinoline-4-carboxylic acid* (**9b**). Yield 5.8 g, 54%. White solid, mp 176–178 °C; ^1^H-NMR (400 MHz, DMSO-*d*_6_) δ 13.15 (s, 1H), 7.96 (dd, *J* = 7.7, 1.5 Hz, 1H), 7.50 (dd, *J* = 7.4, 1.6 Hz, 1H), 7.44 (ddd, *J* = 7.1, 5.2, 1.7 Hz, 3H), 7.40–7.30 (m, 4H), 7.12–7.03 (m, 2H), 6.89–6.73 (m, 2H), 5.68 (d, *J* = 2.1 Hz, 1H), 5.04 (d, *J* = 9.9 Hz, 1H), 4.98 (d, *J* = 10.0 Hz, 1H), 4.36 (d, *J* = 2.1 Hz, 1H), 3.65 (s, 3H). ^13^C-NMR (101 MHz, DMSO-*d*_6_) δ 171.7, 161.0, 158.7, 135.1, 133.5, 132.4, 130.2, 129.9, 129.2, 128.5, 128.3, 128.2, 128.0, 127.3, 126.9, 113.9, 75.8, 63.0, 55.0, 51.6. HRMS (ESI), *m*/*z* calcd for C_24_H_21_NO_5_Na [M + Na]^+^ 426.1312, found 426.1318.

*(3S/R,4S/R)-2-(Benzyloxy)-3-(4-(tert-butyl)phenyl)-1-oxo-1,2,3,4-tetrahydroisoquinoline-4-carboxylic acid* (**9c**). Yield 6.0 g, 70%. White solid, mp 190–191 °C; ^1^H-NMR (400 MHz, DMSO-*d*_6_) δ 13.19 (s, 1H), 7.99 (dd, *J* = 7.7, 1.5 Hz, 1H), 7.50 (td, *J* = 7.5, 1.6 Hz, 1H), 7.44 (ddd, *J* = 7.5, 5.7, 1.6 Hz, 3H), 7.39–7.31 (m, 4H), 7.28 (d, *J* = 8.5 Hz, 2H), 7.11 (d, *J* = 8.5 Hz, 2H), 5.72 (d, *J* = 2.1 Hz, 1H), 5.05 (d, *J* = 9.9 Hz, 1H), 5.01 (d, *J* = 9.9 Hz, 1H), 4.42 (d, *J* = 2.1 Hz, 1H), 1.18 (s, 9H). ^13^C-NMR (101 MHz, DMSO-*d*_6_) δ 171.7, 161.1, 150.1, 135.4, 135.0, 133.4, 132.4, 130.0, 129.3, 128.5, 128.3, 128.2, 128.0, 126.9, 125.9, 125.4, 75.9, 63.2, 51.5, 34.1, 30.9. HRMS (ESI), *m*/*z* calcd for C_27_H_27_NO_4_Na [M + Na]^+^ 452.1832, found 452.1836.

*(3S/R,4S/R)-2-(Benzyloxy)-3-(4-(methoxycarbonyl)phenyl)-1-oxo-1,2,3,4-tetrahydroisoquinoline-4-carboxylic acid* (**9d**). Yield 5.6 g, 65%. White solid, mp 197–199 °C; ^1^H-NMR (400 MHz, DMSO-*d*_6_) δ 13.26 (s, 1H), 7.98 (dd, *J* = 7.6, 1.6 Hz, 1H), 7.92–7.77 (m, 2H), 7.49 (td, *J* = 7.5, 1.6 Hz, 1H), 7.46–7.40 (m, 3H), 7.39–7.27 (m, 6H), 5.88 (d, *J* = 1.9 Hz, 1H), 5.09 (d, *J* = 10.0 Hz, 1H), 5.03 (d, *J* = 10.0 Hz, 1H), 4.48 (d, *J* = 2.0 Hz, 1H), 3.78 (s, 3H). ^13^C-NMR (101 MHz, DMSO-*d*_6_) δ 171.4, 165.7, 161.0, 143.8, 135.0, 133.0, 132.5, 129.9, 129.4, 129.2, 129.1, 128.5, 128.3, 128.1, 128.0, 127.0, 126.5, 76.0, 63.2, 52.1, 51.3. HRMS (ESI), *m*/*z* calcd for C_25_H_21_NO_6_Na [M + Na]^+^ 454.1261, found 454.1264.

*(3S/R,4S/R)-2-(Benzyloxy)-3-(4-bromophenyl)-1-oxo-1,2,3,4-tetrahydroisoquinoline-4-carboxylic acid* (**9e**). Yield 5.1 g, 57%. White solid, mp 187–189 °C; ^1^H-NMR (400 MHz, DMSO-*d*_6_) δ 13.16 (s, 1H), 7.97 (dd, *J* = 7.6, 1.5 Hz, 1H), 7.58–7.40 (m, 6H), 7.40–7.26 (m, 4H), 7.24–7.08 (m, 2H), 5.76 (d, *J* = 2.0 Hz, 1H), 5.07 (d, *J* = 9.9 Hz, 1H), 5.00 (d, *J* = 10.0 Hz, 1H), 4.42 (d, *J* = 2.0 Hz, 1H). ^13^C-NMR (101 MHz, DMSO-*d*_6_) δ 171.4, 161.0, 137.9, 135.0, 133.1, 132.5, 131.5, 129.9, 129.2, 128.5, 128.4, 128.3, 128.1, 128.0, 127.0, 120.9, 75.9, 62.9, 51.3. HRMS (ESI), *m*/*z* calcd for C_23_H_18_BrNO_4_Na [M + Na]^+^ 474.0311, found 4754.0305.

*(3S/R,4S/R)-2-(Benzyloxy)-3-(2-methylphenyl)-1-oxo-1,2,3,4-tetrahydroisoquinoline-4-carboxylic acid* (**9f**). Yield 4.2 g, 54%. White solid, mp 176–178 °C; ^1^H-NMR (400 MHz, DMSO-*d*_6_) δ 13.25 (s, 1H), 8.03 (dd, *J* = 7.3, 1.9 Hz, 1H), 7.54–7.43 (m, 1H), 7.43–7.33 (m, 5H), 7.30 (dd, *J* = 6.9, 1.9 Hz, 1H), 7.22 (d, *J* = 7.5 Hz, 1H), 7.12 (td, *J* = 7.4, 1.3 Hz, 1H), 6.98 (td, *J* = 7.6, 1.4 Hz, 1H), 6.68 (dd, *J* = 7.9, 1.2 Hz, 1H), 5.85 (d, *J* = 1.8 Hz, 1H), 5.01 (d, *J* = 10.1 Hz, 1H), 4.98 (d, *J* = 10.1 Hz, 1H), 4.25 (d, *J* = 1.8 Hz, 1H), 2.44 (s, 3H). ^13^C-NMR (101 MHz, DMSO-*d*_6_) δ 171.8, 161.3, 135.8, 135.2, 135.0, 132.9, 132.3, 131.1, 130.1, 129.2, 128.5, 128.3, 128.2, 128.0, 127.6, 126.8, 125.8, 124.7, 76.0, 61.1, 49.9, 18.5. HRMS (ESI), *m*/*z* calcd for C_24_H_21_NO_4_Na [M + Na]^+^ 410.1363, found 410.1371.

*(3S/R,4S/R)-2-(Benzyloxy)-3-(4-nitrophenyl)-1-oxo-1,2,3,4-tetrahydroisoquinoline-4-carboxylic acid* (**9g**). Yield 3.3 g, 47%. Pale yellow solid, mp 190–191 °C; ^1^H-NMR (400 MHz, DMSO-*d*_6_) δ 13.30 (s, 1H), 8.13 (d, *J* = 8.8 Hz, 2H), 7.99 (dd, *J* = 7.6, 1.6 Hz, 1H), 7.58–7.41 (m, 6H), 7.40–7.30 (m, 4H), 5.97 (d, *J* = 1.8 Hz, 1H), 5.10 (d, *J* = 10.0 Hz, 1H), 5.04 (d, *J* = 10.0 Hz, 1H), 4.53 (d, *J* = 2.0 Hz, 1H). ^13^C-NMR (101 MHz, DMSO-*d*_6_) δ 171.2, 161.0, 147.0, 146.0, 134.9, 132.9, 132.6, 130.0, 129.3, 128.6, 128.3, 128.2, 127.8, 127.5, 127.0, 123.7, 76.0, 62.9, 51.0. HRMS (ESI), *m*/*z* calcd for C_23_H_18_N_2_O_6_Na [M + Na]^+^ 441.1057, found 441.1056.

#### 3.2.7. 2-(Benzyloxy)-3-(4-methoxyphenyl)-3,4-dihydroisoquinolin-1(2*H*)-one (**10**)

To a stirred solution of compound **9a** (403 mg, 1 mmol) in DMSO (3 mL) in a screw-cap vial K_2_CO_3_ (207 mg, 1.5 mmol) was added. The mixture was heated to 110 °C and stirred overnight. After cooling to room temperature, the reaction mixture was diluted with EtOAc (30 mL) and water (15 mL). The organic layer was then separated and washed with water (3 × 15 mL), dried over Na_2_SO_4_, filtered, and concentrated in vacuo to give pure title compound as beige foam. Yield 275 mg, 77%. ^1^H-NMR (400 MHz, CDCl_3_) δ 8.19 (dd, *J* = 7.6, 1.6 Hz, 1H), 7.44–7.35 (m, 2H), 7.33 (s, 5H), 7.17–7.10 (m, 2H), 7.08–7.03 (m, 1H), 6.85–6.72 (m, 2H), 5.15 (d, *J* = 10.3 Hz, 1H), 4.91 (d, *J* = 10.3 Hz, 1H), 4.74 (t, *J* = 5.6 Hz, 1H), 3.75 (s, 3H), 3.41 (dd, *J* = 16.1, 6.1 Hz, 1H), 3.16 (dd, *J* = 16.1, 5.2 Hz, 1H). ^13^C-NMR (101 MHz, CDCl_3_) δ 164.3, 159.4, 135.6 (d, *J* = 4.9 Hz), 132.6, 131.1, 129.8, 128.8, 128.8, 128.5, 128.2, 128.1, 127.6, 127.3, 114.1, 63.4, 55.4, 36.9. HRMS (ESI), *m*/*z* calcd for C_23_H_21_NO_3_Na [M + Na]^+^ 382.1414, found 382.1418.

#### 3.2.8. General Procedure for *O*-debenzylation

##### Synthesis of Compounds **7a**, **11**, and **12**

Compound **7a** (100 mg, 0.25 mmol), **10** (50 mg, 0.14 mmol) or **7c** (150 mg, 0.38 mmol) was dissolved in MeOH (15 mL), 0.05 molar equiv. of 10% Pd/C was added, and the reaction was stirred under the atmosphere of hydrogen (introduced via a balloon) for 16 h at room temperature. The catalyst was removed by filtration of the reaction mixture through a pad of Celite and the filtrate was concentrated in vacuo to give pure title compounds.

*2-Hydroxy-3-(4-methoxyphenyl)-3,4-dihydroisoquinolin-1(2H)-one* (**11**). Yield 35 mg, 94%. White solid, mp 95–96 °C (MeOH); ^1^H-NMR (400 MHz, DMSO-*d*_6_) δ 9.92 (s, 1H), 7.93 (dd, *J* = 7.6, 1.5 Hz, 1H), 7.42 (td, *J* = 7.4, 1.5 Hz, 1H), 7.35 (t, *J* = 7.4 Hz, 1H), 7.20 (d, *J* = 7.4 Hz, 1H), 7.09 (d, *J* = 8.7 Hz, 2H), 6.82 (d, *J* = 8.7 Hz, 2H), 5.07 (dd, *J* = 6.4, 3.8 Hz, 1H), 3.77–3.48 (m, 4H), 3.21–3.09 (m, 1H). ^13^C-NMR (101 MHz, DMSO-*d*_6_) δ 161.4, 158.5, 135.4, 131.9, 131.8, 128.6, 127.8, 127.4, 126.8, 126.7, 113.7, 62.5, 55.0, 36.0. HRMS (ESI), *m*/*z* calcd for C_32_H_30_N_2_O_6_Na [2M + Na]^+^ 561.1996, found 561.1994.

*(3R/S,4R/S)3-(2-Hydroxyphenyl)-1-oxo-1,2,3,4-tetrahydroisoquinoline-4-carboxylic acid* (**12**). Yield 100 mg, 93%. White solid, mp 202.3−202.8 °C; ^1^H-NMR (400 MHz, DMSO-*d*_6_) δ 12.79 (s, 1H, COOH), 9.85 (s, 1H, OH), 8.24 (d, *J* = 4.9 Hz, 1H, NH), 7.91 (dd, *J* = 7.2, 1.9 Hz, 1H, CH(Ar)), 7.39 (td, *J* = 7.1, 1.7 Hz, 2H, 2CH(Ar)), 7.22 (d, *J* = 7.0, 1H, CH(Ar)), 7.00 (t, *J* = 8.4 Hz, 1H, CH(Ar)). 6.81 (d, *J* = 8.1 Hz, 1H, CH(Ar)), 6.71 (d, *J* = 7.5 Hz, 1H, CH(Ar)), 6.59 (t, *J* = 7.5 Hz, 1H, CH(Ar)), 5.39 (dd, *J* = 5.0, 1.7 Hz, 1H, 3-H), 4.07 (s, 1H, 4-H). ^13^C-NMR (101 MHz, DMSO-*d*_6_) δ 173.0, 164.8, 154.4, 135.4, 132.2, 130.1, 129.4, 128.5, 128.0, 127.7, 126.9, 126.9, 119.0, 115.6, 50.8, 48.6. HRMS (ESI), *m*/*z* calcd for C_16_H_14_NO4 [M + H]^+^ 284.0917, found 284.0924.

### 3.3. Spectrophotometric Investigation of Compounds *7d*, *7f*, *7g*, *7h*, *7j*, and Their Complexation with Iron(III) Nitrate

Spectrophotometric measurements were performed on a UV-1800 Shimadzu double beam spectrophotometer (Kyoto, Japan) using 10.00 mm quartz cells. Methanol or methanol—water (85:15) mixture were used as solvent. All measurements were performed at room temperature (26 °C) in stoppered cells. For experiments with Fe^3+^ complexation, Fe(NO_3_)_3_∙9H_2_O was used as iron source.

UV-Vis spectra of compounds **7d**, **7f**, **7g**, **7h**, **7j** (Figure 2a and ESI, Figures S1–S3) were recorded for MeOH solutions (C = 5 × 10^−5^ M) prior to titration experiments. Sample preparation for UV-Vis titrations (Figure 3a and ESI, Figures S4–S8): a 1500 μL aliquote of 0.001 M stock solution of compound **7d**, **7f**, **7g**, **7h**, **7j** (1.5 × 10^−6^ mol) in MeOH was placed in a 10.00 mm quartz cuvette equipped with magnetic stir bar and diluted to 3 mL with 1050 μL of MeOH and 450 μL of water to obtain solution in 85% aq. MeOH (C_L_ = 5 × 10^−4^ M). Aliquotes, 5 or 10 μL of 0.015 M aqueous Fe(NO_3_)_3_ solution, were added to the cell with calibrated micropipette in a stepwise manner (C_M_ = 0.25−5 × 10^−4^ M). The solution was vigorously stirred after addition of each aliquote followed by registration of absorbance spectrum over the wavelength range of 300–700 nm vs. 85% aq. MeOH (all measurements were performed in stoppered cuvettes). Color of the solution was changed from colorless to purple, which corresponds to appearance of a new absorption band with maximum at 500 nm. The absorbance at selected wavelength was plotted as a function of [Fe^3+^]/[ligand] ratio to give binding isotherms presented on Figure 3b and ESI, Figures S4–S8. The maxima of these curves correspond to the maximum formation of complexes and indicate to the stoichiometry of the complexes. The average stoichiometry of complex is estimated from the point where this curve changes its slope (this point is the intersect point of bilinear fitting of experimental curve). 1:2 metal-to-ligand ratio was observed in all cases. Formation constants (Table 1) were calculated from curves on Figure 3a using following equations:

(1) $$K=\frac{\left[ML_{x}\right]}{\left[M\right]{\left[L\right]}^{x}}$$

(2) $$K_{1}K_{2}{\left[L\right]}^{3}+K_{1}\left(1+K_{2}\left(2C_{M}-C_{L}\right)\right){\left[L\right]}^{2}+\left(1+K_{1}\left(C_{M}-C_{L}\right)\right)\left[L\right]=0$$

(3) $$\Delta A_{obs}=\frac{\varepsilon_{\Delta ML}\left(C_{M}\right)K_{1}\left[L\right]+2\varepsilon_{\Delta ML2}\left(C_{M}\right)K_{1}K_{2}{\left[L\right]}^{2}}{1+K_{1}\left[L\right]+K_{1}K_{2}{\left[L\right]}^{2}}$$

Experimental curves were fitted to equation 3 corresponding to 1:2 metal-to-ligand complex formation using nonlinear curve-fitting performed in ThordarsonFittingProgram [25]. The program is based on the iterative adjustment of calculated values of absorbance (A) to observed values using Equation (3) previously derived [25] from Equations (1) and (2), where *K*_1_ and *K*_2_ are stepwise formation constants; ε*_Δ_*_ML_ = ε_ML_ − ε_L_ and ε*_Δ_*_ML2_ = ε_ML2_ − ε_ML,_ where ε*_i_* are molar absorptivities of corresponding species; C_L_ and C_M_ are analytical concentrations of ligand and Fe^3+^ respectively and L is free ligand concentration.

## 4. Conclusions

In summary, we have developed a practically convenient, three-component approach to *N*-hydroxytetrahydroisoquinoline (THIQ) acids via a variant of the Castagnoli–Cushman reaction involving in situ cyclodehydration of homophthalic acid with concomitant formation of an oxime in refluxing toluene. Using hydroxylamine acetate or *O*-benzylhydroxylamine in lieu of the hydroxylamine hydrochloride typically employed to prepare oximes was key to the success of the reaction. For prepared *N*-bezyloxy THIQ acids decarboxylation and debenzylation reactions were additionally investigated. Five selected cyclic hydroxamic acid compounds produced in the course of this study have been profiled and confirmed as ligands for Fe^3+^. Thus, a new, practically simple and flexible approach to potential iron overload disease treatments or analogs of bacterial siderophores for antibiotic delivery has been developed.

## Figures and Tables

**Table 1:** Stoichiometry and K*f* values for Fe^3+^-**7d,** Fe^3+^-**7f,** Fe^3+^-**7g,** Fe^3+^-**7h** and Fe^3+^-**7j** complexes determined by the mole-ratio method.

Ligand	M:L	K_1,2_	log K_1,2_	λ [nm] ^a^
**7d**	1:1	1.67 × 10^6^	6.22	500
	1:2	2.32 × 10^5^	5.36	
**7f**	1:1	5.09 × 10^6^	6.71	500
	1:2	6.04 × 10^5^	5.78	
**7g**	1:1	2.16 × 10^7^	7.33	500
	1:2	5.38 × 10^4^	4.73	
**7h**	1:1	8.22 × 10^5^	5.91	500, 550, 600
	1:2	3.39 × 10^5^	5.53	
**7j**	1:1	2.31 × 10^5^	5.36	470
	1:2	4.06 × 10^5^	5.61	

^a^ Characteristic absorbance wavelength at which absorbance of the complex was measured.

## Supplementary Materials

The following are available online at https://www.mdpi.com/1420-3049/24/5/864/s1, copies of ^1^H and ^13^C spectra for all new compounds **7c–j**,**l**,**m**, **9a**–**g**, **10**-**12**, UV-Vis spectra for compounds **7d**,**f**–**h**,**j** (Figures S1–S3), results of UV-Vis titration of compounds **7d**,**f**–**h**,**j** with FeCl_3_ (Figure S4–S8).
