# Supplementary material for: Facile Access to Fe(III)-Complexing Cyclic Hydroxamic Acids in a Three-Component Format
Source: Molecules. 2019 Feb 28;24(5):864. doi: 10.3390/molecules24050864 (PMC6429155; doi:10.3390/molecules24050864)
Supplement: Supplementary file 1 [file molecules-24-00864-s001.pdf]

Supporting Information for

Facile access to Fe(III)-complexing cyclic hydroxamic acids in a three-component format

Evgeny Chupakhin[a,b], Olga Bakulina[a], Dmitry Dar'in[a] and Mikhail Krasavin[a,b]*

[a] *Saint Petersburg State University, Saint Petersburg, 199034 Russian Federation*

[b] *Immanuel Kant Baltic Federal University, Kaliningrad, 236016 Russian Federation*

* Corresponding author at: Laboratory of Chemical Pharmacology, Institute of Chemistry, Saint Petersburg State University, 26 Universitetskyi prospekt, Peterhof 198504 Russian Federation

E-mail address: m.krasavin@spbu.ru (M. Krasavin)

Contents

Copies of ^{1}H and ^{13}C NMR spectra for compounds **7c-j,l,m, 8, 9a-g and 10-12** S2-21

Electronic absorption spectra of free **7d,f,g,h,j** ligands (Figures S1-S3) S22

Electronic absorption spectra of ligands **7** in the presence of increasing concentration of Fe^{3+} and corresponding binding isotherms (Figures S4-S8) S23-24

^{1}H and ^{13}C NMR of compound **7c**

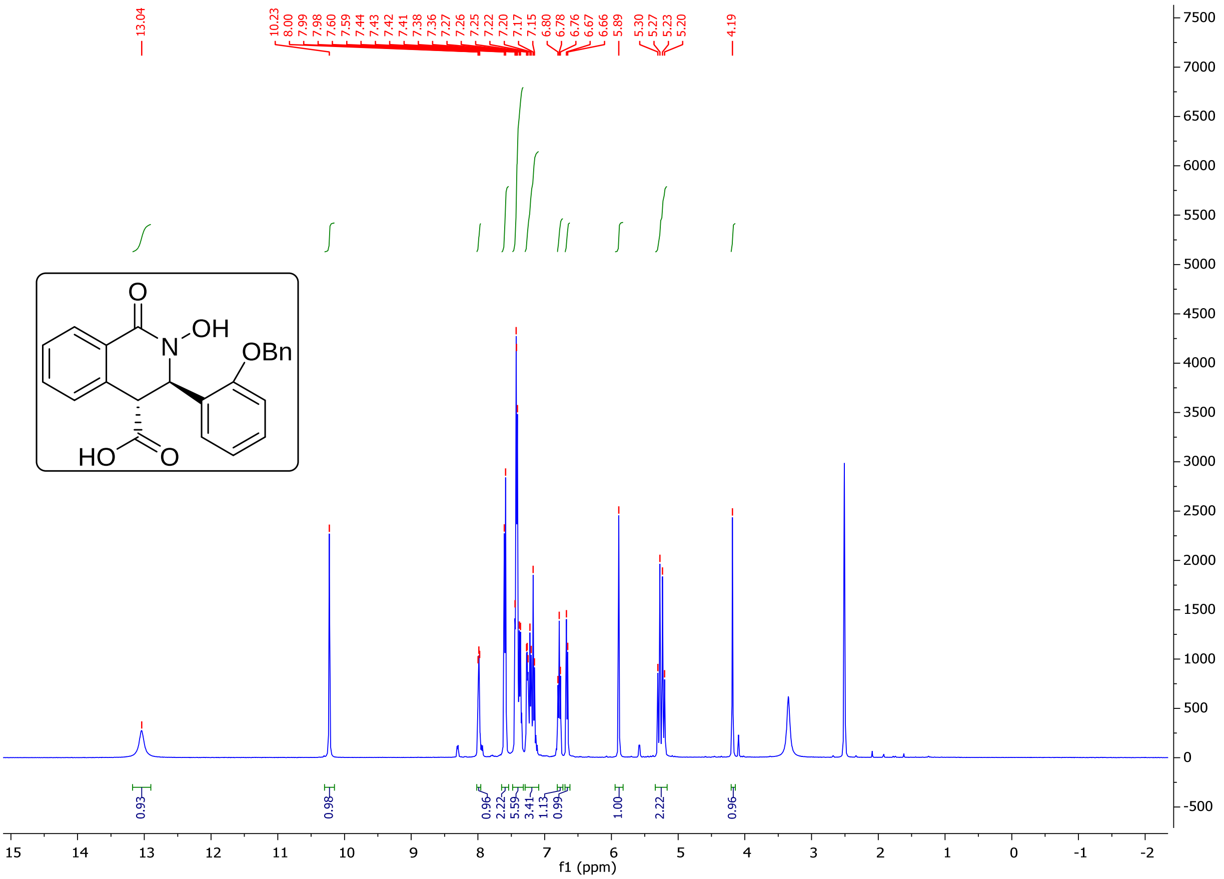

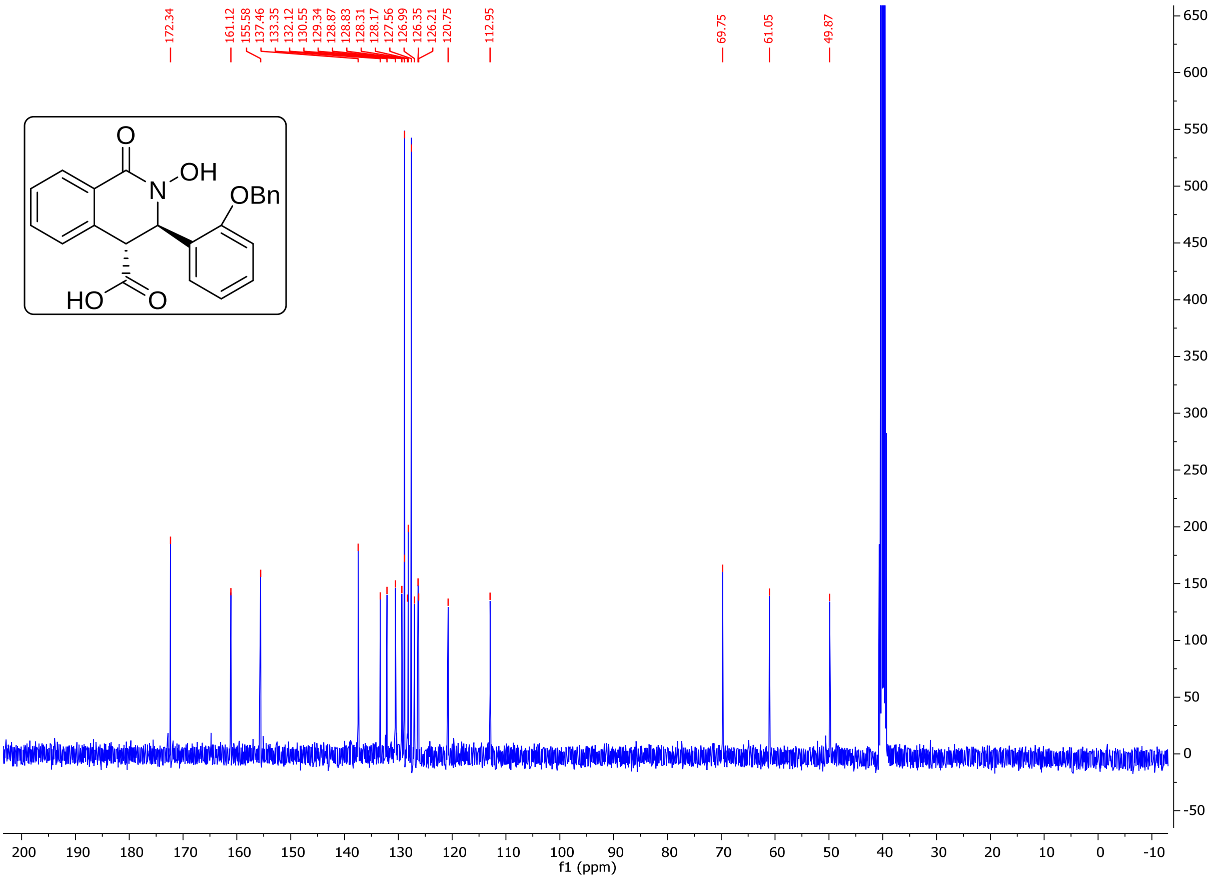

^{1}H and ^{13}C NMR of compound **7d**

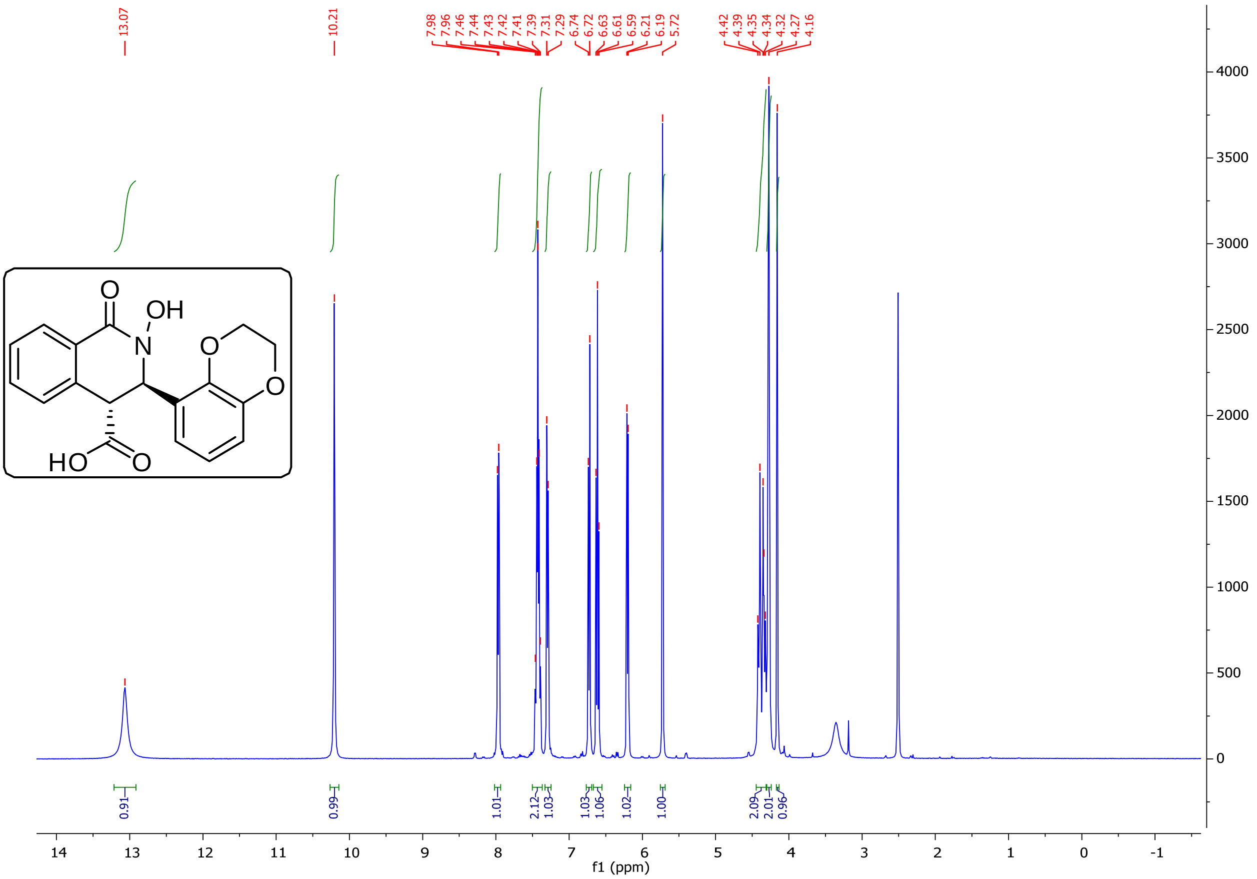

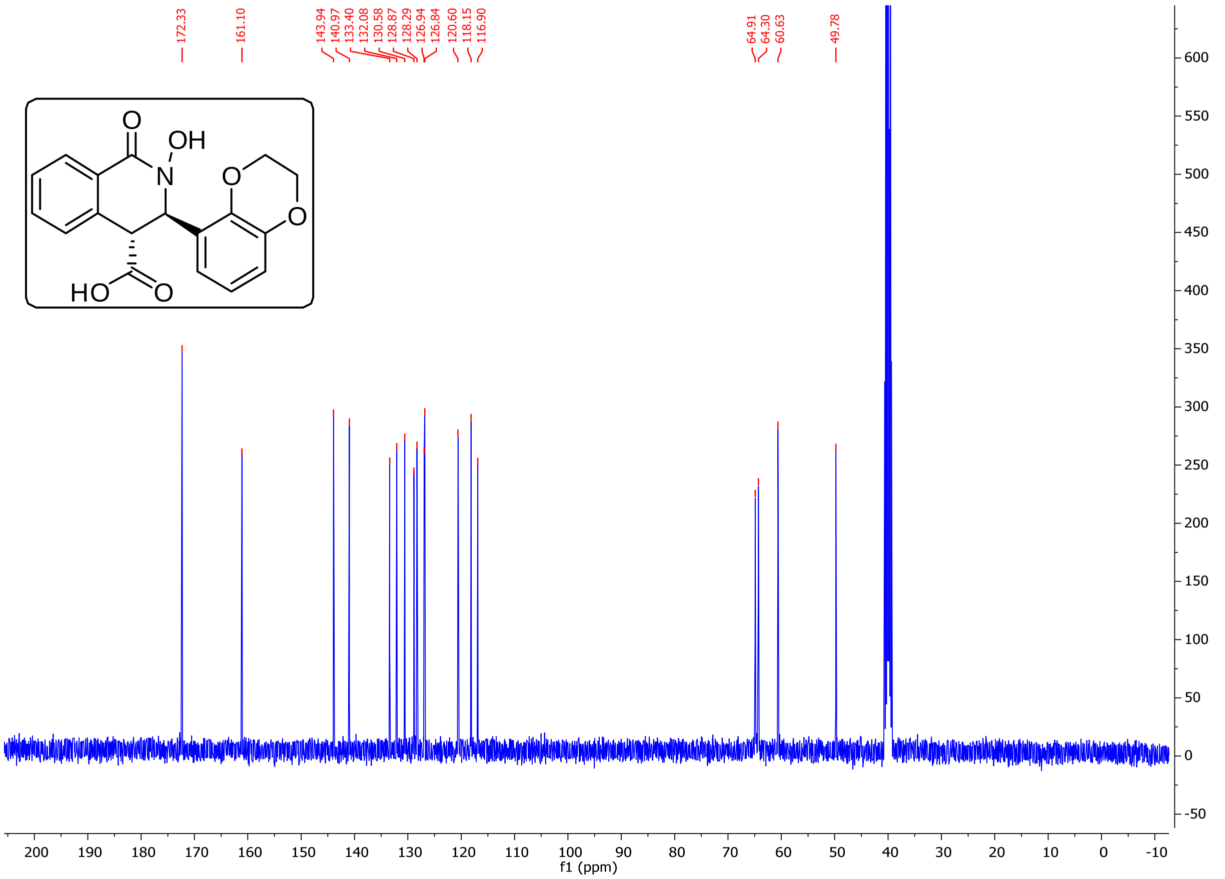

^{1}H and ^{13}C NMR of compound **7e**

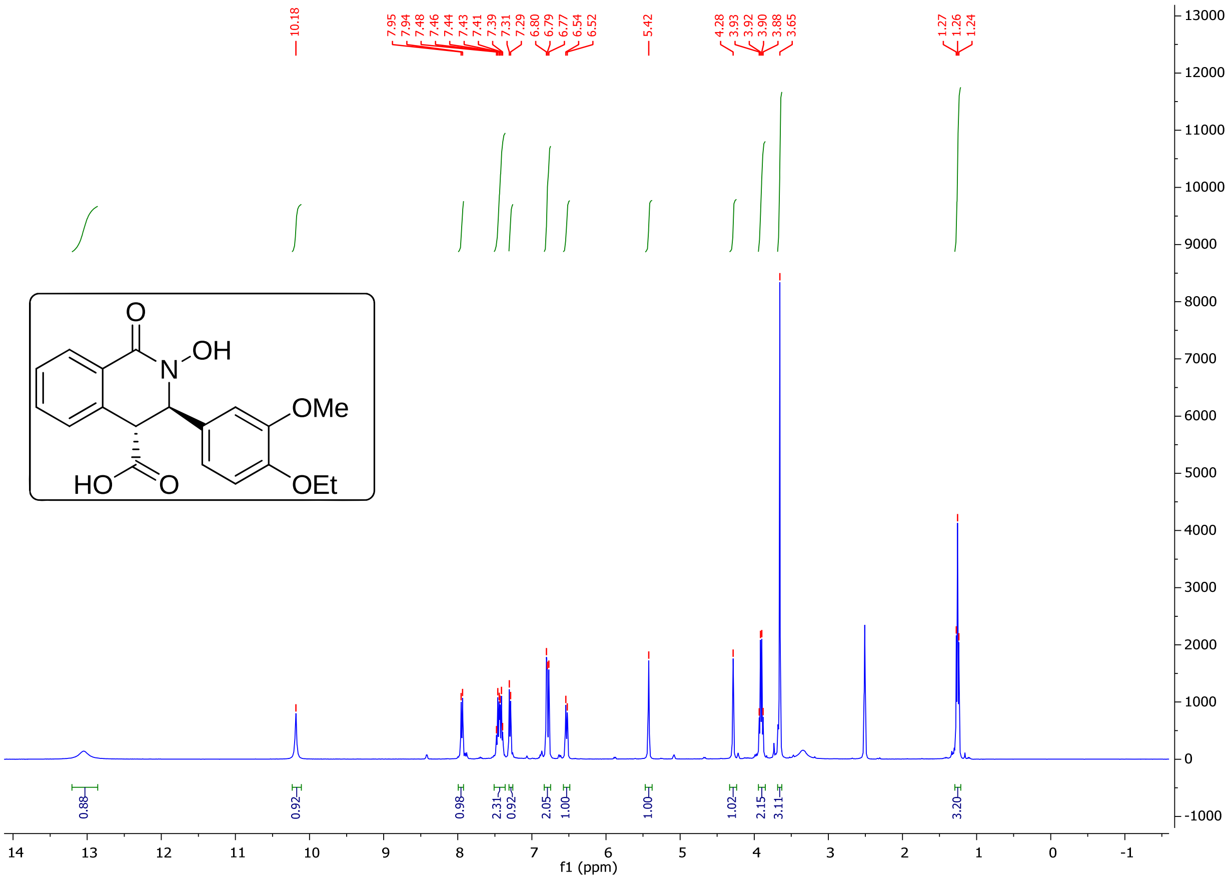

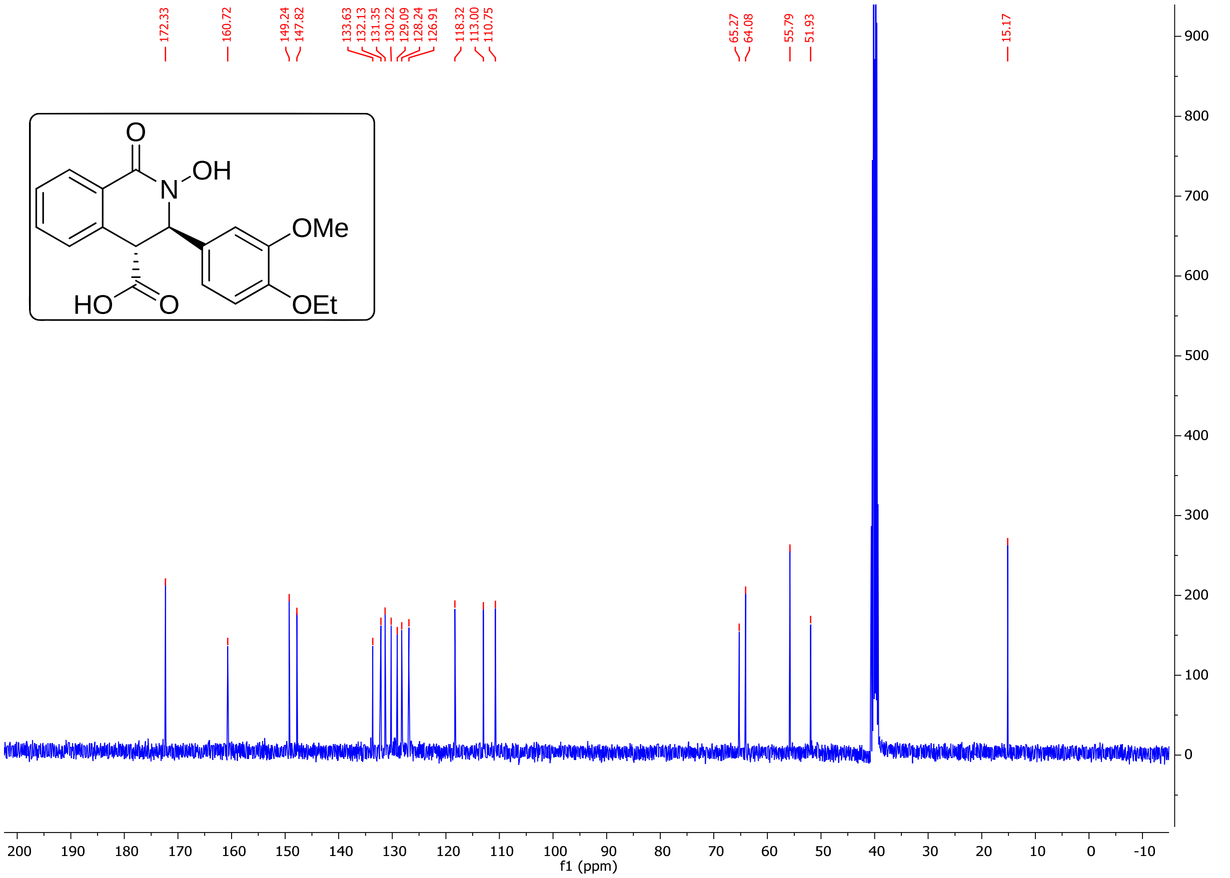

^{1}H and ^{13}C NMR of compound **7f**

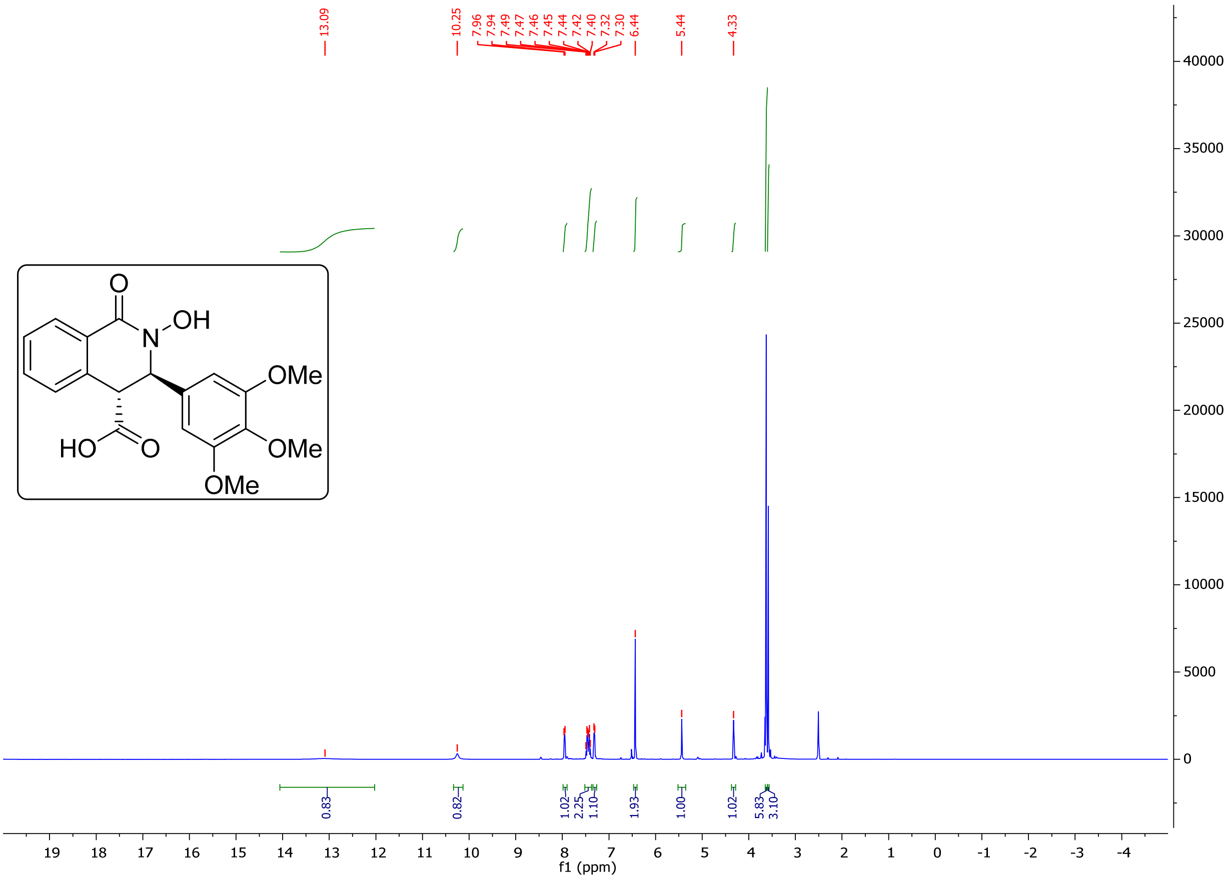

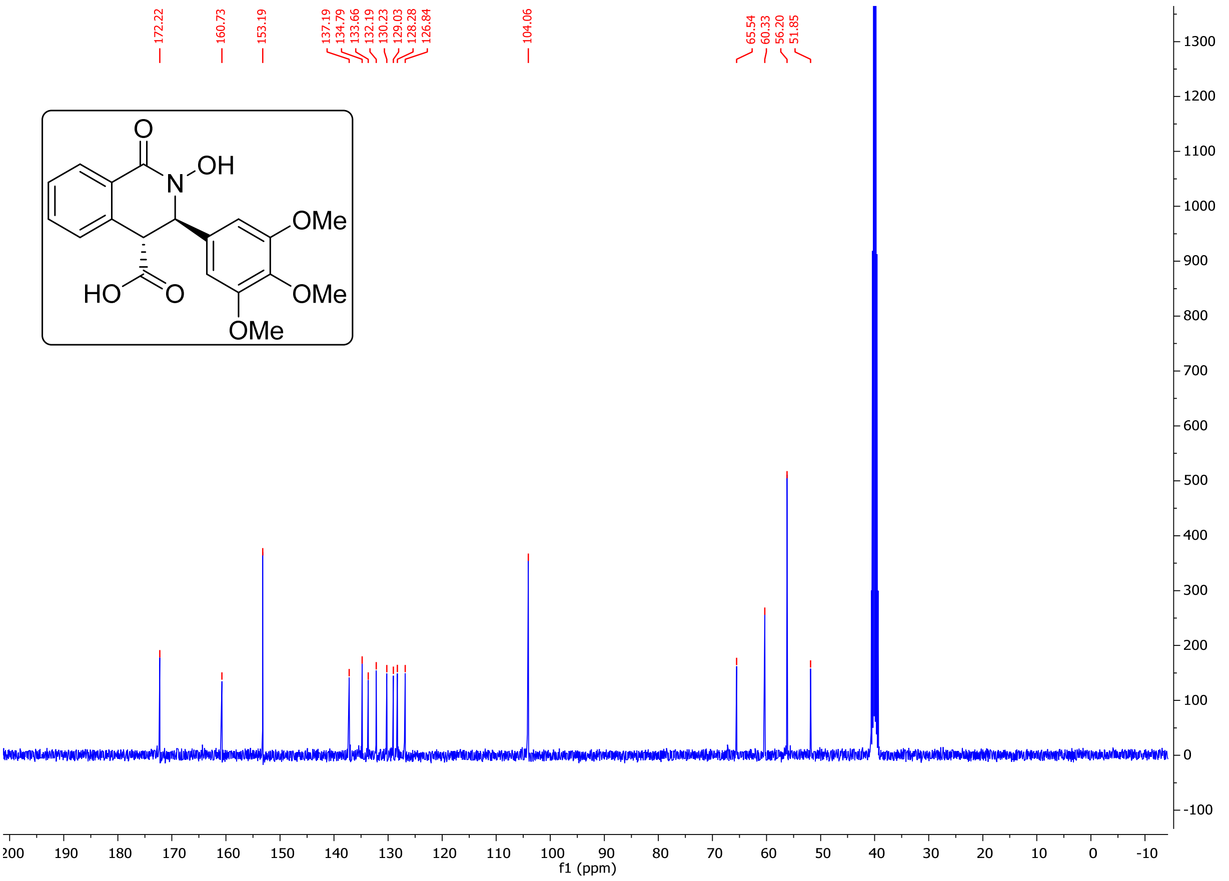

^{1}H and ^{13}C NMR of compound **7g**

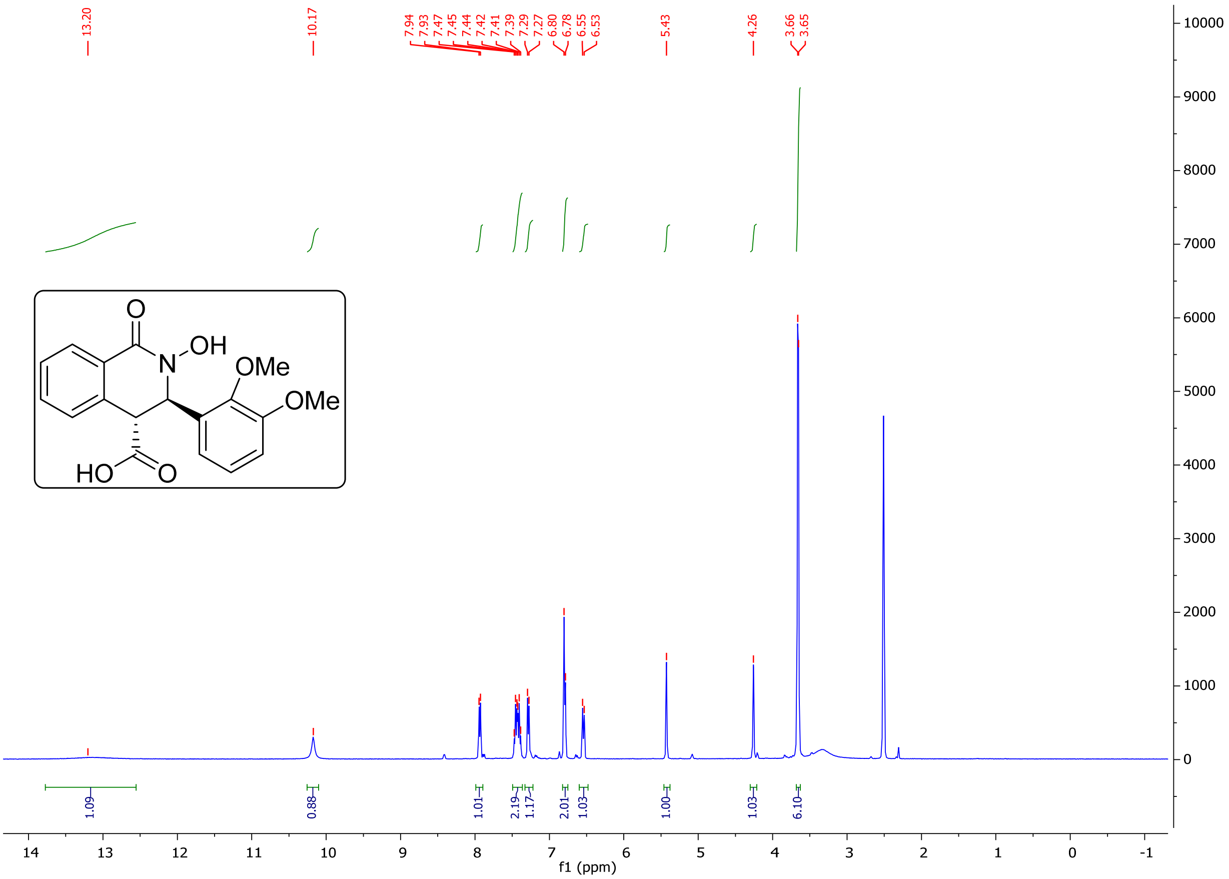

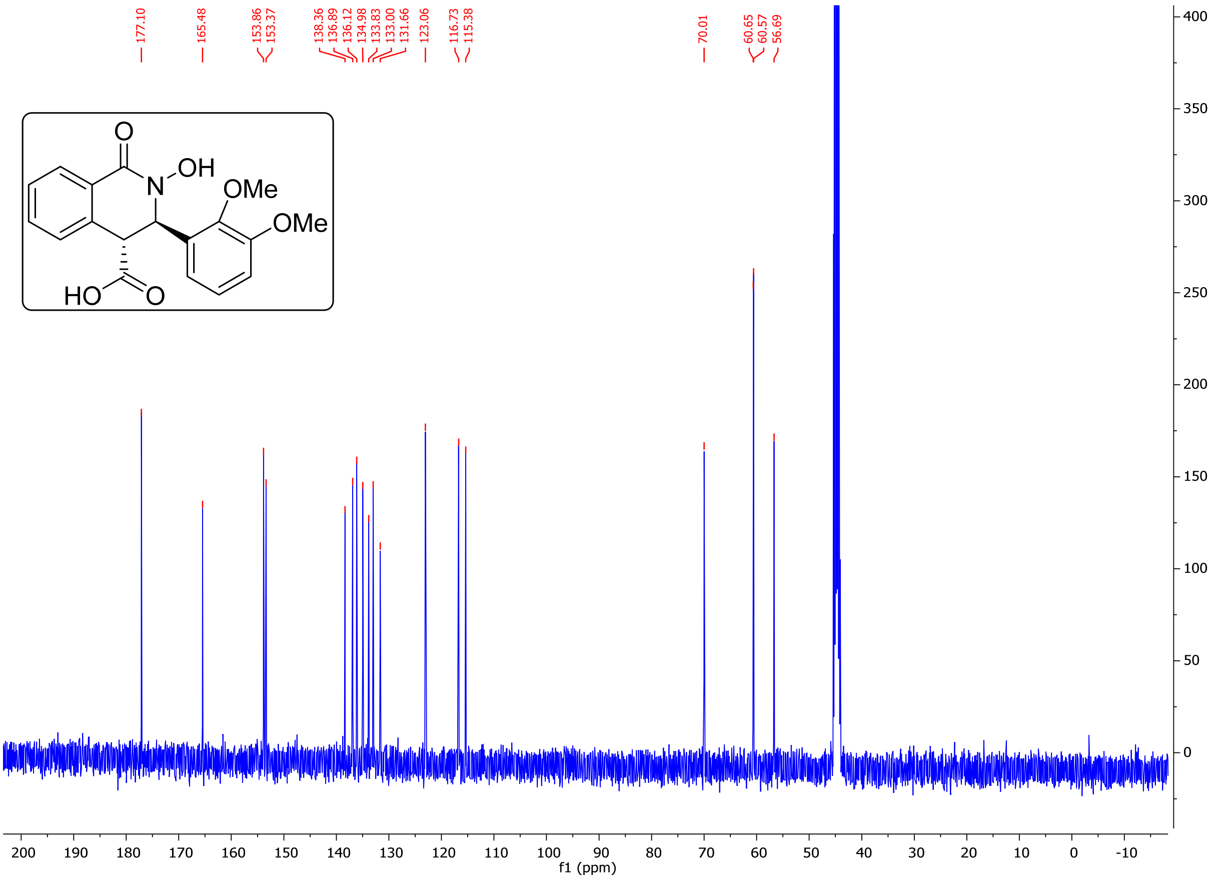

^{1}H and ^{13}C NMR of compound **7h**

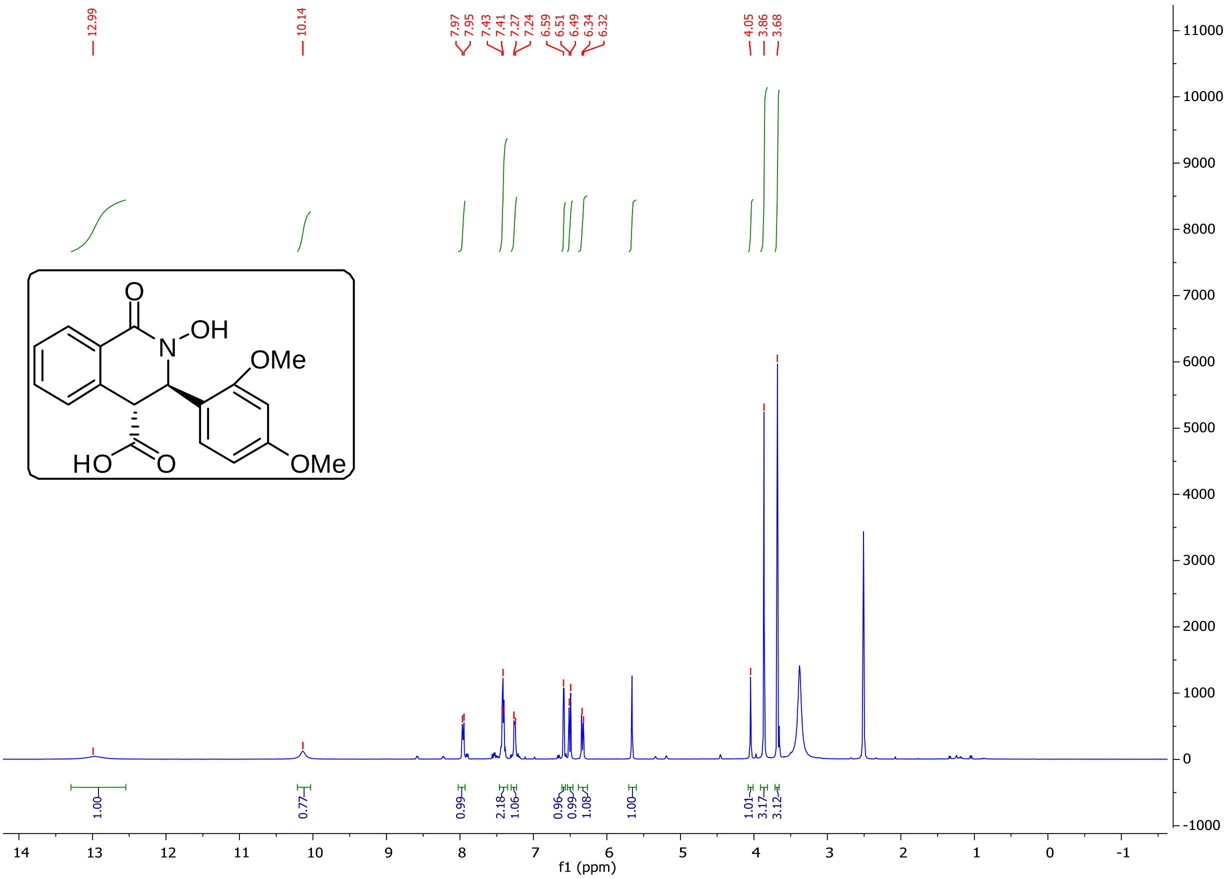

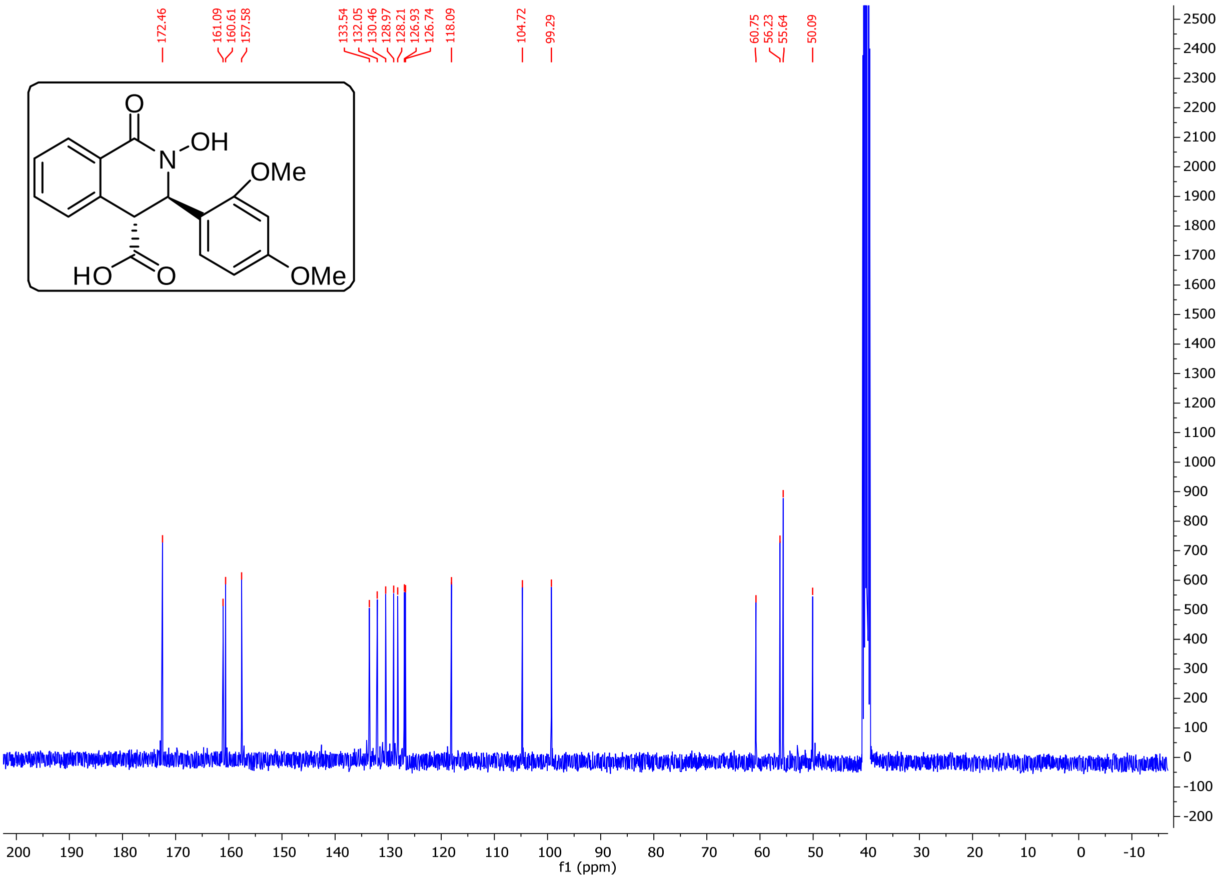

^1H and ^{13}C NMR of compound **7i**

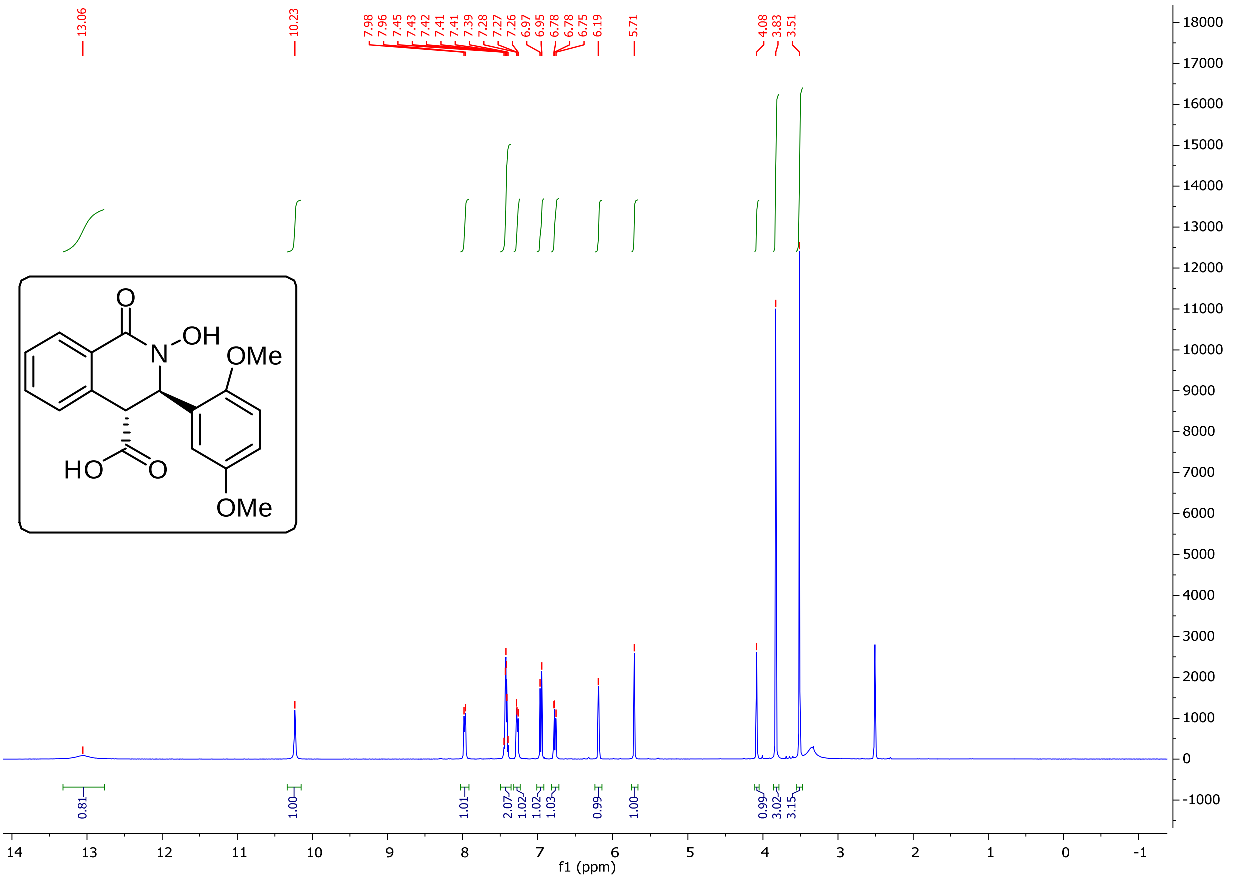

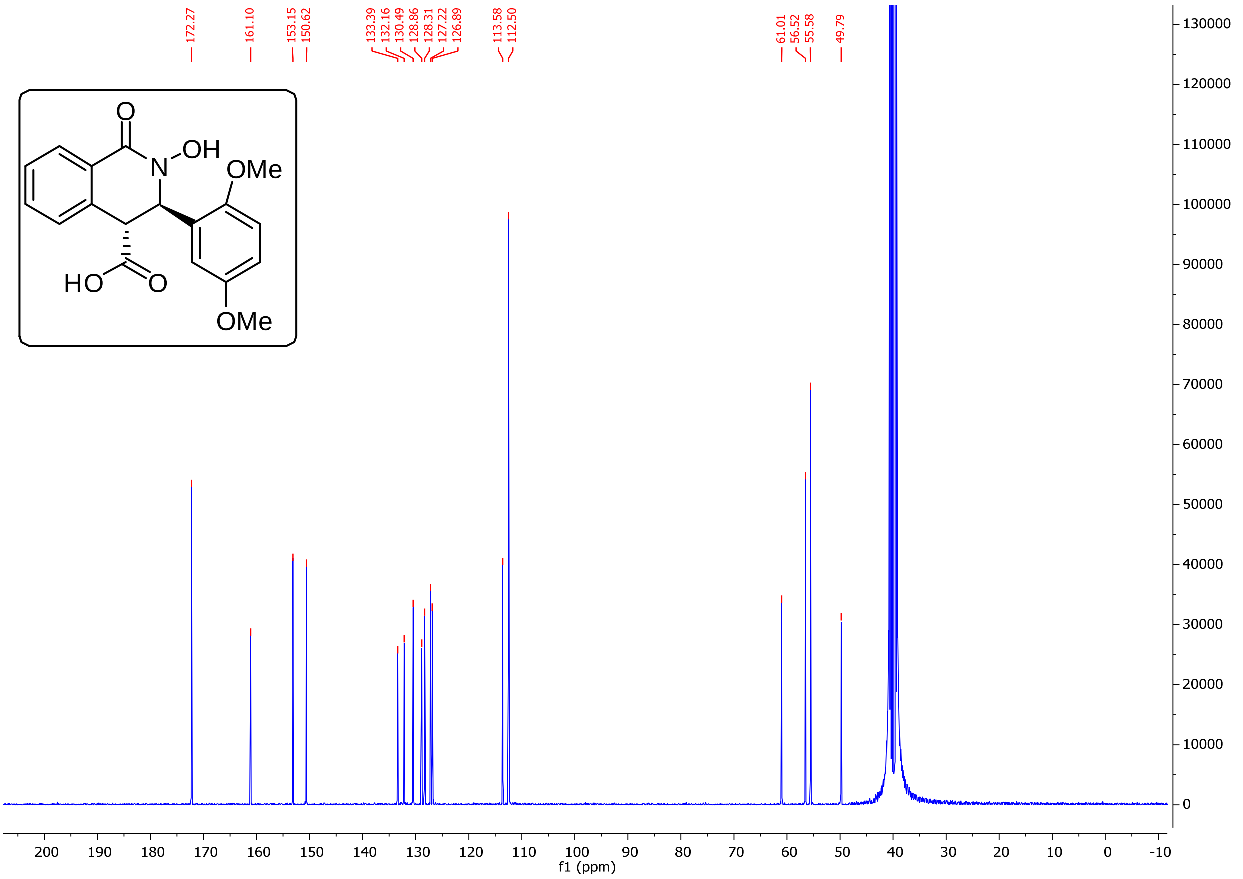

^{1}H and ^{13}C NMR of compound **7j**

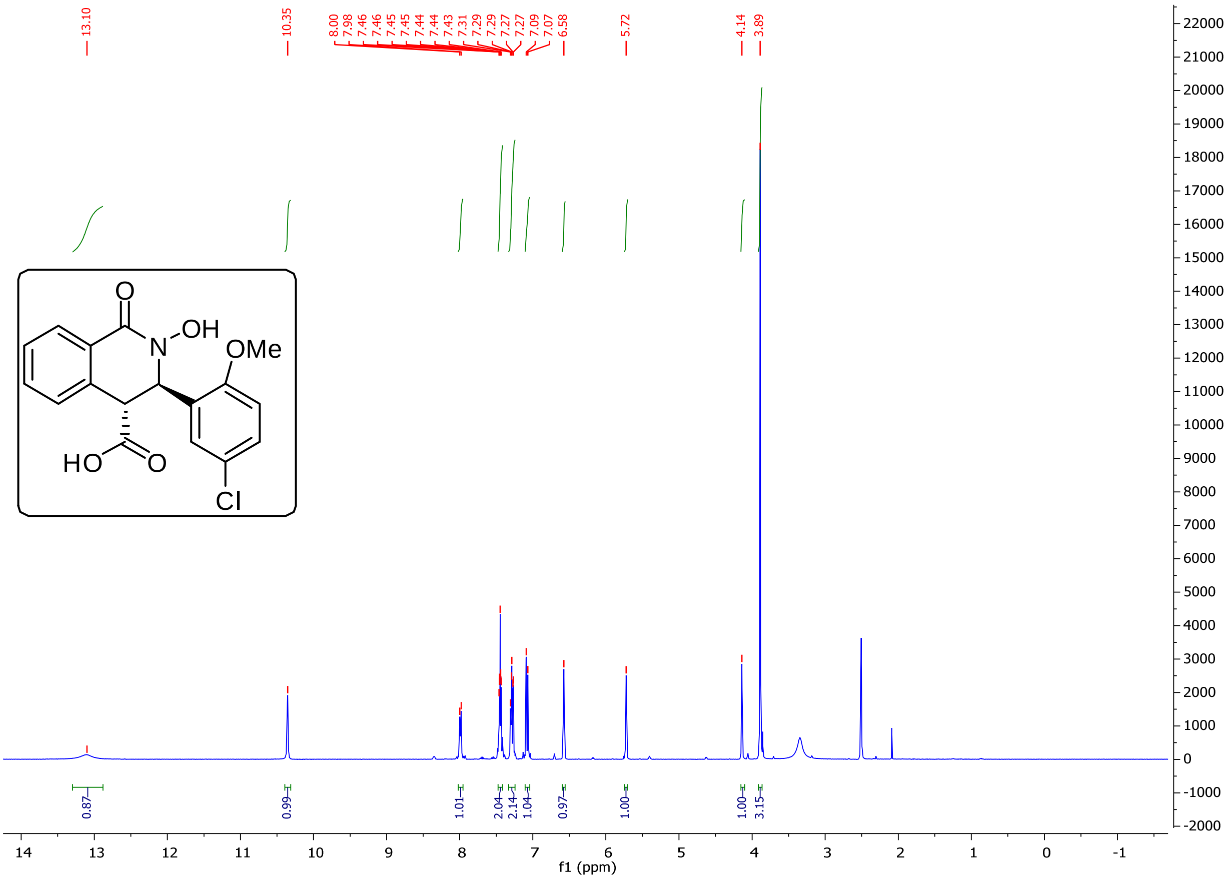

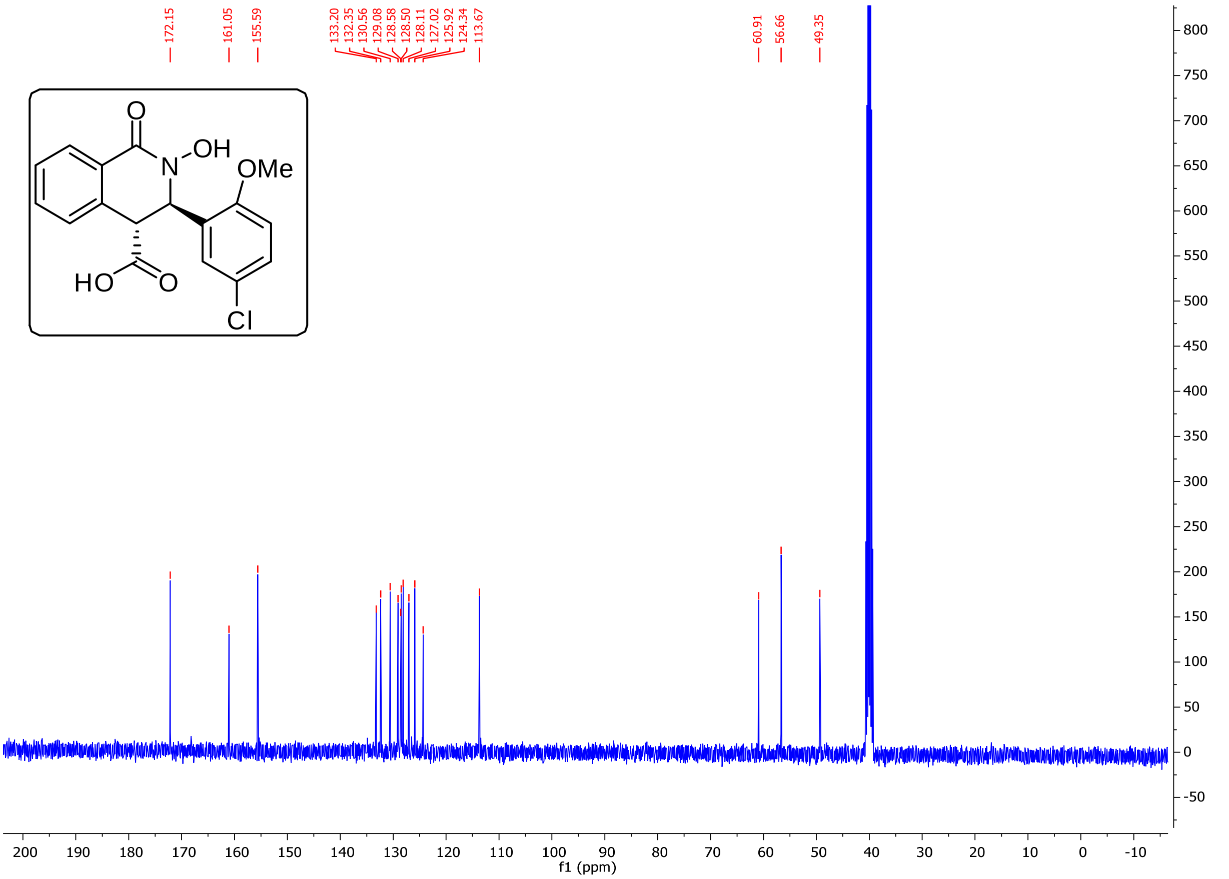

^{1}H and ^{13}C NMR of compound **7l**

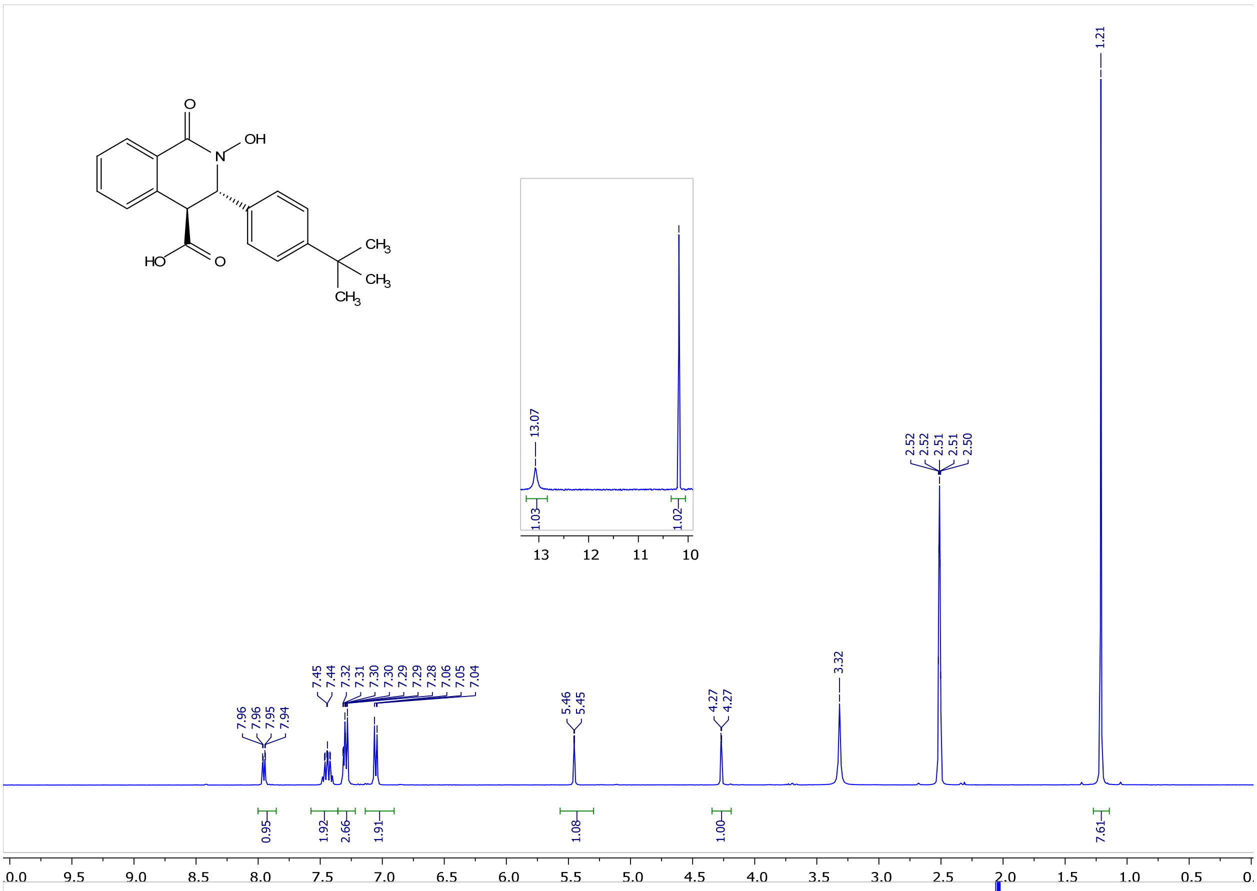

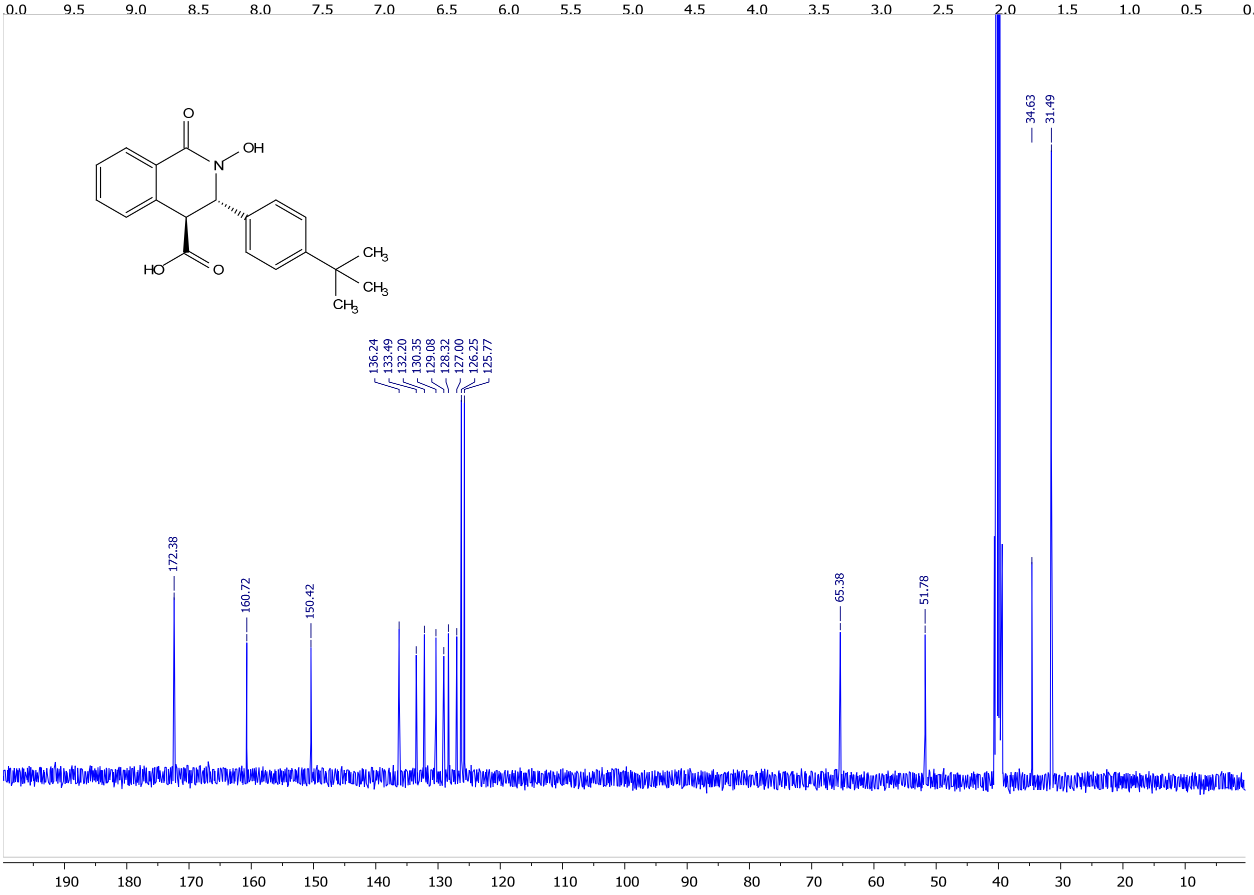

^{1}H and ^{13}C NMR of compound **8**

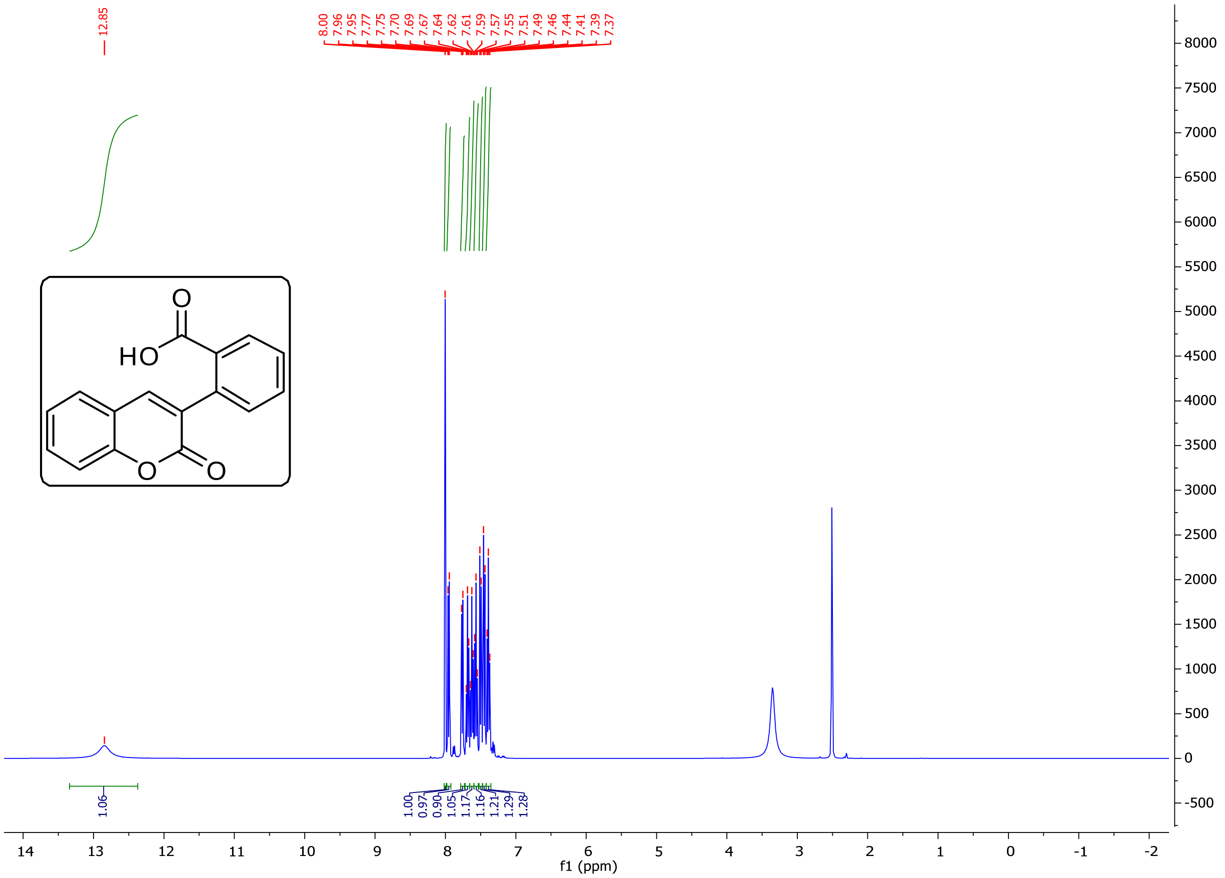

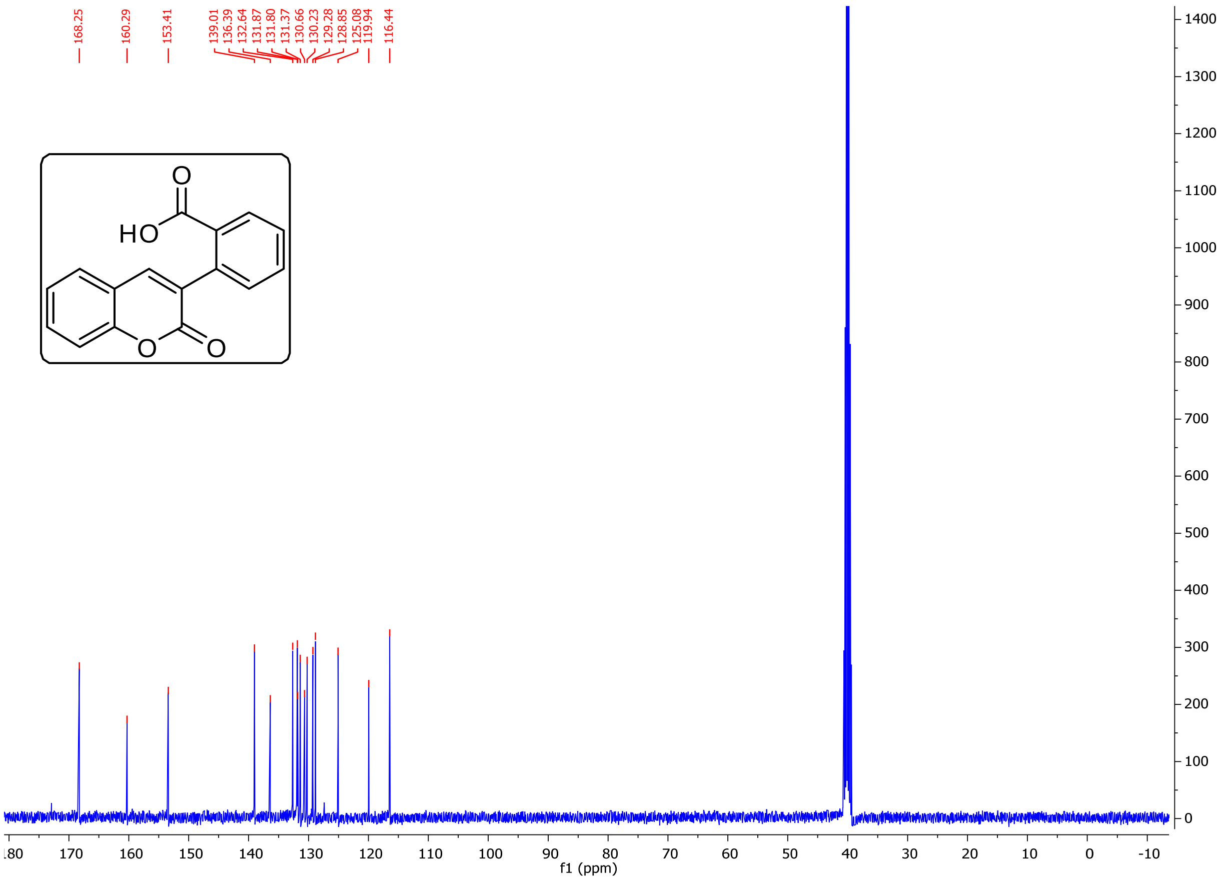

^{1}H and ^{13}C NMR of compound **9a**

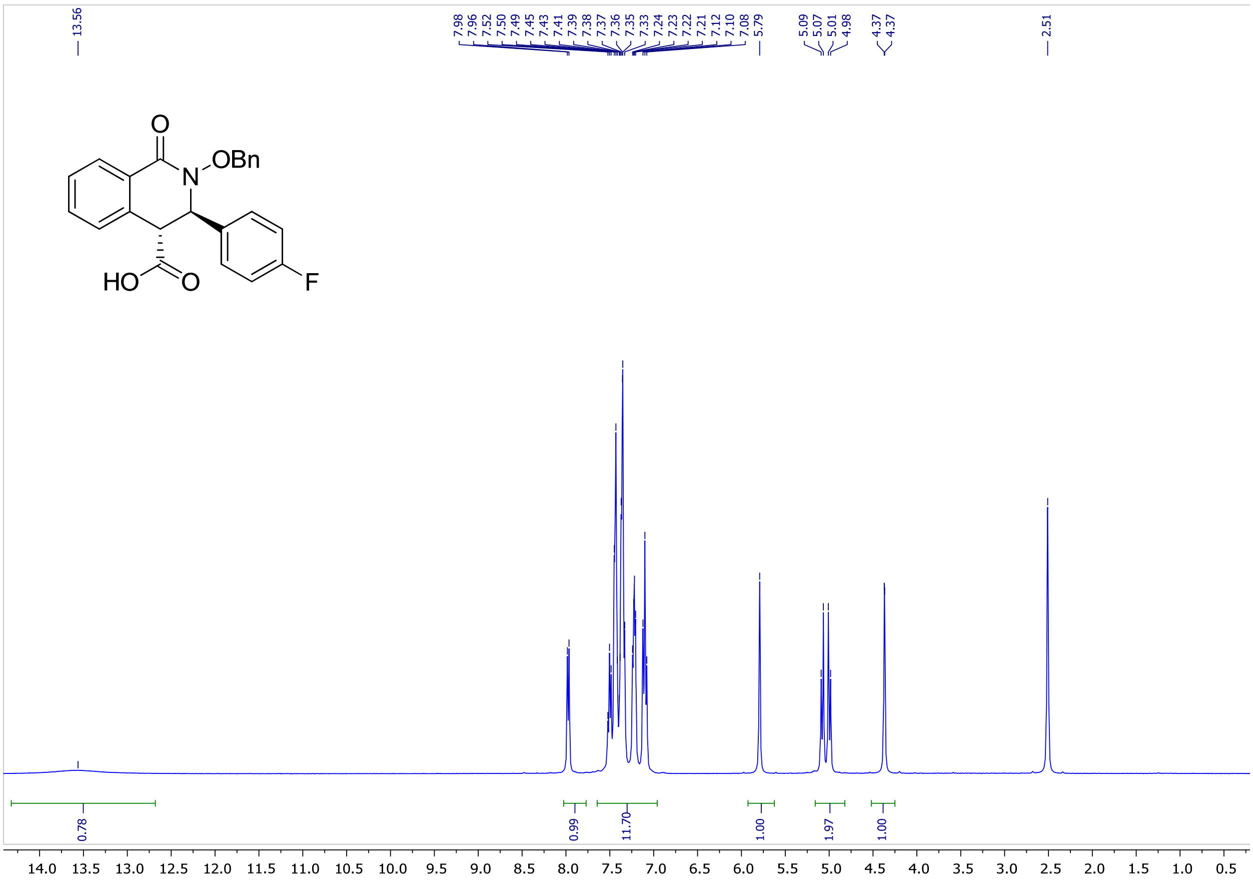

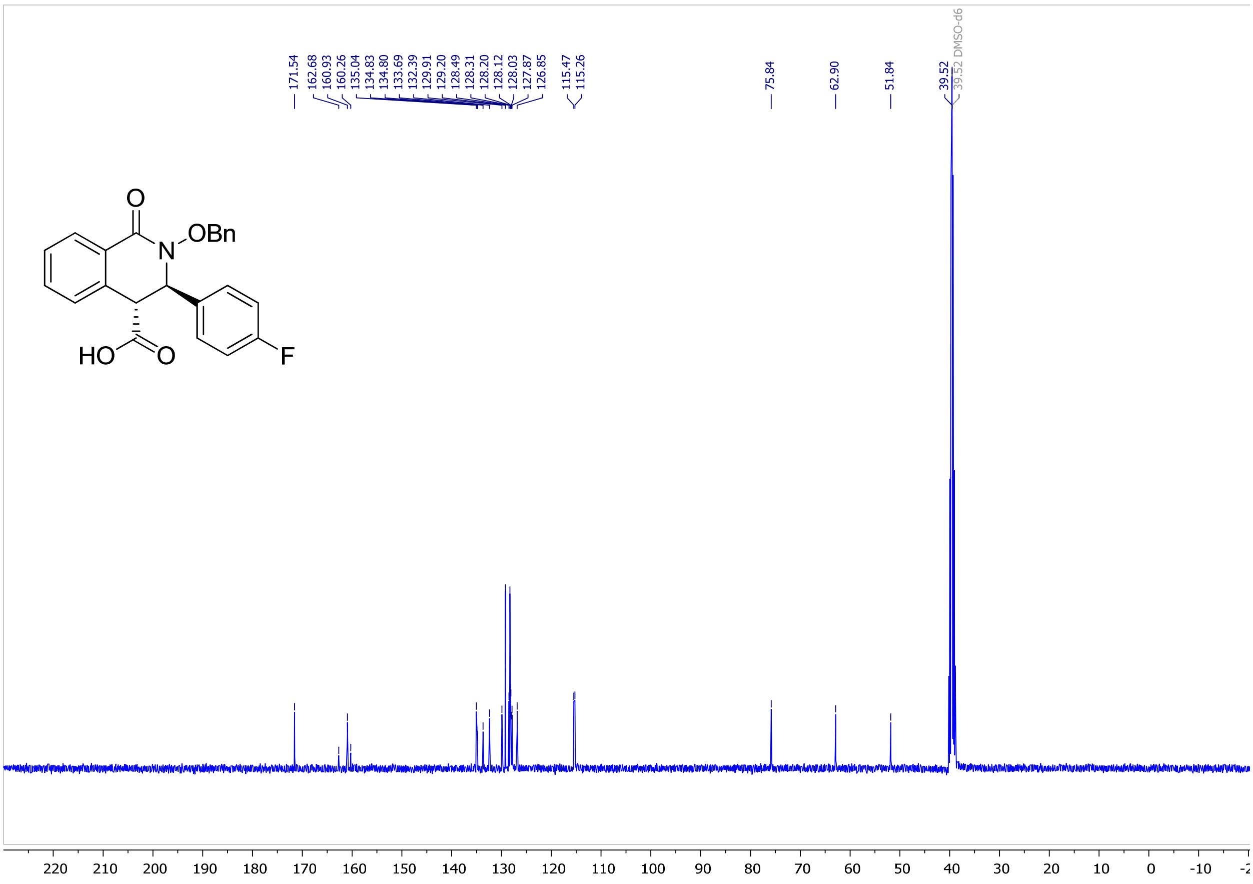

^{1}H and ^{13}C NMR of compound **9b**

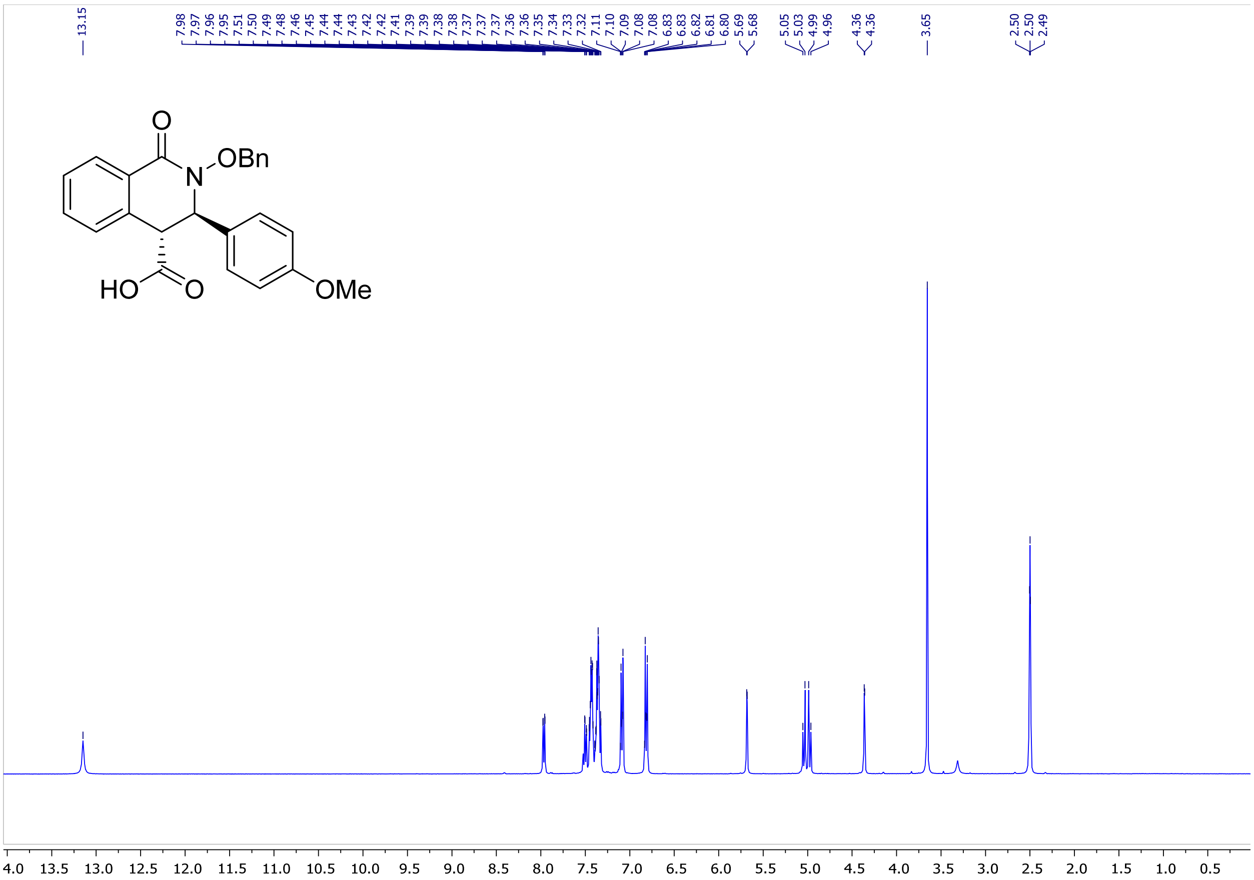

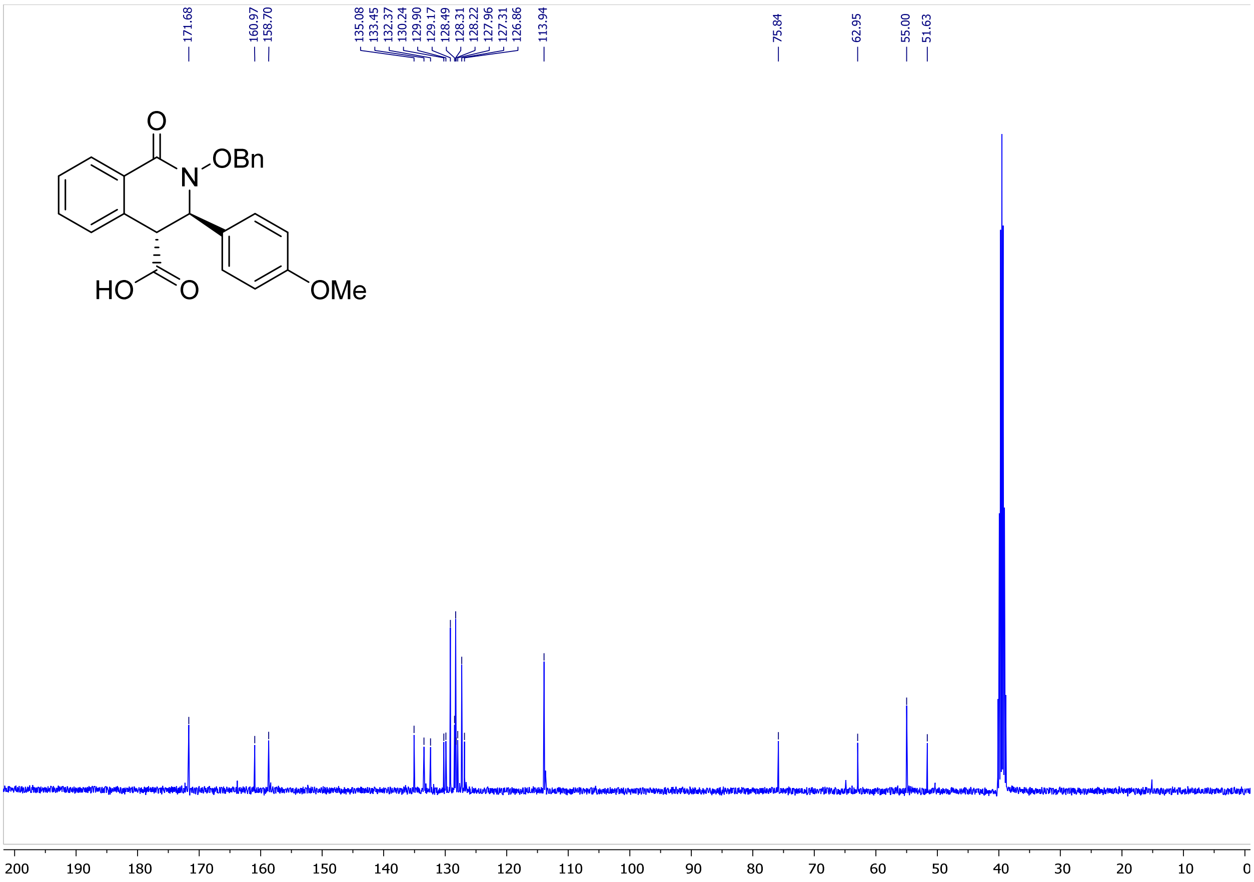

^{1}H and ^{13}C NMR of compound **9c**

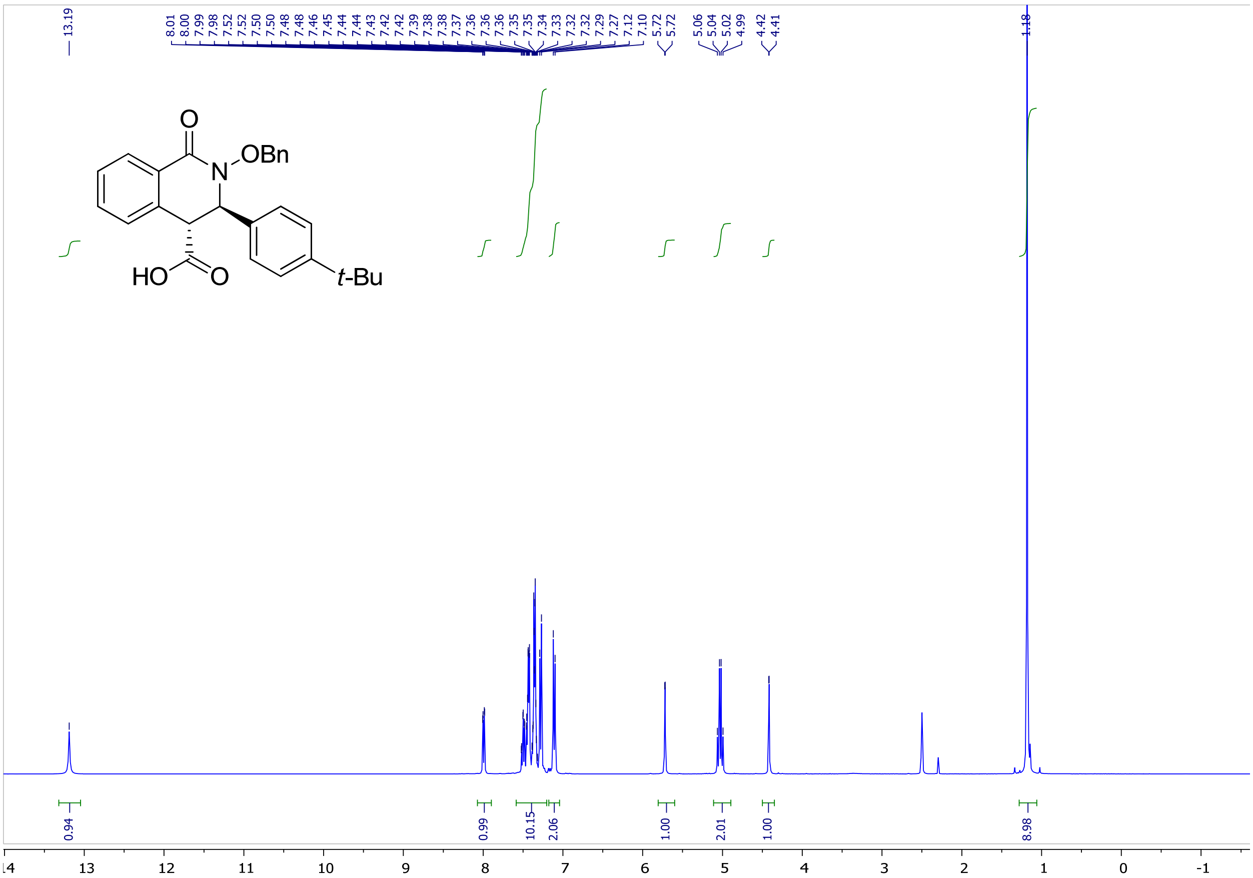

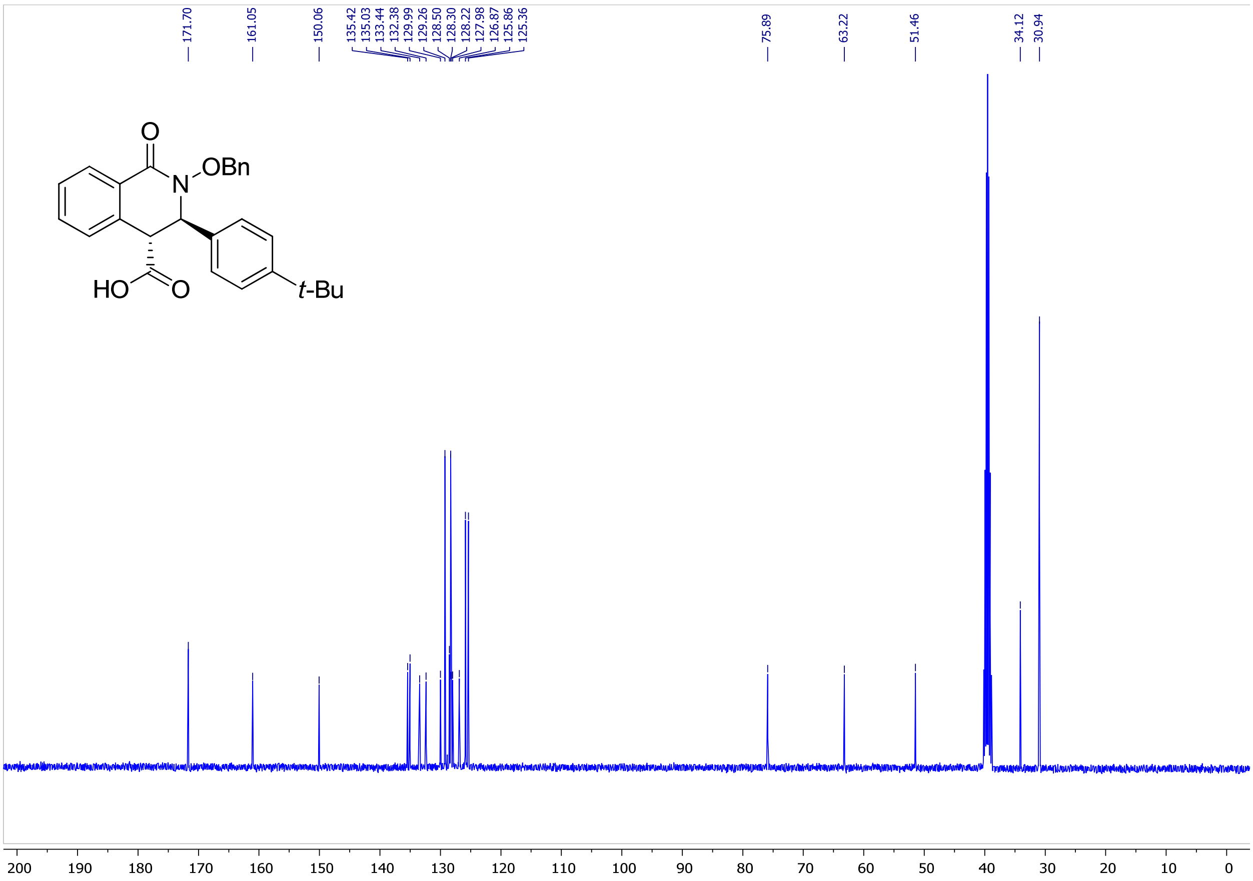

¹H and ¹³C NMR of compound **9d**

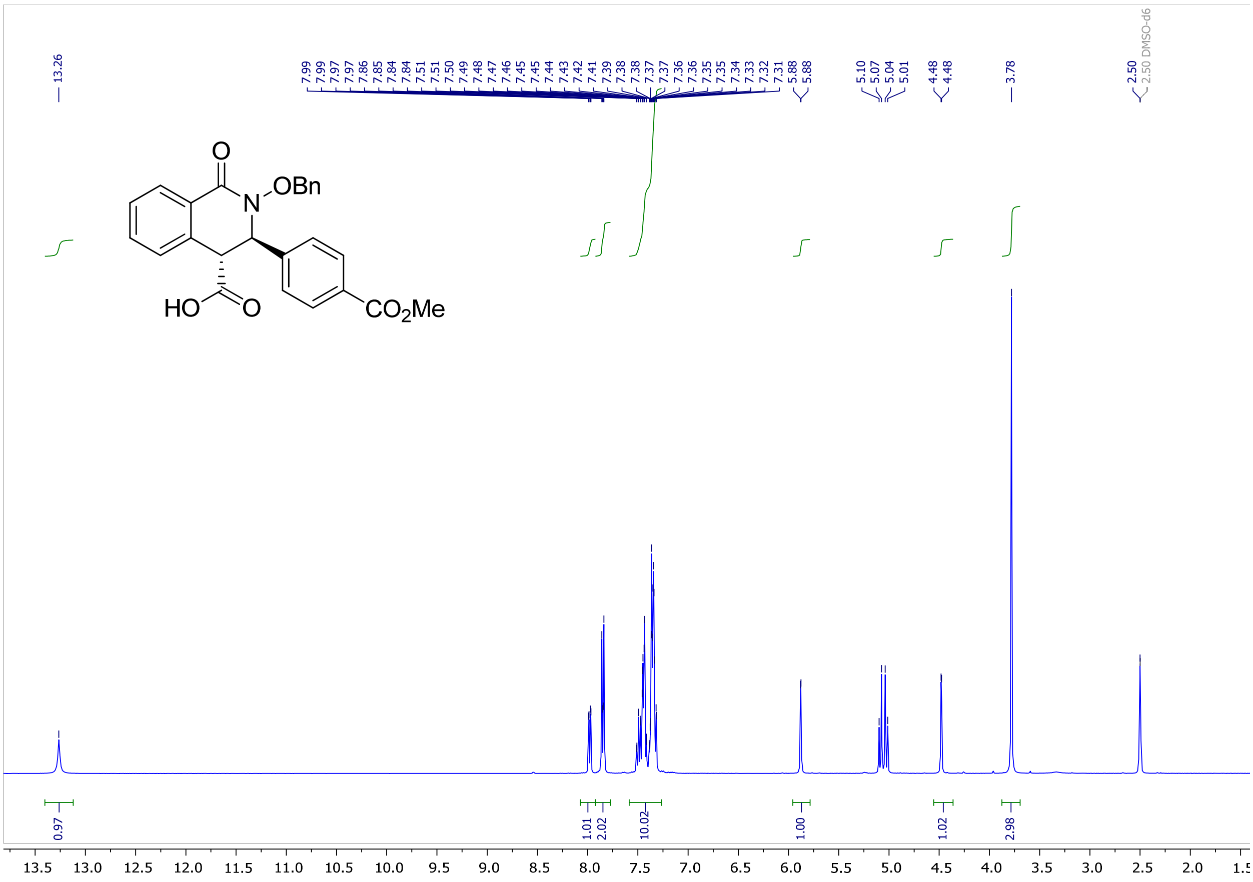

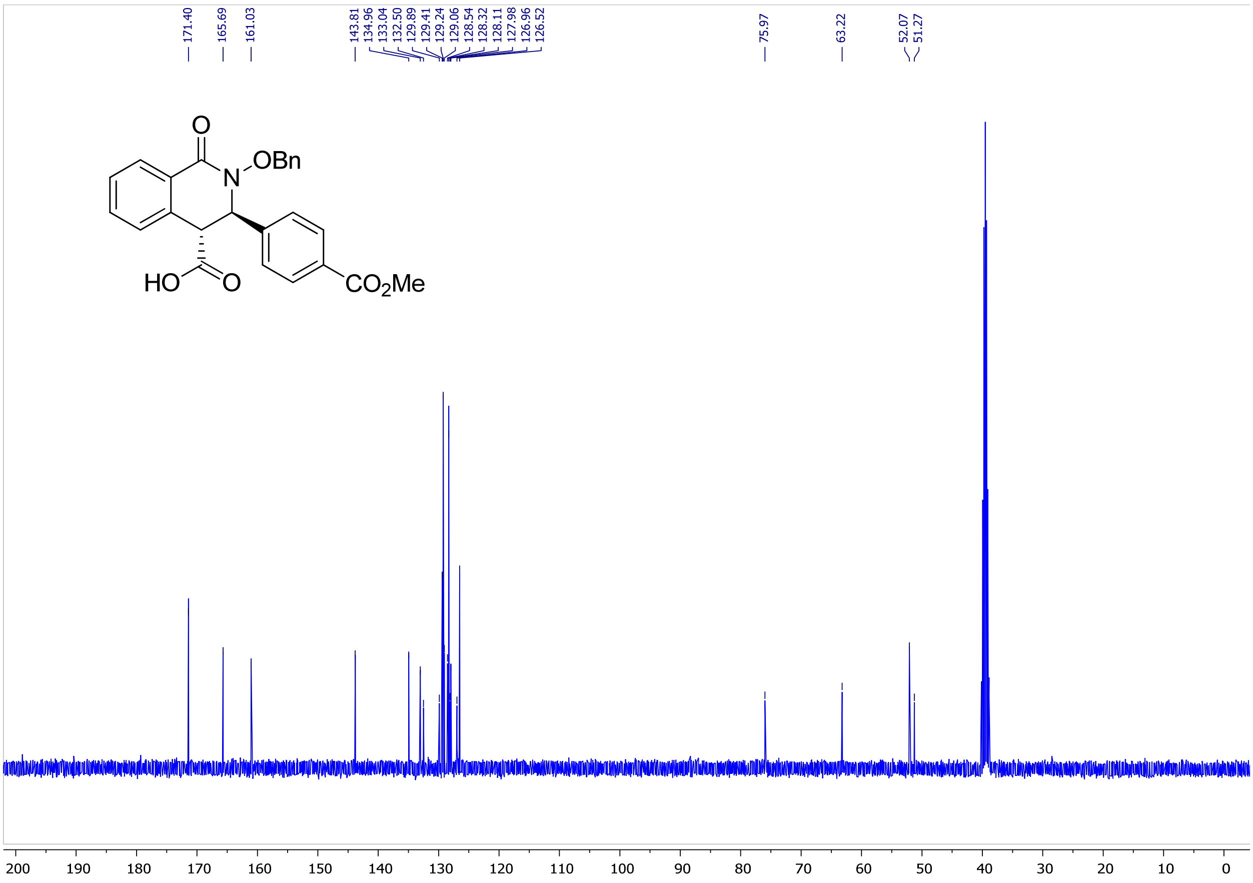

^{1}H and ^{13}C NMR of compound **9e**

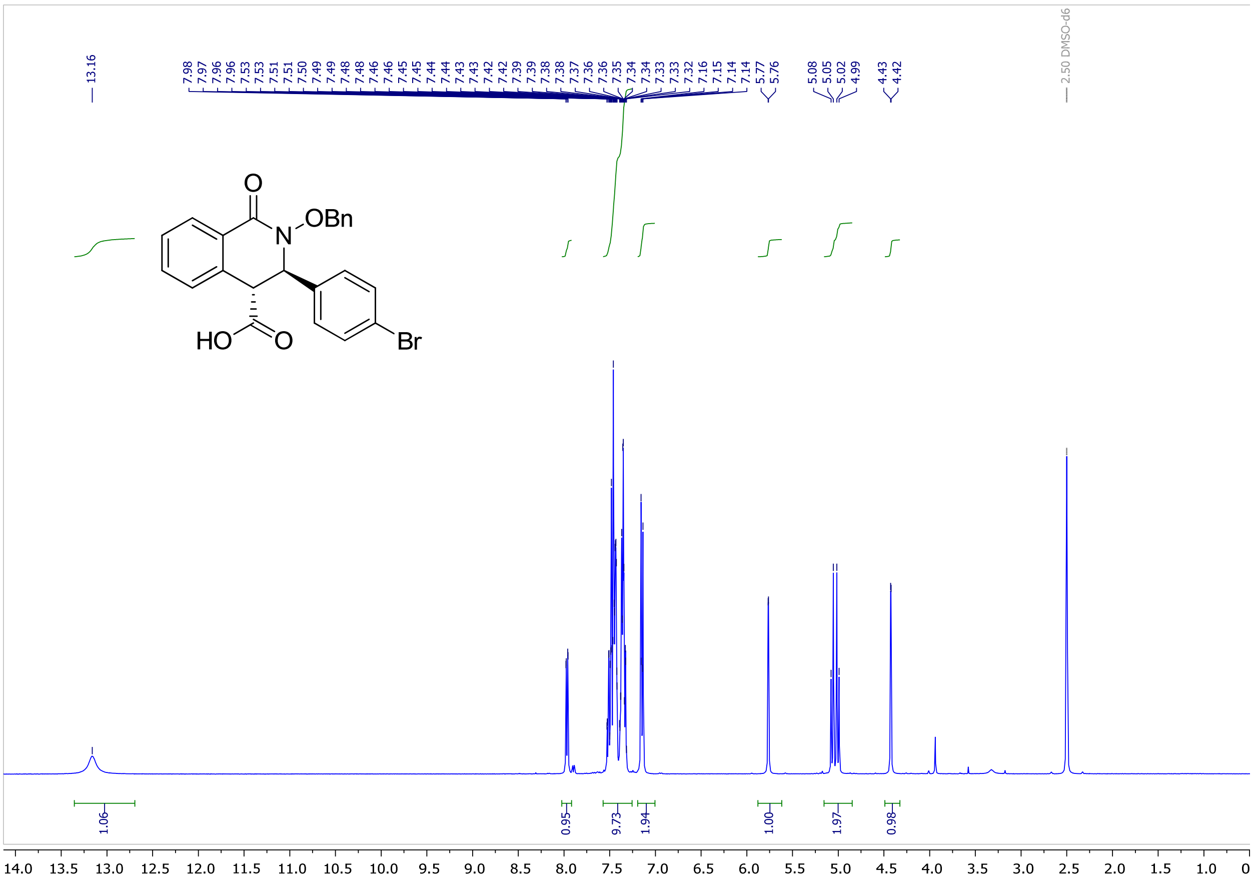

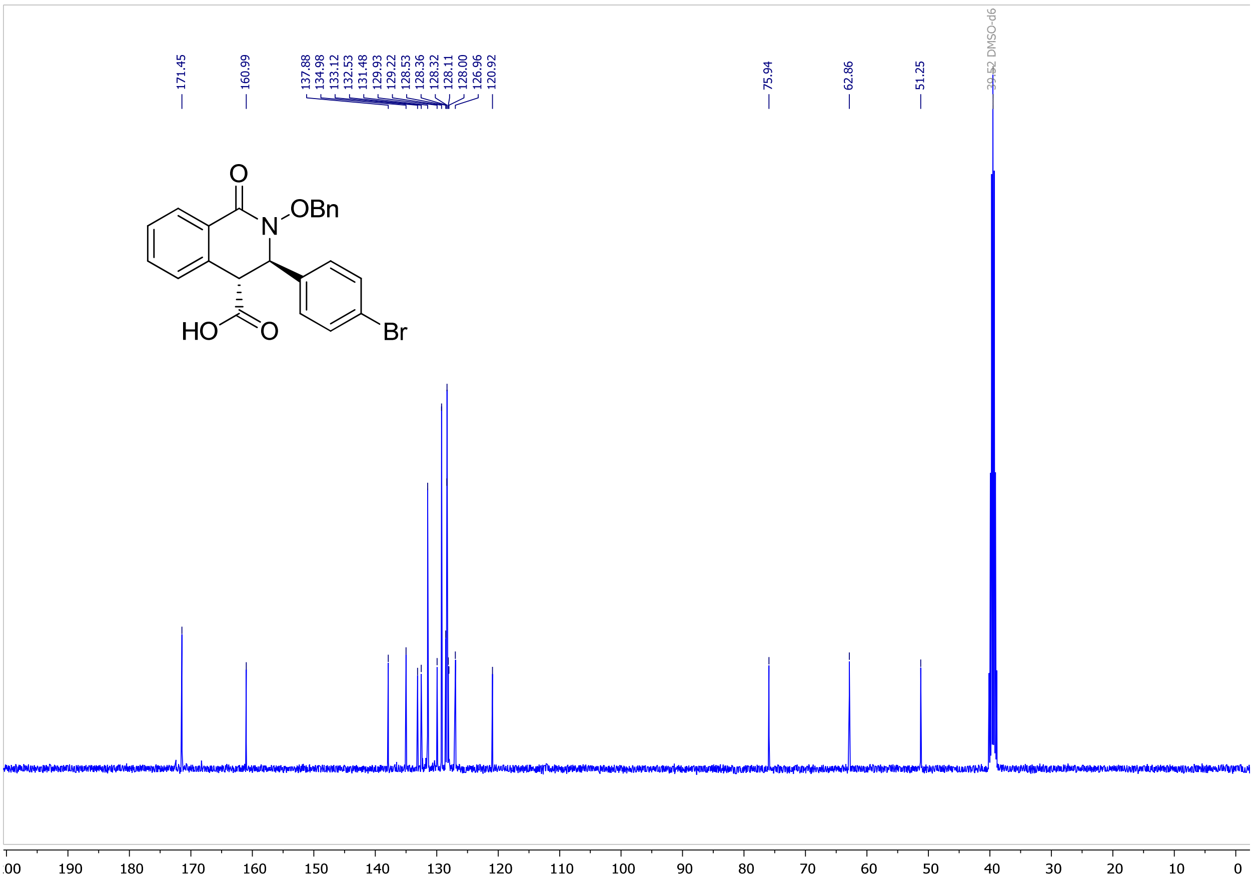

^{1}H and ^{13}C NMR of compound **9f**

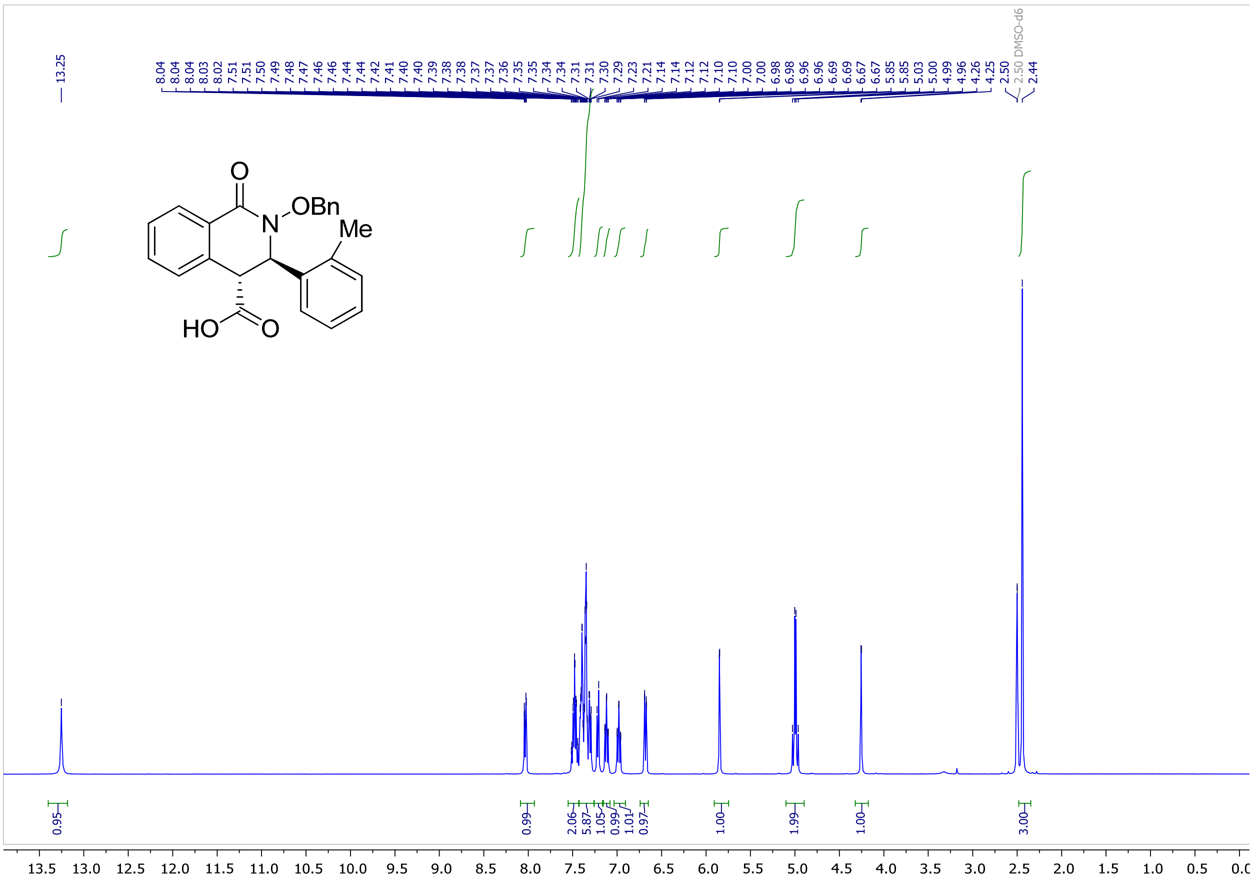

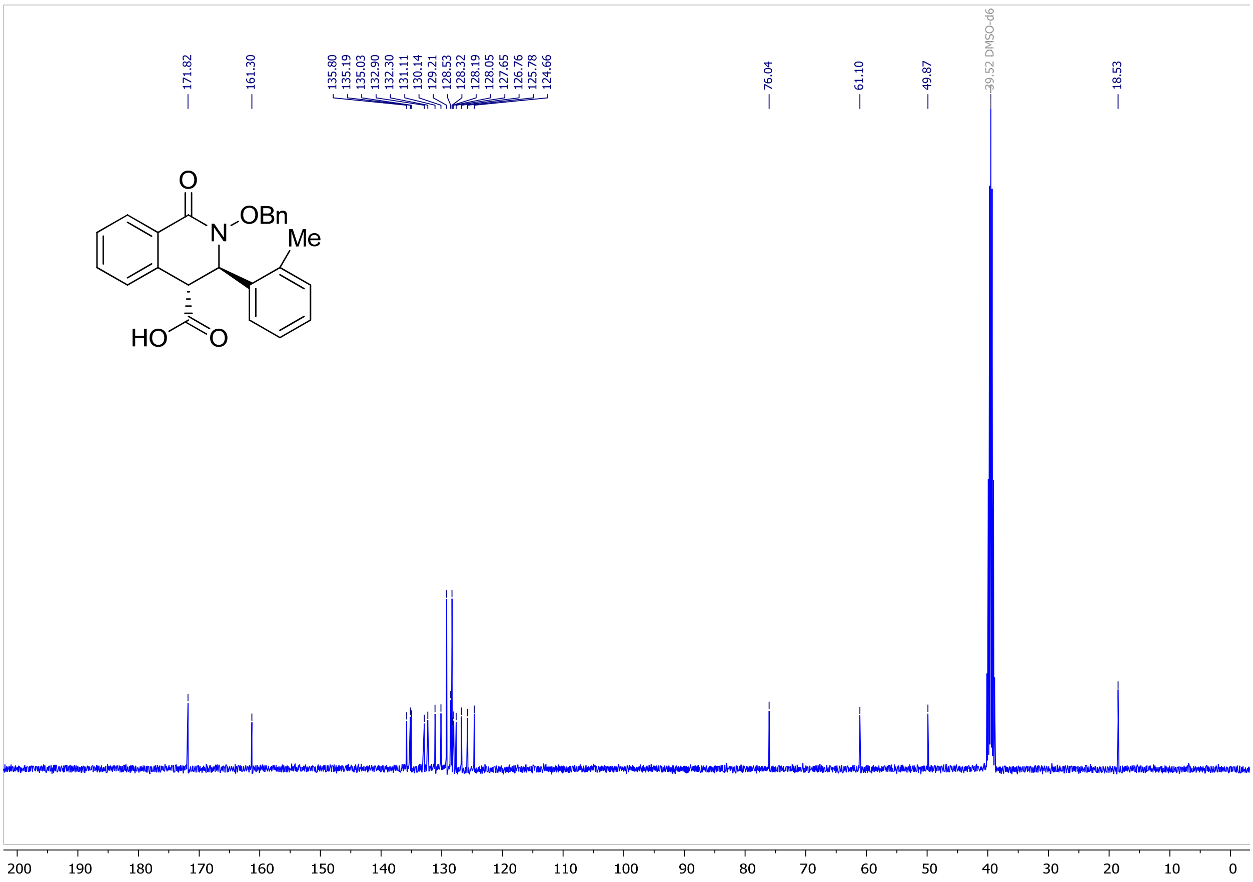

^{1}H and ^{13}C NMR of compound **9g**

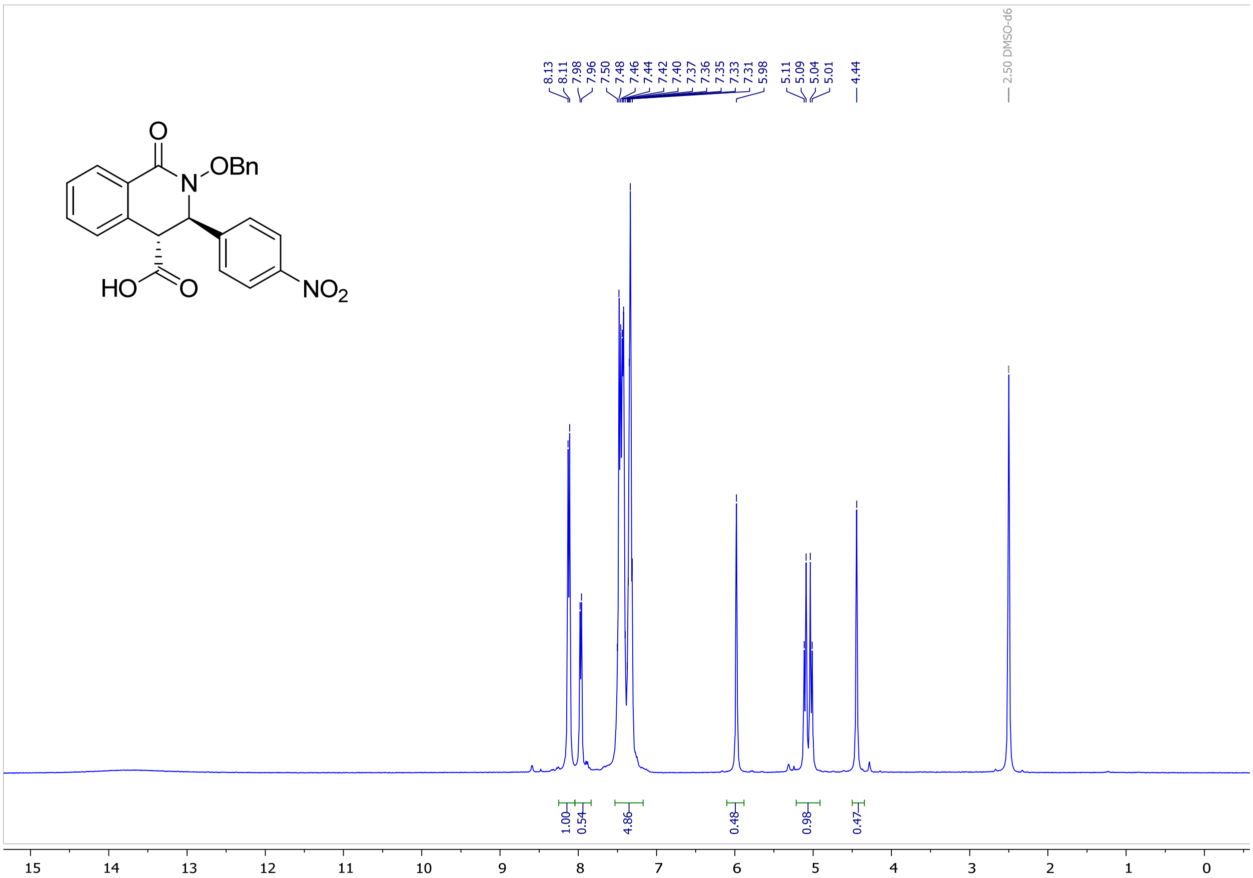

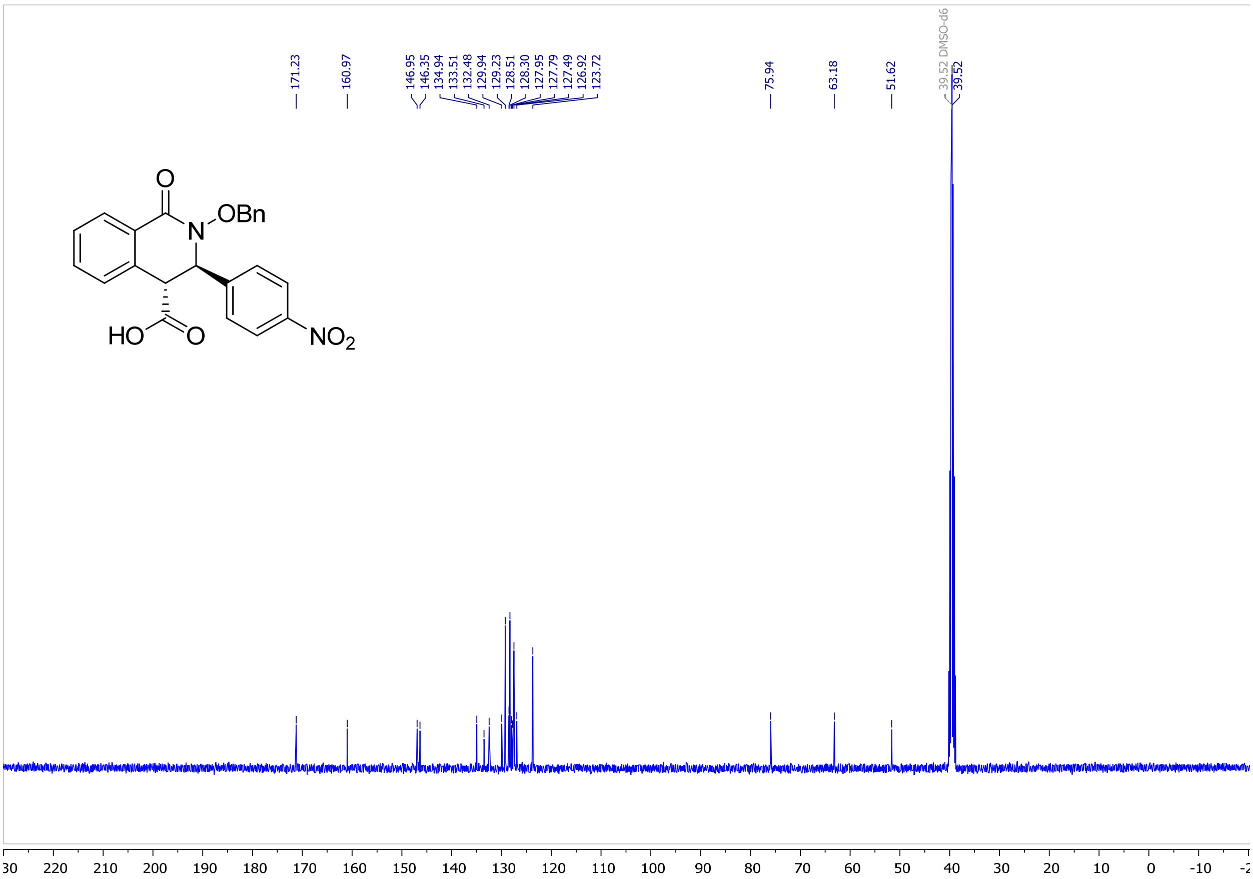

^{1}H and ^{13}C NMR of compound **10**

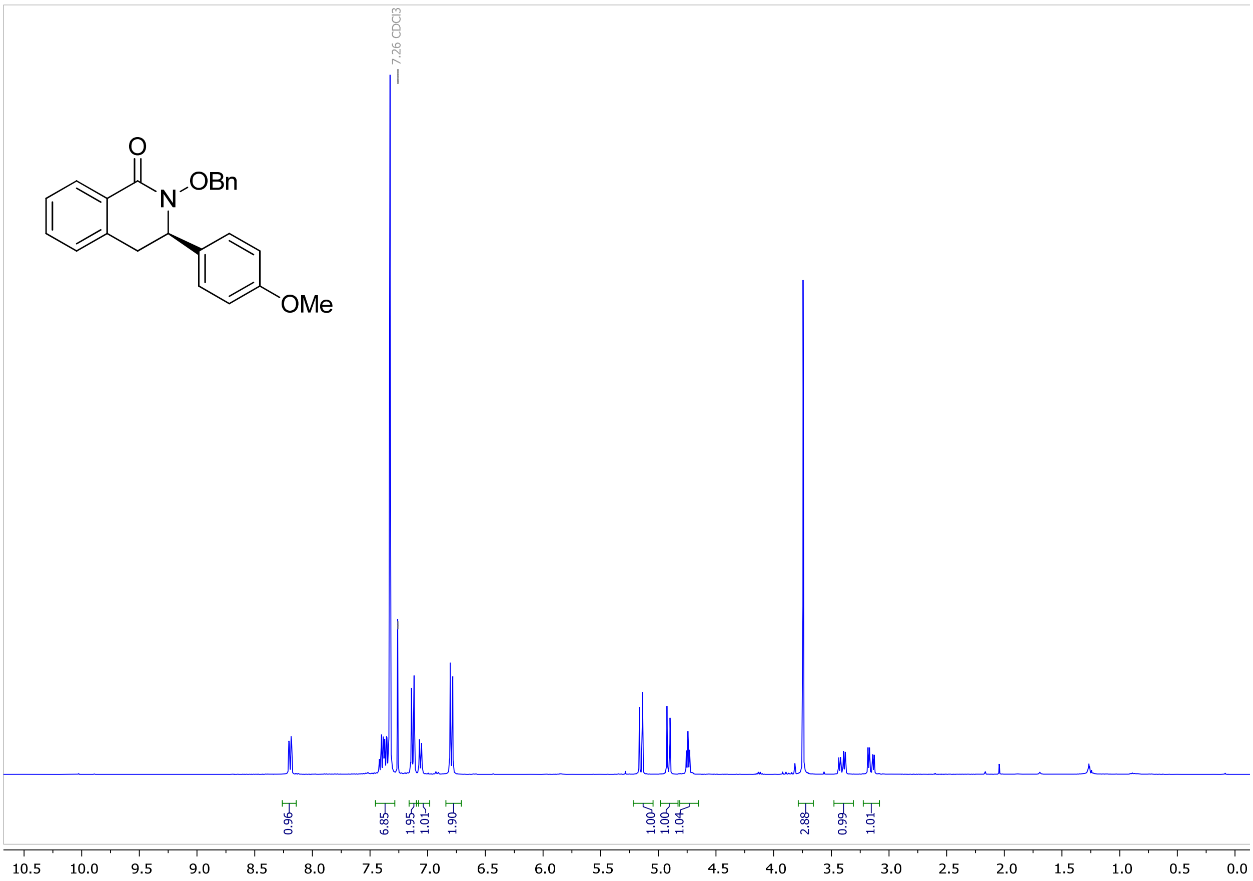

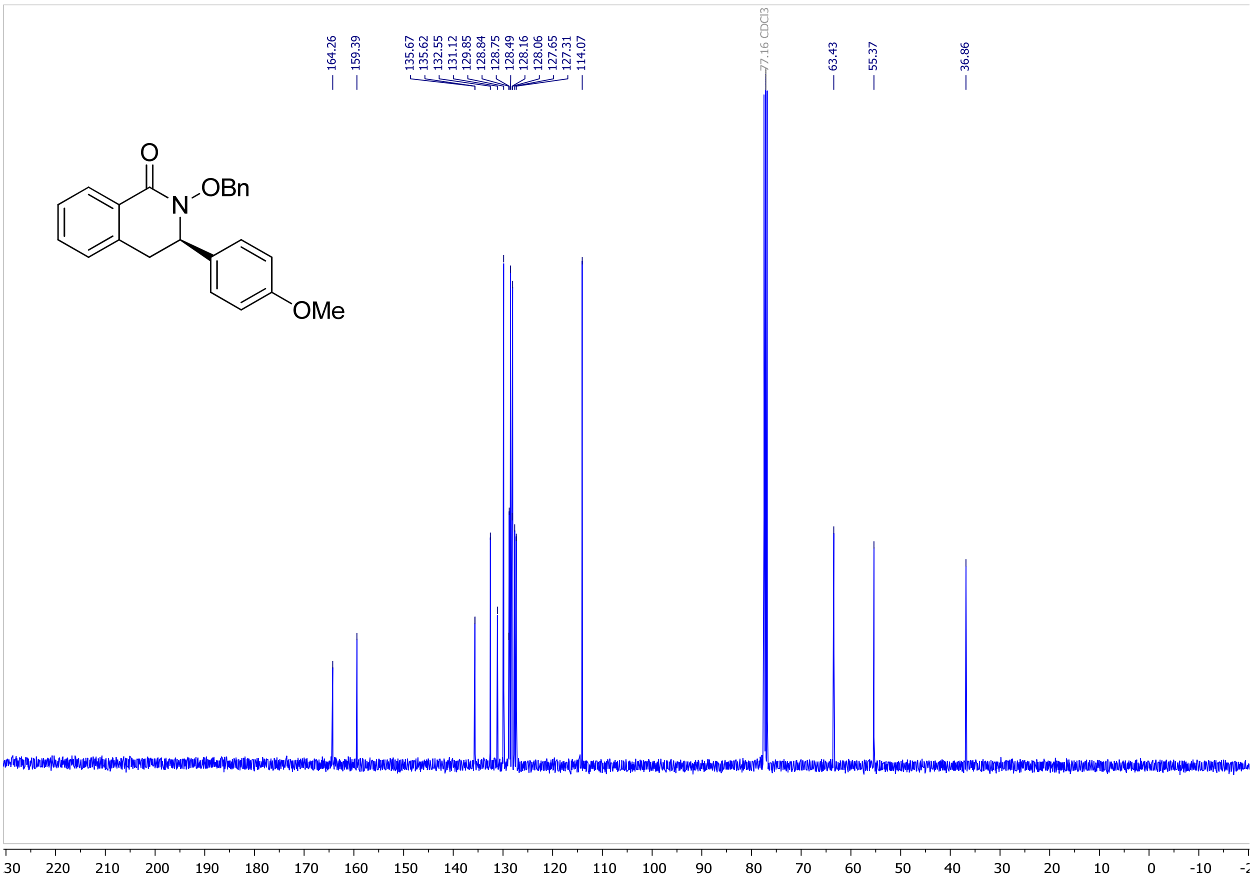

^{1}H and ^{13}C NMR of compound **11**

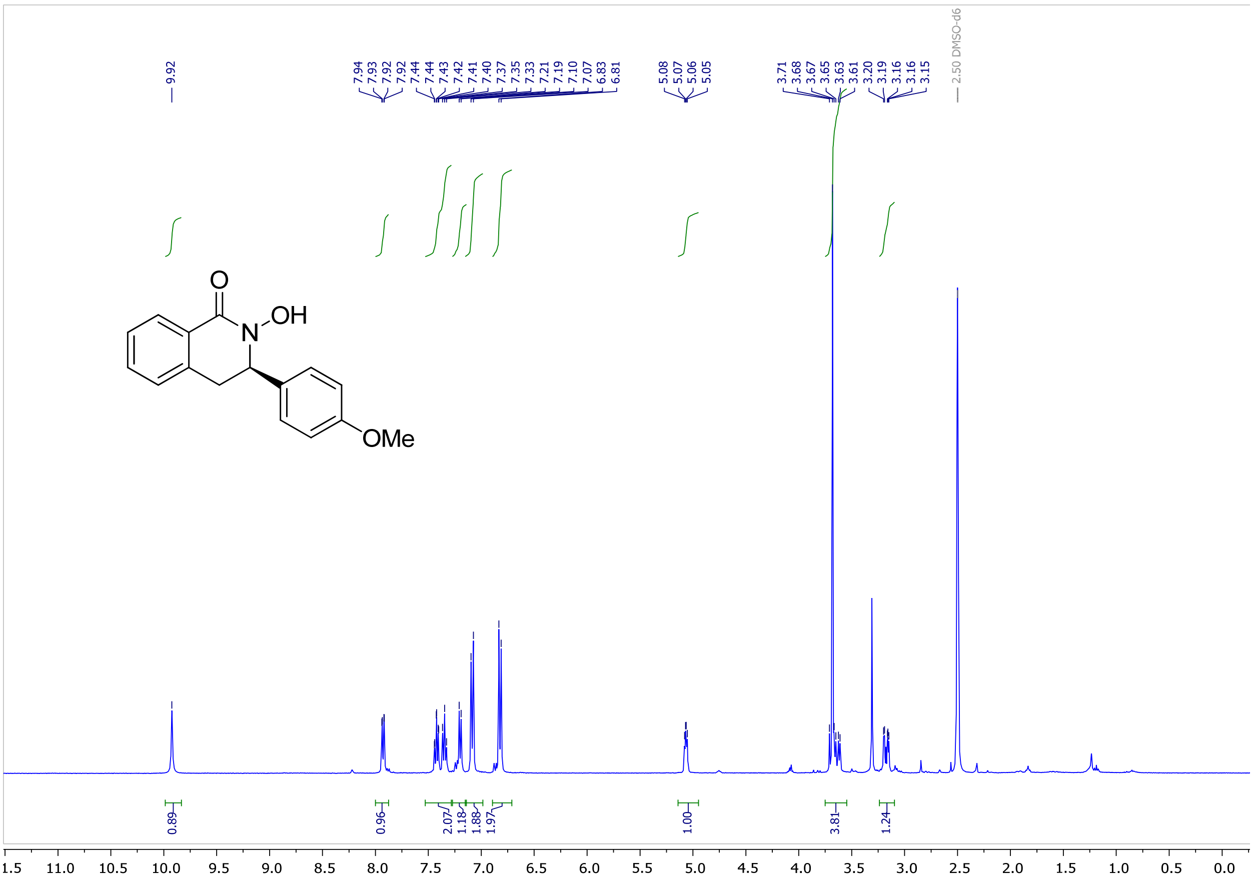

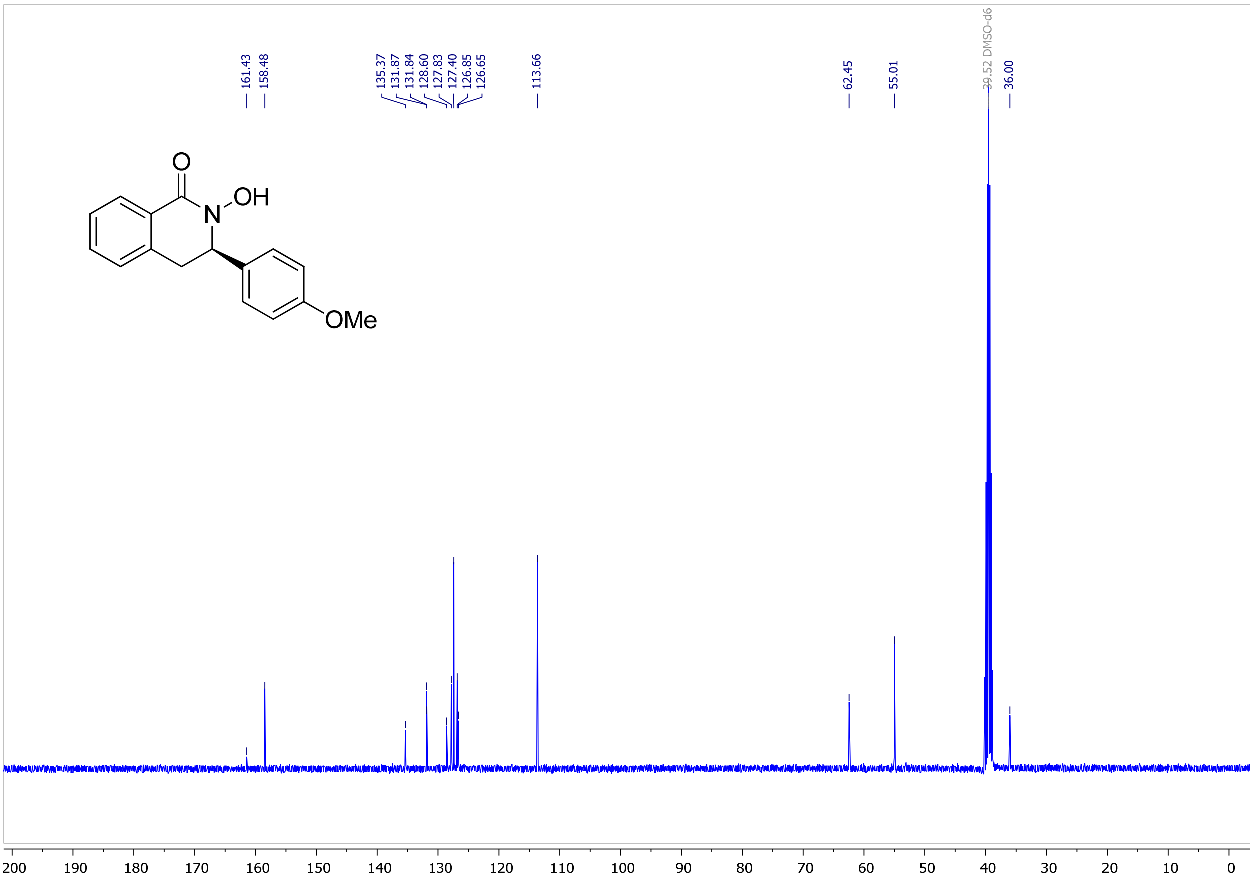

^{1}H and ^{13}C NMR of compound **12**

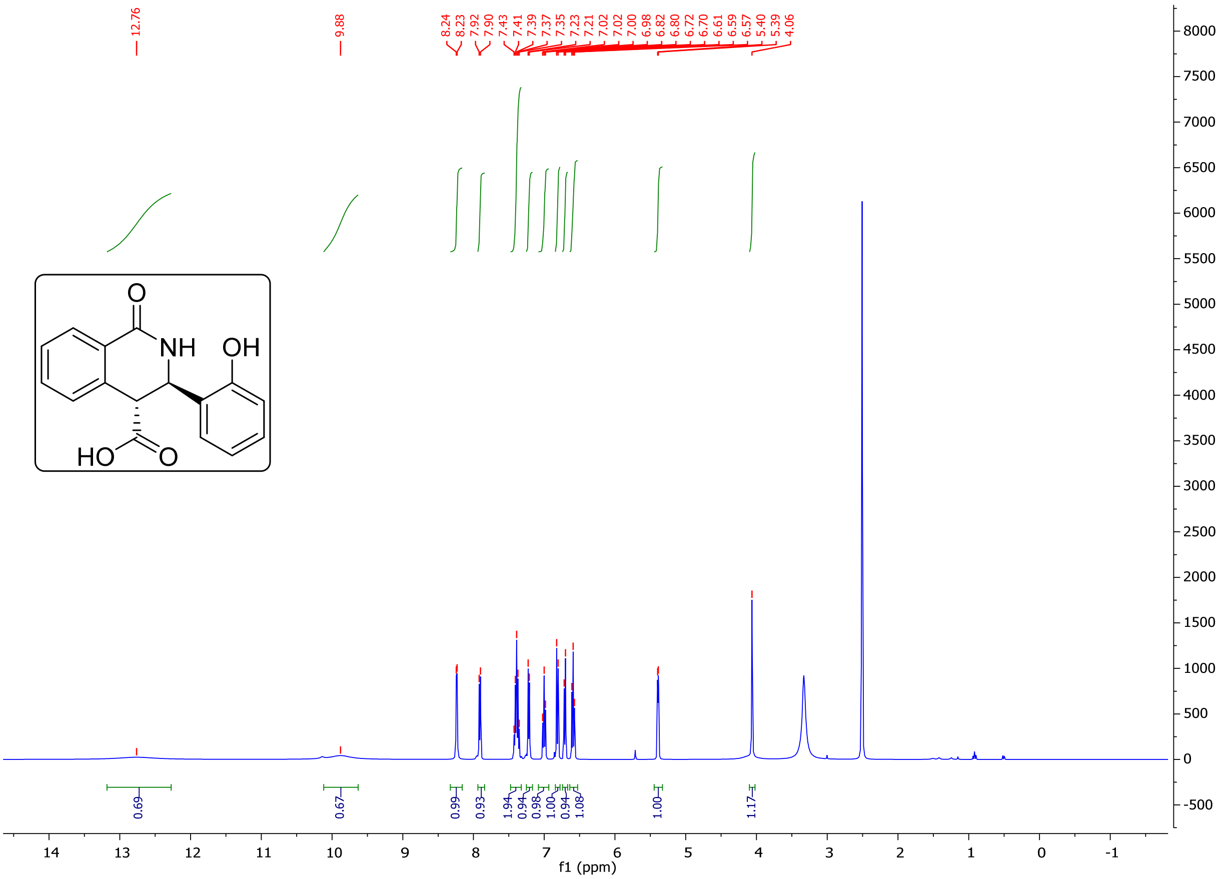

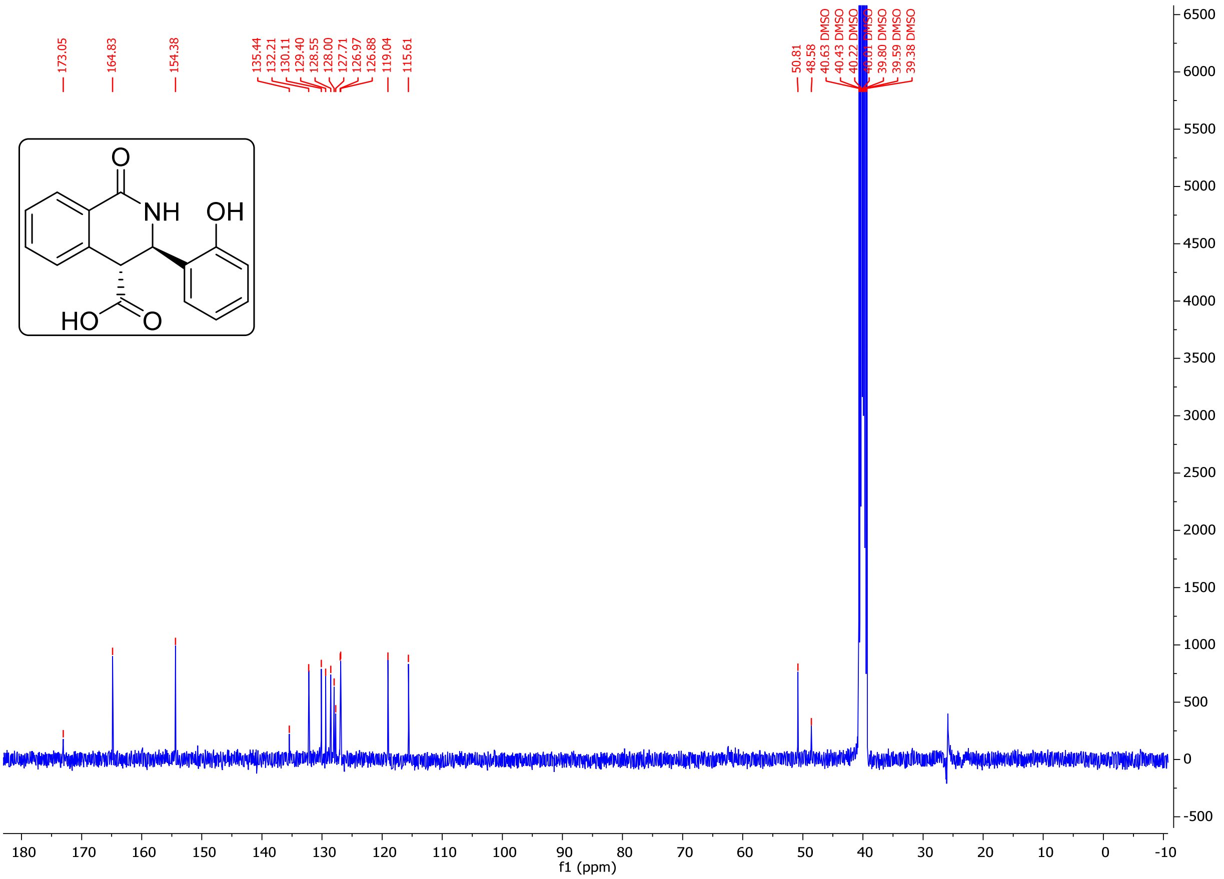

Electronic absorption spectra of 7d,f,g,h,j ligands recorded in MeOH (C = 5×10^{-5} M).

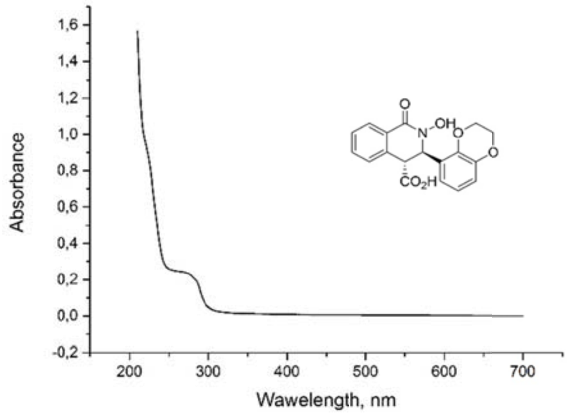

(a)

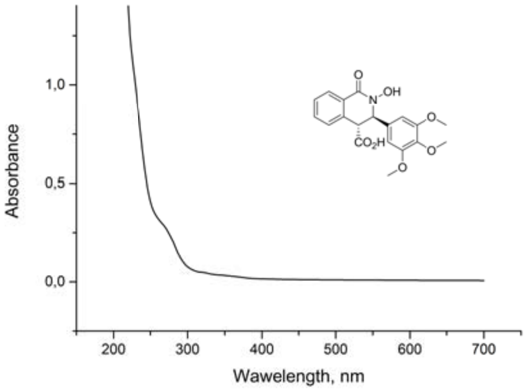

(b)

Figure S1. (a) UV-Vis spectrum of compound **7d**; (b) UV-Vis spectrum of compound **7f**;

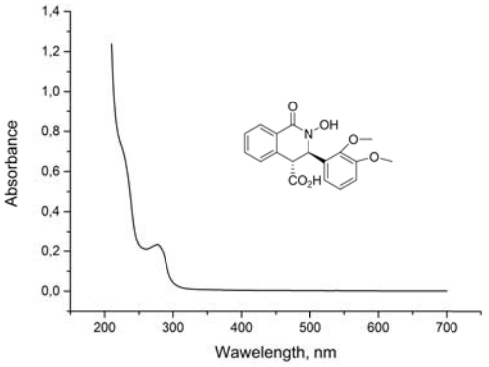

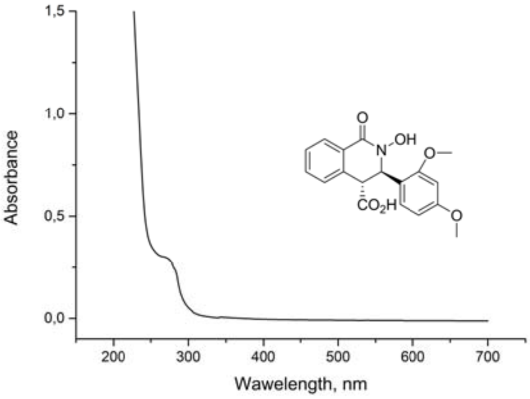

(a) **(b)**

Figure S2. (a) UV-Vis spectrum of compound **7g**; (b) UV-Vis spectrum of compound **7h**;

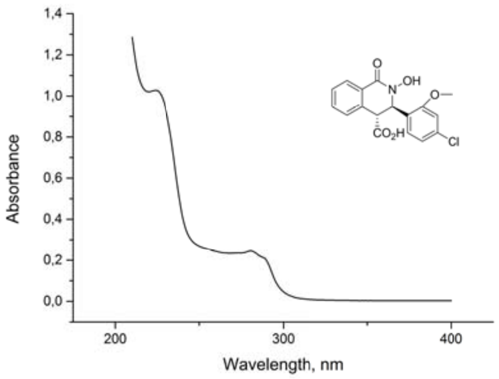

Figure S3. UV-Vis spectrum of compound **7j**

Electronic absorption spectra of 7d,f,g,h,j ligands (C_L = 5×10^{-4} M) in the presence of increasing concentration of Fe^{3+} (left; C_{Fe} = 0.25–5×10^{-4} M) and corresponding binding isotherms plotted at selected wavelengths (right; red asterisks – experimental data, black line – nonlinear curve fitting according to eqn.3, Materials and Methods).

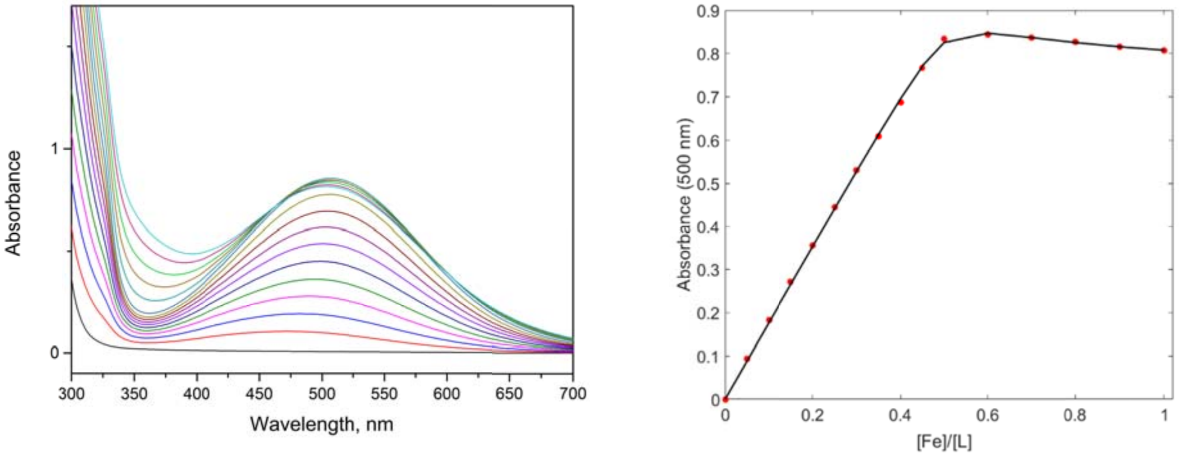

(a) **(b)**

Figure S4. (a) Changes in UV-Vis spectrum of compound **7d** upon addition of $Fe(NO_3)_3$; (b) corresponding mole ratio plot (500 nm), **7d**+Fe^{3+}

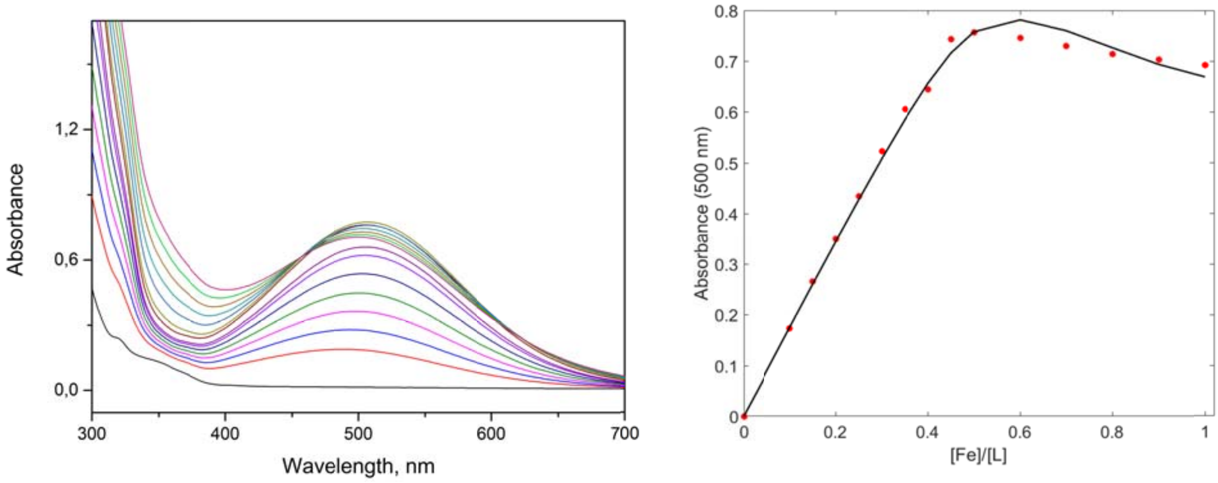

(a) **(b)**

Figure S5. (a) Changes in UV-Vis spectrum of compound **7f** upon addition of $Fe(NO_3)_3$; (b) corresponding mole ratio plot (500 nm), **7f**+Fe^{3+}

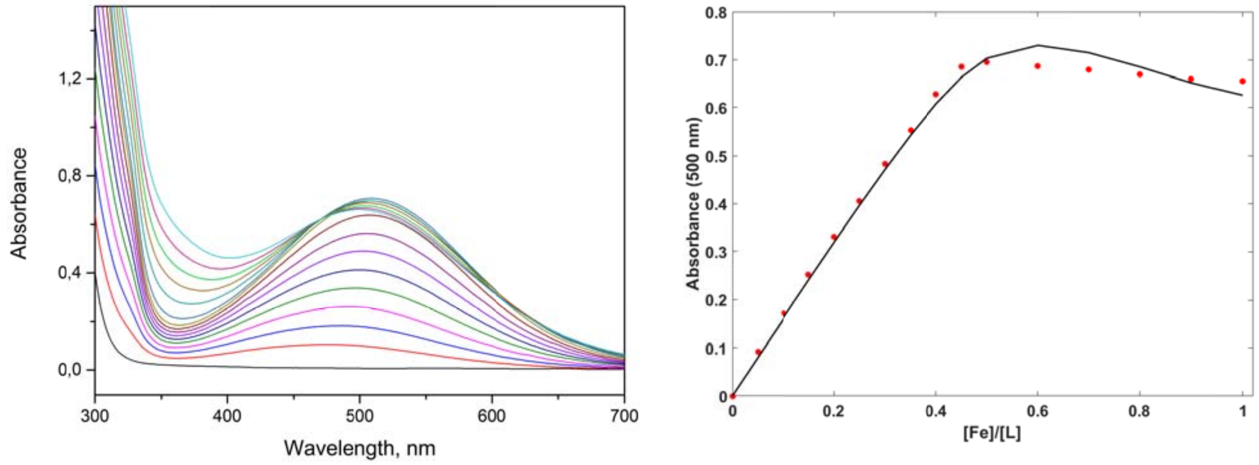

(b)

(a)

Figure S6. (a) Changes in UV-Vis spectrum of compound **7g** upon addition of $Fe(NO_3)_3$; (b) corresponding mole ratio plot (500 nm), **7g**+Fe^{3+}

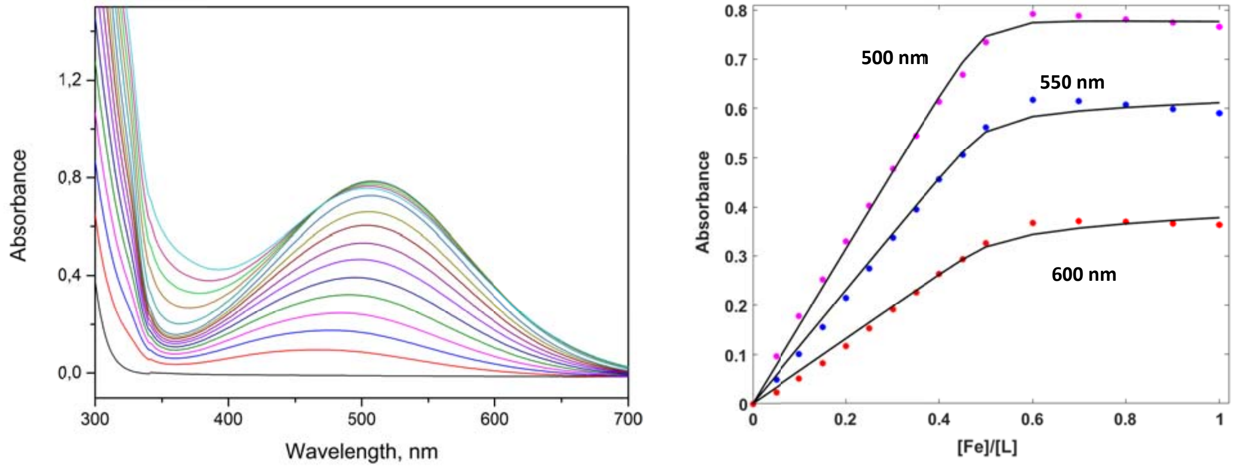

(a) (b)

Figure S7. (a) Changes in UV-Vis spectrum of compound **7h** upon addition of $Fe(NO_3)_3$; (b) corresponding mole ratio plots (500, 550, 600 nm), **7h**+Fe^{3+}

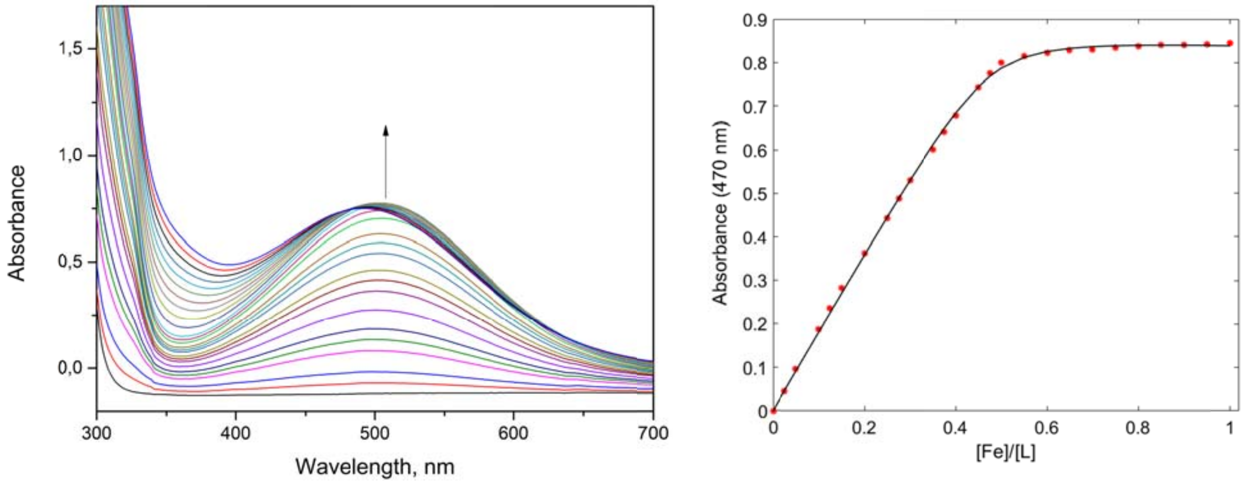

(a) (b)

Figure S8. (a) Changes in UV-Vis spectrum of compound **7j** upon addition of $Fe(NO_3)_3$; (b) corresponding mole ratio plot (470 nm), **7j**+Fe^{3+}
